# Sexuality and its effects on older adults’ depressive symptoms and quality of life

**DOI:** 10.1590/0034-7167-2021-0645

**Published:** 2023-02-06

**Authors:** Edison Vitório de Souza, Diego Pires Cruz, Lais Reis Siqueira, Uanderson Silva Pirôpo, Randson Souza Rosa, Benedito Fernandes da Silva, Namie Okino Sawada

**Affiliations:** I Universidade de São Paulo. Ribeirão Preto, São Paulo, Brazil.; II Universidade Estadual do Sudoeste da Bahia. Jequié, Bahia, Brazil.; III Universidade Federal de Alfenas. Alfenas, Minas Gerais, Brazil.; IV Universidade Estadual de Feira de Santana. Feira de Santana, Bahia, Brazil.

**Keywords:** Aged, Sexuality, Depression, Quality of Life, Health of the Elderly, Anciano, Sexualidad, Depresión, Calidad de Vida, Salud del Anciano, Idoso, Sexualidade, Depressão, Qualidade de Vida, Saúde do Idoso

## Abstract

**Objectives:**

to analyze the effects of sexuality on depressive symptoms and quality of life in older adults.

**Methods:**

a cross-sectional and analytical study, developed with 596 older adults, who completed four instruments for data collection. Data were analyzed using the Kruskal-Wallis test and Structural Equation Modeling, with a 95% Confidence Interval.

**Results:**

among the sexuality dimensions, only physical and social adversities exerted statistically significant effects on depressive symptoms (SC=-0.095; p=0.003), but with low magnitude. Moreover, all sexuality dimensions had statistically significant effects on quality of life, being of low magnitude for sexual act (SC=0.171; p=0.010) and for physical and social adversities (SC=0.228; p<0.001), and moderate magnitude for affective relationships (SC=0.474; p<0.001).

**Conclusions:**

effects of different magnitudes were observed between sexuality dimensions on participants’ depressive symptoms and quality of life.

## RESUMO

**Objetivos:**

analisar os efeitos da sexualidade na sintomatologia depressiva e qualidade de vida de pessoas idosas.

**Métodos:**

estudo transversal e analítico, desenvolvido com 596 pessoas idosas, que preencheram quatro instrumentos para a coleta dos dados. Os dados foram analisados com o Teste de Kruskal-Wallis e a Modelagem de Equações Estruturais, com Intervalo de Confiança de 95%.

**Resultados:**

dentre as dimensões da sexualidade, somente as adversidades física e social exerceram efeitos estatisticamente significantes na sintomatologia depressiva (CP=-0.095; p=0,003), porém com fraca magnitude. Além disso, todas as dimensões da sexualidade exerceram efeitos estatisticamente significantes na qualidade de vida, sendo de fraca magnitude para o ato sexual (CP=0.171; p=0,010) e para as adversidades física e social (CP=0.228; p<0,001), e moderada magnitude para as relações afetivas (CP=0.474; p<0,001).

**Conclusões:**

observaram-se efeitos de diferentes magnitudes entre as dimensões da sexualidade sob a sintomatologia depressiva e qualidade de vida dos participantes.

## RESUMEN

**Objetivos:**

analizar los efectos de la sexualidad sobre los síntomas depresivos y la calidad de vida en ancianos.

**Métodos:**

estudio transversal y analítico, desarrollado con 596 ancianos, que cumplimentaron cuatro instrumentos para la recolección de datos. Los datos se analizaron mediante la prueba de Kruskal-Wallis y el modelado de ecuaciones estructurales, con un Intervalo de Confianza del 95%.

**Resultados:**

entre las dimensiones de la sexualidad, solo las adversidades físicas y sociales ejercieron efectos estadísticamente significativos sobre los síntomas depresivos (CE=-0,095; p=0,003), pero de baja magnitud. Además, todas las dimensiones de la sexualidad tuvieron efectos estadísticamente significativos sobre la calidad de vida, siendo de baja magnitud para el acto sexual (CE=0,171; p=0,010) y para las adversidades físicas y sociales (CE=0,228; p<0,001), y magnitud moderada para las relaciones afectivas (CE=0,474; p<0,001).

**Conclusiones:**

se observaron efectos de diferente magnitud entre las dimensiones de la sexualidad sobre los síntomas depresivos y la calidad de vida de los participantes.

## INTRODUCTION

As older adults increase, there is also a substantial increase in diseases prevalent in old age, such as neurological-degenerative diseases and depression^(1)^. Depression is directly related to increased morbidity and mortality, low therapeutic compliance and self-care deficit. Moreover, the disease substantially burdens health services and decreases the quality of life (QoL) of those affected, constituting an important public health problem, due to its undesirable outcomes in the individual and family contexts^(2)^.

The rate of diagnosis of depression in older adults is undersized. There are estimates that 50% of cases are not diagnosed by primary health care professionals, especially because symptoms are similar to senescence. Some symptoms mentioned are related to physical complaints, such as drowsiness, lack of appetite, fatigue and indisposition, which are often confused with the organic process of adaptation to aging^(1)^.

Depression is a psychiatric condition characterized by reduced mood, whose etiology is especially related to the situation of loss and physiological and social role changes, commonly observed in old age. This is one of the most serious conditions, since 48.9% of older adults suffer from chronic diseases, and depression accounts for 9.2% of this estimate^(1)^.

From this perspective, it is essential to implement new care strategies to significantly reduce cases of depression among older adults. The role of Primary Health Care (PHC) in activities that promote active and successful aging becomes evident, especially addressing issues about sexuality among older adults in the most diverse forms of individual and collective interaction, because it is pointed out that sexuality is part of their identity, social relationships and mental health^(3)^, in addition to affirming the desire to express it^(4,5)^.

Sexuality is a concept not reduced to sex, and can be understood as a construct characterized by multidimensionality, involving expressions of feelings, cognition and thoughts such as affection, caress, touch, embrace, intimacy, love and the sexual act itself^(6,7,8,9)^. It is, therefore, a natural experience that obeys an individual’s own physiological impulses^(10)^.

The great challenge, however, in working on the sexuality of older adults, concerns the existence of judgments and constant surveillance by society, because it is erroneously disclosed that sexuality can only be experienced by young people, which ends up inhibiting older adults’ identity, and their experiences become suppressed. As a consequence, the individual may have implications for mental health by not feeling socially able to have such an experience^(11,12)^.

However, it is reported that the expression of sexuality begins from birth^(13)^ and continues to develop in old age^(14)^. It is a component considered a basic human need, essential for health^(15)^, well-being^(13)^ and QoL^(10,16)^. QoL is a term used to refer to individual perception in relation to the harmony existing between several aspects that structure a person’s daily life, such as intrinsic and extrinsic factors that relate to lifestyle^(17)^. Due to the need to increase quality in the aging process, QoL has become an important health marker, capable of reorienting care practices^(18)^.

The World Health Organization (WHO) defines QoL as “an individual’s perception of their position in life in the context of the culture and value systems in which they live and in relation to their goals, expectations, standards and concerns”^(19)^.

Given the above, the hypothesis of this study is that sexuality exerts strong and negative effects under depressive symptoms, and strong and positive effects under older adults’ QoL. If this hypothesis is confirmed, sexuality may be one of the strategies capable of assisting in the management of QoL and cases of depression among older adults, thus emerging a new perspective of action of health professionals, especially in primary care.

## OBJECTIVES

To analyze the effects of sexuality on depressive symptomatology and QoL of older adults.

## METHODS

### Ethical aspects

Considering the ethical aspects of scientific research, this study was submitted to the Research Ethics Committee of the *Escola de Enfermagem de Ribeirão Preto* at *Universidade de São Paulo* (EERP/USP), obtaining approval in 2020, in accordance with Resolution 466/2012 of the Brazilian National Health Council (CNS). It is noteworthy that all participants read and agreed with the Informed Consent Form (ICF), made available in full on the questionnaires’ initial page. A copy of the ICF was sent in hidden mode to all informed emails, thus ensuring personal information anonymity.

### Study design, period and site

This is a cross-sectional study, developed between July and October 2020. The study was developed exclusively online with participants from the five Brazilian regions: North, Northeast, Midwest, Southeast and South. There were no face-to-face meetings between researcher and participants.

### Sample; inclusion and exclusion criteria

The sample size was defined, *a priori*, considering an infinite population, conservative proportion of 50%, confidence level of 95% (zα/2 = 1.96) and margin of error of 5% (α=0.05), which resulted in a sample of 385 participants. However, in order to remedy losses due to insufficient responses to the questionnaire, there was an increase of more than 50% (n=211), which resulted in a final sample size of 596 participants.

Participants were recruited according to consecutive non-probabilistic sampling. We included participants of both sexes, aged 60 years or over, married, in a stable relationship or with a steady partner, because the instrument on sexuality assesses constructs referring to the participant and their respective spouse^(20)^, residing in any region of Brazil, with internet access and active account on Facebook.

We excluded older adults with functional dependence, hospitalized, residents in long-stay institutions or similar, and those living with some neurodegenerative pathology that made it impossible to understand the instruments. This control was performed through four questions, as a form of screening, made available at the beginning of the page, in which the initial information of the study was provided. Only the older adults who denied all the questions asked participated in the study.

### Study protocol

Data collection was carried out exclusively on Facebook, without face-to-face meetings. The invitation to participate was published on the Facebook page through a hyperlink that provided direct access to the study questionnaire. The questionnaire was built on Google Forms and is divided into four sections.

The first section was elaborated in order to trace the participants’ biosociodemographic profile, with information such as age group, sex, marital status, religion, ethnicity, education, sexual orientation, orientation on sexuality by health professionals and geographic location.

The second section referred to collection of data on sexuality, which was assessed by the Elderly Affective and Sexual Experiences Scale (EVASI), built and validated in Brazil in 2012^(20)^. It is a psychometric scale with 38 items and three dimensions: sexual act, affective relationships and physical and social adversity. EVASI responses are arranged in a Likert-type scale, ranging from 1 (never) to 5 points (always), and there is no cut-off point. The results are interpreted from the perspective that the highest score indicates a better experience of sexuality by older adults^(20)^. It is noteworthy that the physical and social adversity dimension consists of negative issues and, therefore, there was an inversion of values so that all dimensions were in the same direction of analysis.

The third section referred to collection of information on depression screening through the Geriatric Depression Scale (GDS), validated in Brazil and composed of 15 questions^(21)^, whose score ranges from 0 to 15 points. Values of 5/6 (not case/case), which can be interpreted as follows, no symptoms (0 to 5 points), mild depressive symptoms (6 to 10 points) and severe depressive symptoms (11 to 15 points), were considered as cut-off points^(22)^.

Finally, the fourth section was designed to assess participants’ QoL, using a standardized instrument validated in Brazil called World Health Organization Quality of Life – Old (WHOQOL-Old)^(23)^. It is a specific instrument for older adults, organized into 24 questions and six facets: sensory skills; autonomy; past, present and future activities; social participation; death and dying; and intimacy. The answers are structured in a Likert-type scale (1 to 5), and the total score varies between 24 and 100 points. This instrument does not have a cut-off point, and is interpreted as follows: the higher the score, the better the QoL and, conversely, the lower score indicates worse QoL.

In this study, a generic version of the QoL instrument was not used, given that, initially, participants showed resistance in joining the study, due to the excess of questions. Thus, it was decided to remove the WHOQOL-Bref instrument from the study, remaining only the WHOQOL-Old. The decision to continue the WHOQOL-Old was based on the EVASI validation study^(20)^, in which the author found a strong correlation between these two instruments (0.64), revealing that the EVASI predicts 41% of the QoL assessed by the WHOQOL-Old. Furthermore, the author found narrow confidence limits, with a sample slope between 0.24 and 0.33, F(1.198)=11.74 and p-value<0.001, considering an interval of 95%, which shows that the results were not due to sampling error^(20)^.

It is worth noting that, due to the fact that older adults actively participate in social networks and have sufficient skills to handle electronic devices to access networks (laptop, computer, tablet and/or cell phone), a questionnaire to assess cognition was waived. Additionally, before participants had access to the questionnaire questions, they were required to include the email, in order to avoid multiple responses from the same participant.

### Analysis of results, and statistics

Initially, data were transported to the Statistical Package for the Social Sciences (SPSS), version 25.0. Data distribution was analyzed using the Kolmogorov-Smirnov test, which showed non-normal distributions (p<0.001). Nominal variables were presented through absolute and relative frequencies, while numerical variables were presented through median and interquartile range (IQR).

Non-parametric statistics, represented by the Kruskal-Wallis H test, were used to compare participants’ sexuality and QoL according to depressive symptom intensity. To analyze the effects of sexuality (independent variable) on depressive symptomatology and QoL (dependent variables), Structural Equation Modeling (SEM) was performed in the STATA statistical software. It is a method of analysis that allows investigating the plausibility of theoretical models capable of explaining relationships between several variables^(24)^. In addition, although the present study is cross-sectional, the application of SEM allows the measurement of direct and indirect effects of one variable on the other^(25)^.

In the proposed model, one latent variable with factor load indicators above 0.50 and four observed variables were included. Thus, the latent QoL was formed by the domains autonomy (DOM2), past, present and future activity (DOM3), social participation (DOM4) and intimacy (DOM6). Meanwhile, the observable variables were formed by the domains sexual act (EVASI1), effective relationships (EVASI2) and physical and social adversity (EVASI3), and by depressive symptomatology (GDS). The standardized coefficients (SC), along with their respective 95% Confidence Intervals (95% CI) were used for data interpretation, as recommended by Kline^(26)^: small effect (SC=0.10); medium effect (SC=0.30) and strong effect (SC>0.50).

To certify the adequacy of the model, some adjustment indexes were used: the Comparative Fit Index (CFI) and the Tucker–Lewis Index (TLI), with values closer to 1, indicating a better fit^(25)^; the Adjusted Goodness-of-Fit Index (AGFI) absolute fit index, ranging from 0 to 1, with values ≥ 0.90 indicate well-fitted models^(27)^; a Standardized Root Mean Square Residual (SRMR), with values < 0.08, indicating a good fit, and < 0.10, an acceptable fit^(24,26)^; and the Root-Mean-Square Error of Approximation (RMSEA), with its 90% CI (90% CI), and the following interpretation: perfect fit (RMSEA=0); good fit (RMSEA <0.05); moderate fit (RMSEA=0.05–0.08); mediocre fit (RMSEA=0.08–0.10); and inadequate fit (RMSEA>0.10)^(28)^.

## RESULTS

Table 1 shows the description of participants’ biosociode-mographic variables. There is a greater predominance of older men (66.1%), white (65.3%) with high school education (36.9%) and who have never received guidance on sexuality by health professionals (77.0%). Moreover, there was a higher prevalence of older adults without depressive symptoms (72.0%), followed by mild (19.1%) and severe (8.9%) symptoms.

**Table 1:** Participants’ biosociodemographic characteristics, Ribeirão Preto, São Paulo, Brazil, 2020

Variáveis	n	%
Sex		
Male	394	66.1
Female	202	33.9
Age (years)		
60 - 64	295	49.5
65 - 69	188	31.5
70 - 74	80	13.4
75 - 79	28	4.7
≥ 80 years	5	0.8
Education		
Primary	51	8.6
Elementary school	107	18.0
High school	220	36.9
Higher education	218	36.6
Ethnicity		
White	389	65.3
Yellow	11	1.8
Black	30	5.0
Brown	151	25.3
Indigenous	5	0.8
Does not know	10	1.7
Religion		
Catholicism	291	48.8
Protestantism	85	14.3
Spiritism	73	12.2
African religion	11	1.8
Other	66	11.1
Without religion	70	11.7
Marital status		
Married	358	60.1
Stable union	120	20.1
With fixed partnership	118	19.8
Time living together		
≤5 years	116	19.5
Between 6 and 10 years	46	7.7
Between 11 and 15 years	60	10.1
Between 16 and 20 years	35	5.9
> 20 years	339	56.9
Living with children		
Yes	188	31.5
No	382	64.1
No children	26	4.4
Received sexual orientation		
Yes	137	23.0
Never	459	77.0
Sexual orientation		
Heterosexual	515	86.4
Homosexual	19	3.2
Bisexual	8	1.3
Others	54	9.1
Brazil region		
Northeast	131	22.0
Midwest	78	13.1
Southeast	264	44.3
South	123	20.6

Table 2 shows that older adults without depressive symptoms better experience their sexuality when compared to those with mild and severe symptoms. It is also observed that, for older adults without depressive symptoms, the sexual act and affective relationships presented similar scores, indicating that the experiences in these dimensions are equivalent. Otherwise, older adults with severe depressive symptoms had better experience in sexuality in the sexual act to the detriment of affective relationships.

**Table 2:** Comparison of sexuality and quality of life of older adults according to depressive symptoms, Ribeirão Preto, São Paulo, Brazil, 2020

Variables	Depressive Symptomatology			H	p value
	Absent Median (IQR)	Mild Median (IQR)	Severe Median (IQR)		
Sexuality					
SA	76.00 (67.00 – 81.50)	62.00 (54.25 – 72.25)	58.00 (44.00 – 65.00)	112.863	**<0.001** *
AR	76.00 (67.00 – 82.00)	63.00 (55.00 – 71.25)	56.00 (42.50 – 66.00)	115.808	**<0.001** *
PSA	9.00 (8.50 – 11.00)	8.00 (7.00 – 11.00)	7.00 (5.00 – 9.00)	53.745	**<0.001** *
GS	159.00 (142.50 – 169.00)	134.50 (114.00 – 150.00)	122.00 (95.50 – 136.50)	111.817	**<0.001** *
Quality of life					
SS	81.25 (68.75-93.75)	75.00 (56.25-87.50)	68.75 (43.75-81.25)	32.570	**<0.001** *
AUT	75.00 (62.50-81.25)	62.50 (50.00-68.75)	50.00 (34.37-62.50)	111.409	**<0.001** *
PPFA	75.00 (62.50-81.25)	50.00 (37.50-62.50)	31.25 (25.00-43.75)	173.166	**<0.001** *
SP	75.00 (56.25-81.25)	50.00 (37.50-62.50)	37.50 (21.87-50.00)	155.368	**<0.001** *
DD	75.00 (50.00-87.50)	56.25 (31.25-75.00)	50.00 (25.00-68.75)	44.550	**<0.001** *
INT	75.00 (68.75-87.50)	56.25 (37.50-75.00)	37.50 (25.00-46.87)	168.229	**<0.001** *
GQoL	71.87 (64.06-80.20)	56.25 (50.00-62.50)	44.79 (38.02-51.56)	220.436	**<0.001** *

* *Statistical significance by the Kruskal-Wallis H test (p<0.05); IQR – interquartile range; SA - sexual act; AR - affective relationships; PSA - physical and social adversities; SS - sensory skills; AUT - autonomy; PPFA - past, present and future activities; SP - social participation; DD - death and dying; INT - intimacy; GQoL - general quality of life.*

Regarding QoL, it is observed that, regardless of the presence or absence of depressive symptoms, the best QoL was evidenced in the sensory abilities facet. Finally, in general, it was observed that older adults without depressive symptoms had better experience in sexuality and better QoL in all dimensions assessed, with statistically significant differences.

**Figure 1:**
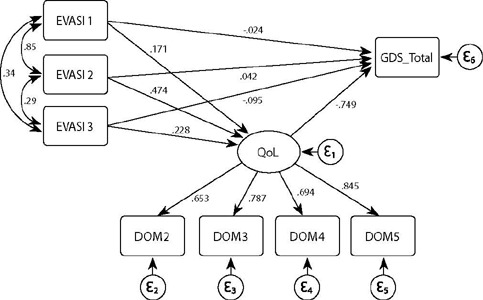
Structural equation model for sexuality (EVASI1, EVASI2, EVASI3), quality of life and depressive symptomatology of older adults, Ribeirão Preto, São Paulo, Brazil, 2020

In the measurement model, the latent QoL showed adequate factor loadings (>0.5) for all domains, except for sensory abilities (DOM1) and death and dying (DOM5). The latent QoL, together with sexuality and depressive symptomatology assessment, composed the proposed structural model (Figure 1). It was possible to show a good fit of the model by assessing the RMSEA (0.09 [95%CI 0.07-0.11]), TLI (0.930), CFI (0.964) and SRMR (0.03) indexes.

It is noted that between the domains of sexuality and depressive symptoms, the direct effects were weak and not significant for EVASI1 (SC=0.024; 95%CI= - 0.119 – 0.072; p=0.684) and EVASI2 (SC=0.042; 95%CI = -0.062 – 0.146; p=0.505), being significant only for EVASI3 (SC=-0.095; 95%CI=-0.147 – -0.043; p=0.003), but with low magnitude, according to Table 3.

**Table 3:** Standardized coefficients of structural equation modeling between sexuality, depressive symptoms and quality of life

	SC	95%CI	p
Measurement model			
DOM 2 ← QoL	0.653	0.604 – 0.703	**<0.001**
DOM 3 ← QoL	0.787	0.750 – 0.824	**<0.001**
DOM 4 ← QoL	0.694	0.646 – 0.742	**<0.001**
DOM 6 ← QoL	0.845	0.816 – 0.874	**<0.001**
Structural model			
Direct effects			
EVASI1 → GDS	-0.024	-0.119 – 0.072	0.684
EVASI2 → GDS	0.042	-0.062 – 0.146	0.505
EVASI3 → GDS	-0.095	-0.147 – -0.043	**0.003**
EVASI1 → QoL	0.171	0.061 – 0.280	**0.010**
EVASI2 → QoL	0.474	0.366 – 0.583	**<0.001**
EVASI3 → QoL	0.228	0.170 – 0.286	**<0.001**
QoL → GDS	-0.749	-0.826 – -0.672	**<0.001**
Indirect effects			
EVASI1 → QoL → GDS	-0.128	-0.210 – -0.046	**0.011**
EVASI2 → QoL → GDS	-0.355	-0.450 – -0.261	**<0.001**
EVASI3 → QoL → GDS	-0.171	-0.219 – -0.122	**<0.001**

*SC – standardized coefficients; 95%CI - 95% Confidence Interval; EVASI – Elderly Affective and Sexual Experience Scale; QoL – quality of life; GDS - Geriatric Depression Scale.*

With regard to the direct effects on QoL, they were weak and positive for EVASI1 (SC=0.171; 95%CI= 0.061 – 0.280; p=0.010) and EVASI3 (SC=0.228; 95%CI=0.170 – 0.286; p< 0.001), and moderate for EVASI2 (SC=0.474; 95%CI=0.366 – 0.583; p<0.001).

When considering the indirect effects of sexuality (i.e., mediated by QoL), it is noted that they significantly reduce depressive symptomatology (i.e., mediated by QoL), being of low magnitude for EVASI1 (SC= -0.128; 95%CI= -0.210 – -0.046; p=0.011) and EVASI3 (SC= -0.171; 95%CI -0.219 – -0.122; p<0.001), and moderate magnitude for EVASI2 (SC= -0.355. 95%CI= -0.450 – -0.261; p<0.001), according to Table 3.

## DISCUSSION

Although this study was developed with older adults residing throughout the Brazilian territory, peculiar biosociodemographic characteristics were observed that are not the reality of most Brazilian older population, such as people with high schooling, white people and with access to the internet.

Moreover, the fact that the participants of this study were predominantly male (66.1%) and with high educational level, considering high school and higher education (73.5%), differs from other investigations developed with the same public, in which there is a higher prevalence of female older adults with low education^(1,2)^.

Regarding the prevalence of depressive symptoms, the results revealed here are similar to other studies^(1,2)^, with prevalence of older adults without depressive symptoms assessed with the same psychometric instrument. This is an exultant result, given that, in the present investigation, older adults without depressive symptoms had better experiences in sexuality and better QoL in all dimensions assessed, when compared to those with that condition, as shown in Table 2. *EVASI – Elderly Affective and Sexual Experience Scale; QoL – quality of life; GDS - Geriatric Depression Scale.*

In fact, the literature shows that depression is responsible for increased morbidity and mortality and a significant reduction in the QoL of those affected, also contributing to the worsening of existing pathologies, further increasing the risk of death and morbidity^(1)^. Moreover, according to the results of a study^(29)^ developed with older adults, the absence of depressive symptoms was associated with greater knowledge about sexuality in aging, which may indicate the existence of an inverse relationship between these two constructs.

Another relevant finding was that, in the present study, older adults without depressive symptoms had similar scores in sexual and affective experiences, indicating equivalence in these two dimensions, contrary to other studies^(20,30)^, in which they reveal that affective relationships assume a prominent role in the relationship, while the sexual act takes a secondary position.

However, this evidence does not corroborate the erroneous thought that older adults are asexual and/or do not have sexual desires, since the literature^(8,16)^ states that sexual desires remain in old age and that older adults continue to engage in sexual activities, even if with reduced frequency.

Our results also corroborate this evidence, revealing that experiences in sexuality exerted effects on the proposed outcomes, especially on QoL, in which all sexuality dimensions exerted effects of increasing participants’ QoL.

In relation to depressive symptomatology, it suffered a weak effect only from the physical and social adversities of sexuality dimension. It is a dimension that assesses whether older adults perceive their health as an obstacle to sexual experiences, whether there is discomfort with the changes resulting from aging and whether they are afraid of being victims of prejudice, due to decision-making to experience their sexuality^(20)^.

The study that is closest to the results found here was developed with adult women using a case-control methodology, in which an inversely proportional and statistically significant correlation was observed between participants’ depressive symptoms and sexuality^(31)^. However, as the investigated public differs from our sample, it is not possible to make a rigid comparison with our results, although the literature lacks studies that investigate the relationship between these constructs.

On the other hand, it was observed that older adults with severe depressive symptoms had better experience in sexuality in the sexual act to the detriment of affective relationships, as shown in Table 2. This result may be explained, in part, due to the greater severity of the depressive symptoms present that may be interfering with greater impact on aspects of mood and feelings, making the sexual act dimension more evident.

Although society is currently more adapted to changes in paradigms related to sexuality, it is noticeable that a portion of society still finds it difficult to assess it as something healthy and part of human existence. The fact is that talking about sexuality still causes discomfort and modesty, especially among older adults who show avoidant behavior to discuss their experiences^(29)^. This evidence corroborates the results of the present study, in which 77.0% of participants never received guidance on sexuality from health professionals. Another study^(32)^, developed with older women, identified the same problem: the fear of talking about sexuality, often due to the restrictive education to which they were subjected.

Therefore, it is important that there is greater coverage and qualification of health consultations to older adults^(1)^. It is noteworthy that PHC is a guiding strategy of the care process, contributing significantly to promoting active aging and autonomy for older adults^(2)^, and sexuality is an intrinsic component to the human being to be enjoyed freely and without prejudice or obstacles.

### Study limitations

Participants’ biosociodemographic characteristics are one of the limitations, as they do not represent the majority of Brazilian older adults. Furthermore, it is mentioned that the sample may have been considerably limited, given that data collection occurred in only a single social network and with older adults with internet access, which requires caution in comparing our results with other studies.

### Contributions to nursing

This study contributes significantly to nursing practice. The originality of the theme is noted, being the first investigation that tries to understand the effects of experiences in sexuality in the face of older adults’ depressive symptoms and QoL. Thus, it is encouraged that more nurses, especially those who work in PHC, develop studies with more robust methodologies so that they can somehow elucidate these effects and identify possible causality between the variables.

*In loco* studies can also be articulated with specialists in the subject for the development of standardized and statistically reliable instruments, feasible for application in PHC, without compromising the flow of care. Considering the wide field of work of nurses and the growing commitment to scientific production, they become an important professional in creating strategies to promote sexual health and sexuality for older adults, especially in PHC, considered the gateway to health services and the communication center for the entire health network.

## CONCLUSIONS

It was found that sexuality dimensions exerted effects of different magnitudes on participants’ depressive symptoms and QoL, but without causal relationships, given that the present study is cross-sectional. In this sense, the present study can be considered an initial milestone for health professionals to recognize the benefits of sexuality in old age and start working with it more frequently in health services.

## SUPPLEMENTARY MATERIAL

https://doi.org/10.48331/scielodata.QJT3ED.

0034-7167-reben-76-01-e20210645-sup01
