# Supplementary material for: Sexuality and its effects on older adults’ depressive symptoms and quality of life
Source: Rev Bras Enferm. 2023 Feb 6;76(1):e20210645. doi: 10.1590/0034-7167-2021-0645 (PMC9901350; doi:10.1590/0034-7167-2021-0645)
Supplement: 0034-7167-reben-76-01-e20210645-sup01 [file 0034-7167-reben-76-01-e20210645-sup01.pdf]

Sexo	Idade	Religião	Etnia	Escolaridac	Estado_civ	Tempo_de	Já_teve_or	Orientaçãc	Região
1	1	5	3	5	1	5	1	1	2
1	1	5	3	5	1	5	1	1	2
2	1	6	4	3	1	5	1	1	4
2	1	1	1	5	1	5	1	1	4
1	2	1	1	5	3	1	2	1	4
1	1	2	1	5	1	5	2	1	4
2	1	1	1	4	1	5	1	1	5
1	1	2	3	5	1	5	1	1	3
2	4	3	1	4	1	2	1	1	5
2	1	4	3	3	1	5	1	1	2
1	2	6	1	5	1	5	2	1	4
2	1	1	1	5	1	5	2	1	4
2	3	3	4	5	1	5	1	1	4
1	1	1	4	4	3	5	1	1	2
1	1	5	4	4	1	4	1	1	2
1	2	6	1	5	3	1	2	1	3
2	1	6	1	4	1	5	1	1	4
1	1	1	1	4	3	1	1	1	2
2	1	1	1	4	1	5	1	1	2
2	2	3	4	5	2	3	1	1	2
1	1	1	1	4	1	5	2	1	5
1	2	5	1	2	1	5	1	2	4
2	1	1	4	5	2	5	1	1	2
2	3	2	1	3	3	1	1	1	2
2	3	2	1	3	2	1	1	1	2
2	2	6	1	4	3	1	2	2	4
2	2	1	1	5	1	5	2	1	4
1	2	5	1	4	1	5	1	1	4
1	1	1	1	5	1	5	1	1	4
2	2	2	4	5	2	3	1	1	2
2	2	2	4	5	2	3	1	1	2
1	2	1	1	4	1	5	1	1	5
2	2	1	1	5	1	5	1	1	4
1	1	1	1	5	3	4	2	1	5
2	2	6	4	4	2	1	1	1	2
1	1	1	1	4	3	2	1	1	4
1	1	1	1	1	1	3	1	1	2
2	2	1	1	1	1	5	2	1	5
1	1	1	4	5	1	5	1	1	3
2	2	2	1	4	3	1	1	1	2
1	1	1	1	5	1	5	1	1	4
1	1	3	1	5	1	5	2	1	4
2	3	1	1	4	3	5	1	1	4
2	1	1	1	5	1	5	1	1	2
2	2	4	4	2	1	5	1	1	4
1	1	1	1	5	1	5	1	1	3

2	1	1	1	4	3	2	1	1	5
2	2	1	1	4	2	2	1	2	4
2	1	3	3	4	3	2	1	1	2
2	2	1	4	4	3	2	1	1	5
1	1	6	1	4	2	5	1	1	4
1	3	2	1	3	1	1	1	1	3
1	4	1	1	2	3	1	1	1	4
1	2	2	1	5	3	1	1	1	4
1	2	6	1	5	1	5	1	1	2
2	1	6	3	5	1	5	1	1	3
1	1	1	3	2	3	5	1	1	5
2	1	1	5	4	1	5	1	1	2
2	1	1	4	1	1	5	1	4	3
2	1	1	4	1	1	5	1	4	3
2	2	1	4	3	3	4	1	4	2
2	2	1	4	3	3	4	1	4	2
2	2	1	1	4	1	5	1	1	4
1	2	3	1	4	3	5	2	1	3
1	1	1	1	1	1	5	2	1	5
1	2	6	1	5	1	5	1	1	2
2	2	1	4	1	1	5	1	1	3
2	1	2	1	4	1	5	1	1	5
2	3	1	1	4	1	5	1	1	3
1	1	1	4	4	2	3	2	1	2
2	1	5	1	4	1	5	1	1	5
1	2	6	4	3	1	5	1	1	2
2	1	1	1	4	2	5	1	1	5
2	3	5	1	4	3	2	1	2	5
1	2	3	1	5	2	1	2	1	4
1	2	1	1	5	3	1	1	1	5
1	1	5	4	3	1	4	1	1	2
1	2	1	4	5	2	3	1	4	2
2	2	5	1	3	1	5	1	4	5
2	2	2	1	4	1	4	1	1	4
1	2	5	4	4	1	5	1	1	3
2	1	2	4	4	2	1	1	1	2
2	3	2	1	5	3	1	2	1	5
2	1	1	4	5	1	5	1	1	3
1	1	1	1	5	1	5	1	1	4
2	4	3	1	3	1	5	2	1	4
2	2	6	4	4	2	2	1	1	2
1	1	6	1	5	3	3	2	1	5
1	2	3	3	1	3	1	1	1	3
2	1	2	1	4	2	3	1	1	4
2	1	1	4	1	1	5	1	1	3
2	2	6	1	1	3	1	1	1	5
1	2	5	1	4	3	1	1	1	4

1	1	2	1	4	3	1	1	1	2
1	2	3	1	4	3	3	1	1	5
2	4	1	5	4	2	1	1	1	5
1	1	1	1	5	1	5	2	1	5
1	1	1	4	4	1	5	2	1	2
1	2	2	1	4	3	1	1	1	3
1	2	1	5	4	1	5	2	1	4
1	1	1	1	4	1	5	1	1	4
2	4	1	1	5	1	5	1	1	4
2	1	1	1	5	1	5	1	1	3
2	2	6	1	5	1	5	2	1	5
2	1	1	1	1	1	5	1	3	5
1	2	2	4	2	2	3	1	1	5
1	1	1	1	5	3	1	1	1	5
1	1	2	4	4	3	1	1	1	3
1	2	5	4	4	3	1	1	1	2
2	2	1	1	2	1	5	1	4	4
2	4	1	1	2	1	5	1	1	5
1	1	1	4	5	1	5	2	1	4
1	3	1	4	1	3	5	1	4	2
2	1	1	1	4	1	5	1	1	2
1	1	5	1	3	1	5	1	1	4
1	2	3	1	4	2	5	1	1	5
2	3	1	4	5	2	3	2	1	4
1	1	6	4	3	1	5	1	1	4
1	1	1	1	5	2	2	1	1	5
2	1	2	4	4	1	3	1	1	3
2	2	1	4	5	1	4	1	1	4
2	2	2	1	4	1	5	2	1	4
2	3	6	6	1	1	5	1	4	3
2	1	1	1	4	1	1	2	2	4
2	3	3	1	5	1	5	2	1	5
2	1	5	1	5	1	3	2	1	5
2	2	1	4	1	1	5	1	1	2
2	1	1	1	4	3	2	1	1	2
2	1	2	1	3	1	5	2	1	2
2	2	2	4	1	1	5	1	4	5
2	3	1	1	1	2	4	1	1	5
2	2	1	1	4	1	1	1	1	5
2	1	5	4	4	1	5	1	1	4
2	1	1	1	4	1	5	2	1	3
2	2	1	4	4	1	5	1	4	4
2	1	1	1	2	1	5	1	1	5
1	1	1	1	4	2	5	1	1	2
2	1	6	1	2	1	5	1	1	4
1	1	6	4	1	2	1	2	4	2
2	1	2	1	1	1	5	1	1	5

2	1	6	1	5	1	5	1	1	4
2	1	6	1	4	1	5	1	1	4
1	1	1	1	4	3	1	1	1	4
2	3	1	1	3	1	5	1	1	5
2	1	1	1	4	1	5	1	1	4
2	4	5	1	4	2	5	1	1	4
2	4	5	1	5	3	2	1	1	4
2	3	1	1	1	1	5	1	1	5
2	3	5	1	5	1	5	2	1	4
2	1	1	1	3	2	4	2	1	4
1	1	1	1	4	2	3	1	1	4
2	3	1	1	5	1	5	1	1	4
2	1	1	3	5	1	5	1	2	2
1	1	3	4	5	3	3	2	4	2
2	3	1	1	4	2	5	1	4	5
1	3	3	1	2	1	5	1	1	4
1	2	2	1	2	1	1	1	1	2
2	2	5	1	5	1	5	1	1	4
1	2	6	4	4	2	5	2	1	4
2	2	6	1	4	3	2	1	1	2
2	2	2	1	5	2	4	1	1	4
2	1	3	1	4	1	5	2	1	4
1	2	1	4	5	3	2	2	1	3
1	1	1	4	5	3	5	2	1	2
2	2	2	1	4	1	5	1	1	4
2	2	1	4	5	1	5	1	1	2
2	1	1	4	4	2	1	1	1	4
2	4	3	1	2	1	5	2	1	5
2	1	5	1	5	1	3	1	1	4
1	2	3	1	4	3	5	2	1	4
2	1	5	4	1	1	2	1	1	5
2	4	1	1	3	1	5	1	1	3
2	1	2	4	5	2	5	1	1	2
1	2	3	1	5	3	1	2	1	4
1	3	1	1	4	1	5	1	4	4
1	1	6	6	4	1	5	2	1	4
1	2	3	1	2	2	1	2	4	3
2	2	2	4	4	1	5	1	1	3
2	2	6	1	4	2	1	1	1	5
1	1	3	1	4	3	1	1	1	5
1	3	1	4	3	1	5	1	1	3
2	1	6	6	5	2	3	2	1	2
2	1	1	4	4	3	1	1	1	2
2	2	6	4	2	2	1	1	1	4
1	3	2	1	4	2	3	1	1	4
1	1	2	1	4	2	1	1	1	5
2	3	1	1	5	1	5	1	1	4

1	1	3	1	5	3	4	2	1	5
2	3	6	1	5	2	5	1	4	4
1	1	3	4	4	1	2	2	1	4
1	1	3	1	4	3	1	1	1	4
1	3	1	4	1	1	5	1	1	4
2	3	1	1	3	2	5	1	1	4
1	1	1	4	1	3	1	1	1	2
1	1	1	1	4	1	5	1	1	5
1	1	2	2	3	1	1	1	1	3
1	1	2	1	1	3	1	1	1	5
1	2	6	1	4	1	5	1	1	4
1	1	6	4	5	2	2	1	1	4
2	1	1	1	4	2	4	2	1	2
2	2	2	1	5	1	5	1	1	5
2	4	1	1	4	3	3	2	1	4
1	2	5	1	3	2	1	1	1	2
1	1	3	4	5	1	5	1	1	2
2	1	4	4	4	3	1	1	1	2
1	1	1	1	4	3	5	1	1	5
1	2	5	3	4	3	2	1	1	4
1	1	2	4	4	3	1	1	1	4
1	2	1	1	5	3	1	1	1	4
2	1	6	4	4	3	2	1	3	5
2	2	1	1	5	1	5	1	1	4
2	2	1	4	5	1	3	1	1	2
1	2	4	1	5	2	5	1	1	4
2	2	3	1	2	2	3	1	1	4
2	1	1	1	4	1	5	1	1	5
1	2	3	1	5	2	5	1	2	4
1	3	1	4	5	1	5	1	1	4
2	2	1	1	5	3	1	1	1	4
1	1	1	4	5	2	5	2	1	4
2	3	5	1	5	2	3	1	1	4
1	2	6	4	5	1	5	2	1	5
1	2	3	3	5	3	1	2	1	4
1	1	3	1	5	1	5	2	1	4
1	3	1	1	5	3	4	1	1	4
1	2	5	4	5	3	5	1	1	3
2	5	6	1	5	1	5	2	1	4
1	1	3	1	5	3	1	1	1	2
1	2	3	4	5	1	5	1	1	4
2	2	6	4	5	1	5	1	1	4
1	1	6	2	4	3	5	2	4	5
2	1	1	1	5	2	3	2	1	4
2	1	5	1	4	1	5	1	1	5
1	1	1	2	3	1	5	1	1	2
1	1	5	1	1	3	2	1	1	5

1	1	3	1	5	1	5	2	3	5
1	2	2	3	2	1	5	1	1	4
1	2	2	4	2	2	1	1	1	3
1	1	5	1	4	2	5	2	1	4
1	2	1	1	5	3	1	1	1	2
2	1	1	1	5	1	5	1	1	5
1	2	3	1	5	3	1	2	1	5
1	1	3	1	5	3	3	2	4	4
2	2	6	1	5	1	4	1	1	2
1	1	3	4	2	3	1	1	1	2
1	2	1	3	5	2	1	1	1	2
1	2	5	1	3	3	1	2	1	5
1	4	5	1	4	1	5	1	1	5
2	3	2	1	5	1	5	2	1	4
1	1	5	1	5	2	2	2	1	4
1	1	6	1	4	3	3	1	1	4
1	2	3	1	5	3	2	2	1	4
2	2	1	1	5	1	5	1	1	2
1	1	1	4	4	3	1	2	1	2
2	3	5	4	5	1	5	1	1	4
2	3	6	1	5	2	3	1	1	4
2	4	1	1	1	1	5	1	1	4
1	4	1	1	5	2	5	1	1	4
1	3	2	1	5	2	5	1	1	4
1	2	5	1	1	2	5	1	4	4
1	2	1	1	5	1	5	1	1	2
2	1	1	1	5	1	5	1	1	2
2	1	1	4	4	1	5	1	1	3
2	1	1	1	5	1	4	1	1	4
2	1	3	1	5	1	5	2	4	2
1	1	5	1	4	1	5	1	1	3
2	2	1	3	4	2	1	2	3	4
1	4	1	1	4	3	1	1	2	3
2	1	1	1	5	1	5	2	1	4
2	4	3	1	4	3	3	1	2	4
2	3	1	1	5	2	1	1	1	3
2	2	6	1	5	1	5	2	1	5
2	3	1	1	4	1	5	1	1	4
1	4	1	1	2	1	5	2	1	4
2	1	1	1	4	1	5	1	1	4
1	2	1	1	2	1	3	1	1	5
2	2	1	1	5	1	5	1	1	2
1	1	5	3	4	3	4	1	1	4
2	2	1	4	4	2	4	1	3	4
1	2	1	1	2	1	1	1	1	5
1	1	1	1	4	1	3	1	1	5
1	2	6	1	4	1	5	1	1	4

1	1	3	4	5	1	3	2	2	4
2	1	1	1	5	1	5	1	1	4
1	1	3	4	4	3	1	1	1	4
1	1	2	1	2	1	4	1	1	4
1	2	3	1	5	1	5	1	1	5
2	3	1	5	5	1	5	1	1	3
2	1	1	1	5	2	3	1	1	3
2	2	1	1	2	1	5	1	1	4
1	1	5	4	5	3	1	1	1	4
2	1	1	3	4	2	5	1	1	4
2	3	6	1	5	1	5	1	1	4
1	1	1	4	4	3	1	2	1	4
2	2	1	1	5	1	5	1	1	5
2	1	4	1	4	3	3	1	1	4
2	4	6	4	2	1	3	1	1	2
1	1	5	4	4	2	1	1	1	2
2	2	5	1	4	1	5	2	1	4
2	2	2	1	4	1	5	2	1	4
2	3	1	1	1	1	5	1	1	5
2	1	1	1	4	1	5	1	1	4
2	1	2	2	4	1	5	1	4	3
2	1	2	4	2	1	3	1	4	2
2	2	5	1	4	1	5	1	1	5
2	2	1	1	4	1	5	2	1	5
2	3	1	1	5	1	5	2	1	5
2	1	4	4	4	3	1	2	1	4
1	1	3	1	5	1	3	2	1	3
1	1	3	1	5	1	3	2	1	3
2	3	3	1	5	3	1	1	1	5
1	2	1	1	5	3	5	1	1	2
2	2	1	4	2	1	5	1	1	3
2	3	2	1	1	1	5	1	1	4
1	1	5	1	3	1	1	1	4	5
2	2	2	4	4	3	3	1	1	4
2	2	2	1	5	2	5	1	1	3
2	1	1	1	3	1	5	1	1	4
2	2	5	1	5	1	5	1	3	4
2	2	1	1	2	1	5	1	1	5
2	3	2	4	3	1	5	1	1	4
2	2	4	4	5	1	5	1	1	4
2	1	2	1	4	1	5	1	2	4
1	1	2	1	5	1	5	2	1	4
1	2	1	1	5	1	5	1	1	5
1	1	5	1	4	1	1	1	1	3
2	1	1	4	4	1	3	1	4	2
2	4	1	1	2	1	5	1	1	2
1	2	1	1	4	1	5	1	1	4

2	1	2	4	5	1	5	1	1	3
1	2	1	3	5	3	1	1	1	4
1	1	2	4	5	3	1	2	1	3
1	2	1	1	5	1	5	1	1	4
2	1	5	1	4	1	5	1	1	4
2	2	1	4	5	1	3	1	1	2
2	1	3	1	5	2	4	1	1	3
2	1	6	2	2	3	1	1	1	3
1	3	1	1	1	3	1	1	4	5
2	4	1	1	5	2	5	1	1	4
2	3	4	1	4	1	4	1	1	4
1	1	2	1	5	1	4	1	1	4
2	3	2	1	4	1	5	1	1	5
2	2	1	1	4	1	5	1	1	2
1	2	5	1	4	2	5	2	4	5
2	4	1	1	1	1	5	1	4	4
2	1	5	1	2	3	1	2	1	4
2	2	1	1	5	1	5	2	1	4
2	2	5	1	4	3	1	2	1	4
2	1	5	1	4	3	3	2	1	4
2	1	1	1	4	3	1	1	4	4
1	2	1	4	1	3	2	1	1	4
1	1	5	3	2	3	4	1	1	4
2	1	1	1	4	1	5	2	1	5
2	1	1	1	5	3	1	1	1	4
2	1	1	4	3	1	5	1	2	5
2	2	1	4	3	2	1	1	1	3
2	1	2	1	4	2	1	1	1	2
2	1	1	4	4	1	5	1	1	4
2	1	1	1	4	2	2	1	1	4
2	2	2	1	4	1	5	1	1	2
2	2	2	3	5	1	5	1	1	4
2	2	1	1	4	1	5	2	1	4
2	1	5	1	5	1	2	1	1	4
2	1	4	1	5	2	3	1	2	2
2	2	2	1	5	1	2	1	1	3
2	1	3	4	3	2	3	1	1	3
2	1	2	1	4	1	5	2	1	4
2	3	1	1	3	1	5	1	1	5
2	1	3	1	5	1	4	1	1	4
2	2	1	1	4	1	5	2	1	2
2	1	1	1	1	3	5	2	4	4
2	1	6	4	3	1	5	1	1	4
2	1	1	4	2	3	2	1	1	4
2	1	1	4	2	1	5	1	1	5
1	3	1	1	4	2	3	2	1	5
1	1	2	1	5	1	5	1	1	5

2	1	6	1	5	2	1	1	1	5
2	2	2	1	4	1	5	1	1	2
2	3	1	1	2	2	5	2	4	5
2	1	1	1	1	1	4	1	4	4
2	1	6	1	4	1	5	1	1	5
2	3	1	1	1	2	1	1	1	4
2	1	2	1	5	1	5	1	1	4
2	1	1	1	3	1	5	1	1	4
2	2	1	1	3	1	5	1	1	4
2	2	3	1	4	2	4	2	1	4
2	1	1	1	5	3	3	1	1	3
1	2	6	3	4	3	2	1	1	4
2	3	1	4	2	2	4	2	1	2
1	1	1	4	4	2	2	1	1	4
2	3	3	1	5	1	5	1	1	4
2	3	1	1	5	1	5	1	1	4
2	1	2	1	5	1	5	1	1	2
2	2	1	1	5	1	5	1	1	4
1	1	5	1	1	1	5	2	4	5
1	1	1	6	1	3	1	1	4	5
2	1	1	4	3	1	5	1	4	4
2	2	1	5	2	1	5	1	1	2
2	2	1	1	5	1	5	1	1	2
1	1	1	4	5	1	5	2	4	5
2	2	1	1	2	2	1	1	1	4
2	2	1	4	4	1	5	1	1	2
2	1	1	4	4	1	5	1	1	4
1	1	1	1	5	1	5	1	1	4
2	2	5	1	3	1	3	1	4	2
2	2	5	1	3	2	1	1	1	4
2	3	3	1	5	1	5	1	1	5
2	2	1	4	2	1	3	2	1	4
2	1	1	1	5	3	1	1	1	2
2	1	1	4	5	1	5	2	1	4
2	2	1	1	2	2	1	1	1	4
2	3	3	4	5	1	2	1	1	3
2	2	1	1	4	1	5	1	1	4
2	1	1	1	5	2	5	1	1	2
2	1	1	4	4	3	1	2	4	2
2	4	1	1	4	1	5	1	1	4
1	1	3	1	4	2	1	2	1	4
2	3	5	1	2	3	1	1	1	3
2	4	1	1	1	1	5	1	1	3
2	2	3	4	4	2	3	1	4	4
2	1	6	1	4	1	5	1	1	4
1	1	1	1	2	1	5	2	1	4
2	5	1	1	5	1	5	2	1	2

1	1	1	3	2	1	5	1	1	2
2	1	1	4	4	2	1	1	1	2
1	1	1	1	4	1	5	1	1	4
2	1	2	1	2	1	1	1	4	3
2	2	3	4	4	2	1	1	1	3
1	2	3	4	4	1	5	1	1	4
2	1	1	4	5	1	5	1	1	2
2	1	1	4	2	1	5	1	2	4
2	1	1	1	4	1	5	1	1	5
2	2	2	4	5	1	3	1	1	2
2	3	1	1	4	2	5	1	1	5
2	1	3	4	5	3	3	2	1	2
2	2	6	4	4	1	5	1	1	3
2	1	3	4	5	1	5	2	1	3
1	3	5	6	4	1	2	2	4	2
1	1	2	1	1	1	5	1	1	4
2	1	6	4	1	2	5	1	4	3
1	2	1	4	1	1	5	1	1	4
1	1	5	4	5	3	1	1	1	2
1	2	6	1	4	1	5	1	1	4
2	2	1	1	4	1	1	1	1	2
2	1	1	1	2	1	5	2	1	3
2	1	1	1	5	1	5	1	1	4
2	2	1	1	3	2	1	1	1	3
2	3	1	1	5	1	5	1	1	2
2	2	1	4	5	3	5	2	1	4
2	2	1	1	5	2	2	2	1	4
2	1	2	1	4	1	2	1	1	2
1	3	1	1	5	1	5	1	1	5
1	3	1	1	5	1	5	1	1	5
2	3	1	1	2	2	1	1	1	4
1	3	1	1	4	1	5	1	1	4
2	2	2	4	4	1	5	1	1	3
2	1	4	4	4	2	2	1	1	3
1	1	5	1	4	1	1	1	1	3
2	4	1	1	5	1	5	1	1	4
2	2	1	4	4	1	2	1	1	2
2	1	1	4	2	1	5	1	1	3
2	2	1	1	4	1	5	1	1	4
2	1	1	1	5	1	5	2	1	5
2	2	3	1	4	2	3	1	1	3
2	1	1	1	4	1	5	1	1	2
2	1	1	1	1	3	1	1	2	5
2	3	1	1	3	1	5	1	1	4
2	1	2	4	3	1	3	1	1	4
2	3	1	1	5	1	5	1	1	5
2	3	1	1	4	1	5	1	1	3

2	1	1	4	4	1	5	1	1	4
2	1	5	4	1	1	5	1	1	3
1	1	6	1	5	1	5	2	1	4
2	1	1	1	5	1	5	1	1	5
2	2	2	6	5	1	5	1	1	5
2	1	1	4	5	1	5	1	1	4
2	1	2	1	3	1	5	1	1	4
2	1	6	6	4	1	5	1	1	4
2	4	1	1	4	1	5	1	1	5
2	2	1	1	5	1	5	1	1	4
2	1	2	1	5	1	4	1	1	2
1	1	1	3	4	2	2	2	4	5
2	2	1	1	5	1	5	2	1	2
2	1	6	1	4	2	3	1	1	4
2	1	1	4	4	1	5	1	1	4
2	1	1	1	5	1	4	1	1	4
2	3	2	3	4	1	5	1	1	4
2	1	2	4	2	1	4	1	1	2
1	1	5	1	5	2	1	2	1	2
2	3	3	1	5	2	1	1	1	2
2	1	5	1	5	1	1	1	1	5
2	1	1	2	4	2	5	1	1	2
2	1	1	1	4	1	5	2	1	2
2	2	1	4	4	1	5	1	4	3
1	1	5	1	3	1	5	1	4	5
1	3	6	1	5	1	5	1	1	2
2	1	1	3	5	1	4	2	2	4
1	2	6	3	4	1	3	2	1	4
2	1	1	1	4	1	5	2	1	5
2	1	2	1	5	2	4	1	1	2
2	1	1	1	4	1	5	1	1	4
1	2	3	1	5	2	5	2	1	4
1	1	3	1	2	1	5	2	1	4
1	1	1	4	4	1	5	2	1	5
2	1	2	4	4	1	5	2	1	2
2	2	1	1	1	1	5	1	1	4
2	1	1	1	3	1	5	1	1	4
2	1	1	6	3	1	5	1	4	2
2	2	2	2	3	1	5	1	1	4
2	2	6	4	5	2	3	1	4	3
2	1	5	4	2	2	1	1	1	4
2	1	6	3	4	1	3	1	1	2
2	1	1	1	1	1	5	1	1	5
2	3	6	1	5	1	5	1	1	4
1	4	1	1	5	3	2	2	1	4
2	4	3	1	4	1	5	1	4	5
2	1	1	4	5	2	3	1	1	2

2	1	1	1	5	1	5	1	1	2
2	1	1	1	5	2	1	1	1	2
2	4	5	1	4	1	5	1	1	4
2	1	5	1	5	2	2	1	1	4
2	2	6	3	4	1	4	1	1	2
2	1	6	1	4	3	1	2	1	4
1	1	3	1	4	1	5	2	1	5
2	1	5	1	4	1	5	1	1	4
2	5	1	4	5	1	5	1	1	4
1	2	1	1	2	3	2	1	1	5
2	1	5	3	3	1	3	1	1	4
2	1	1	1	1	3	1	1	1	4
2	2	6	1	5	1	1	1	1	4
2	5	3	2	3	2	4	1	1	2
2	1	1	1	4	1	5	1	1	4
2	2	2	3	5	2	2	2	1	2
2	2	1	1	2	1	5	1	1	5
1	1	1	1	2	3	1	1	3	2
2	3	1	6	4	2	5	1	4	3
2	2	1	1	3	1	5	1	1	5
2	1	3	4	5	2	3	1	1	2
2	2	1	1	4	1	5	1	1	4
2	3	6	1	4	3	2	1	1	4
2	1	2	1	1	1	5	1	1	5
2	1	6	1	5	1	5	1	4	2
2	1	1	1	5	1	5	1	1	2
2	1	6	4	3	1	5	1	1	4
2	2	1	4	1	1	5	1	1	4
2	2	6	1	5	2	1	2	1	4
2	2	1	4	4	1	4	1	2	2
2	1	1	1	4	1	5	1	1	5
2	2	3	1	5	2	3	1	1	5
2	1	1	1	4	2	2	1	4	5
2	1	6	1	4	2	1	1	1	5
2	3	4	1	5	1	5	1	1	4
2	2	2	1	4	2	5	1	1	5
2	1	2	1	1	1	5	1	4	3
2	1	1	4	5	1	5	1	1	3
2	2	1	1	2	2	1	1	1	4
2	2	1	1	5	1	5	2	1	2
2	3	3	1	3	1	2	1	1	4
2	1	1	1	4	1	3	1	1	4
2	1	1	1	1	1	5	1	1	3
2	3	1	1	1	1	5	1	1	2
1	1	2	4	5	3	1	1	1	3
2	1	2	1	5	1	5	1	1	4
2	2	3	1	5	1	5	2	3	4

2	2	1	1	4	2	2	1	1	3
2	1	1	1	4	1	5	1	1	4
1	1	2	2	4	3	1	1	1	3
2	1	1	1	5	1	5	2	1	4
2	2	6	1	4	1	5	1	1	4
2	1	2	6	1	1	5	1	1	5
2	2	6	4	5	2	5	2	1	2
2	1	1	1	2	2	1	1	1	4
2	3	3	1	4	1	5	1	2	5
2	1	1	1	5	1	5	1	1	4
2	1	1	1	4	1	5	1	1	4
2	1	1	1	5	1	5	1	1	4
2	2	1	1	4	1	5	1	1	5
1	1	1	1	5	1	5	1	1	3
1	1	2	3	5	3	1	2	1	4
2	1	1	1	4	1	5	1	1	5
1	2	5	4	4	3	5	1	1	4
1	1	3	4	3	3	1	2	1	4
2	3	1	1	2	1	5	2	1	3
1	1	2	4	4	3	1	1	4	3
2	1	1	1	1	2	1	1	1	2
2	2	1	4	4	1	5	1	1	2
2	1	1	2	4	1	5	1	4	5
2	3	6	1	3	1	5	1	1	4
2	1	3	1	4	3	1	2	1	4
2	1	1	2	4	2	5	2	1	2
2	5	1	4	5	1	5	1	1	4
1	3	3	1	4	1	5	1	1	4
2	1	3	1	5	1	2	2	2	4
2	1	1	1	4	1	4	1	1	2
1	1	3	1	5	3	2	2	1	4
2	3	1	1	5	1	5	1	1	5
2	3	1	1	4	1	2	1	1	4

Mora_com	EVASI_1	EVASI_2	EVASI_3	EVASI_4	EVASI_5	EVASI_6	EVASI_7	EVASI_8	EVASI_9
1	5	3	2	4	2	4	4	5	3
1	4	3	2	4	5	5	5	5	5
2	4	5	5	5	4	5	5	5	5
2	3	5	5	5	3	3	4	5	5
3	3	3	3	4	2	4	4	5	5
2	5	5	5	5	5	5	5	5	5
2	4	4	4	4	2	3	4	3	3
1	2	1	3	1	2	1	3	1	2
2	3	5	5	5	4	5	5	5	5
1	5	5	3	3	3	5	5	5	5
2	1	1	2	1	1	2	5	4	1
1	5	3	2	3	2	4	5	5	5
2	3	3	1	3	1	1	4	3	5
1	2	4	4	4	4	5	5	5	4
2	3	3	3	3	3	5	5	3	3
2	5	4	4	4	5	5	5	5	5
2	3	3	4	3	2	2	3	2	3
2	4	5	3	5	4	4	5	4	5
2	1	5	4	4	4	4	5	5	5
1	4	5	3	4	4	5	5	5	5
2	4	5	5	4	4	5	5	5	5
1	5	5	5	5	5	3	5	3	5
1	5	5	2	5	3	3	5	5	5
1	4	4	4	4	4	5	5	5	5
1	4	5	5	5	5	5	5	5	5
3	5	4	4	5	5	5	4	4	5
2	4	5	5	4	4	5	5	5	4
1	2	1	2	1	2	2	5	1	1
2	2	5	2	3	2	5	5	5	3
1	5	5	4	4	4	4	5	5	4
1	5	5	4	4	4	4	5	5	4
2	5	5	5	5	3	5	5	5	5
2	5	5	5	4	4	4	4	4	4
2	5	5	5	5	5	4	5	5	5
2	2	5	2	3	2	2	5	4	3
1	3	5	3	5	2	5	5	5	3
2	3	3	3	1	3	3	2	3	4
2	2	5	5	5	4	5	5	5	5
1	5	5	5	5	5	5	5	5	5
2	2	2	4	2	3	3	5	3	5
2	3	2	4	2	2	2	4	4	3
2	5	5	5	5	4	5	5	5	5
2	5	2	5	3	2	2	4	4	5
1	4	4	5	4	4	5	5	5	5
1	3	5	5	5	3	5	5	5	4
2	3	3	3	4	3	3	5	5	5

2	4	3	4	3	2	5	5	3	4
3	1	3	3	3	3	3	4	4	4
2	4	5	5	5	4	5	5	4	5
2	4	5	5	5	5	5	5	5	5
1	5	5	1	5	1	4	1	5	5
2	4	5	5	4	4	5	5	5	5
1	3	1	2	2	2	5	2	5	5
2	1	1	1	1	1	1	1	1	1
1	4	4	5	3	4	5	5	5	4
1	1	5	5	5	5	5	5	5	5
2	2	5	5	5	2	4	5	5	5
2	2	4	4	4	4	4	4	4	4
2	2	5	5	5	2	5	5	5	5
1	3	5	5	5	3	5	5	5	5
1	2	5	3	5	5	5	5	5	5
1	2	5	3	5	5	5	5	5	5
1	5	5	5	5	5	4	5	4	5
2	2	3	5	3	5	5	4	3	3
2	3	3	3	3	4	3	4	3	2
1	5	5	5	4	4	5	5	5	4
2	4	5	2	4	4	3	4	5	5
2	4	3	4	3	2	3	3	4	3
2	5	5	4	5	3	2	5	5	5
2	5	5	5	4	4	4	5	5	5
2	4	4	5	4	4	4	4	4	4
2	4	1	5	1	1	2	2	2	1
1	4	5	5	5	5	4	4	4	5
2	2	3	5	4	3	4	5	5	5
2	3	5	5	5	2	5	5	5	4
3	2	2	5	5	1	5	5	5	5
2	2	3	3	3	3	5	5	5	3
2	4	5	5	5	5	5	5	5	5
1	4	4	5	4	4	4	5	5	4
1	3	3	4	3	3	3	5	5	4
2	2	3	5	3	2	2	5	5	3
2	3	3	4	4	3	3	3	4	4
2	5	4	5	4	2	3	4	5	5
2	3	3	5	3	2	3	3	2	5
2	1	2	5	1	1	3	4	4	1
1	5	5	5	5	5	5	5	5	5
2	4	3	3	3	4	3	3	1	5
2	5	5	5	5	5	5	5	5	5
1	4	5	3	5	4	3	5	5	5
1	3	5	5	3	2	5	4	4	5
1	5	5	5	5	5	5	4	5	5
2	5	5	5	5	4	5	5	5	5
1	5	5	5	5	5	5	5	5	5

2	3	3	5	3	5	5	4	5	3
2	4	5	5	5	4	4	4	5	5
2	5	5	5	5	4	3	3	1	5
2	5	5	5	5	4	5	5	5	5
1	3	3	2	3	3	3	2	5	3
2	5	5	5	5	5	3	4	5	5
2	3	2	5	5	3	5	5	2	1
2	5	5	5	4	3	2	4	3	5
2	5	5	5	5	3	5	5	5	5
2	3	5	5	5	3	5	5	5	5
2	5	5	4	5	4	5	5	5	5
2	3	4	3	4	4	4	5	5	5
1	3	4	4	5	5	5	5	5	5
2	4	5	5	4	4	4	5	4	5
2	5	5	5	5	5	5	5	5	5
2	1	2	2	2	2	1	5	2	5
1	1	5	2	4	1	1	5	5	5
2	3	5	5	5	3	5	5	5	5
1	2	2	2	2	2	3	4	2	2
1	5	5	5	5	4	5	5	4	5
2	2	4	5	5	4	2	4	5	5
2	5	5	5	3	5	5	3	5	5
1	1	5	5	2	1	5	5	5	5
2	5	5	1	3	1	2	5	3	5
2	5	5	5	4	4	4	5	5	5
2	5	5	4	4	5	5	4	4	5
2	3	3	5	5	3	2	3	3	5
2	2	5	5	2	2	5	5	5	4
2	3	4	4	4	3	3	4	4	4
1	2	5	5	3	3	1	3	5	3
2	5	5	5	5	5	5	5	5	5
2	5	5	5	5	5	5	5	5	5
2	5	5	5	5	5	5	5	5	5
1	1	3	3	3	3	3	3	3	3
2	3	4	4	5	3	5	5	5	5
1	5	5	5	5	3	5	5	5	5
2	1	5	5	5	3	5	5	5	5
2	1	4	5	3	3	3	5	4	3
2	3	5	5	5	4	5	5	5	5
1	5	3	2	3	3	3	4	4	5
2	3	3	5	3	3	3	4	4	5
2	4	5	2	2	2	2	2	5	5
2	4	5	5	4	4	5	4	5	5
2	3	2	2	2	2	2	2	1	1
2	4	5	5	5	5	5	5	5	5
2	5	5	5	5	5	3	5	5	5
2	4	5	5	5	1	1	5	5	5

2	5	5	5	5	5	5	5	5	5
1	5	5	2	5	2	5	5	5	3
2	5	5	5	5	3	3	4	5	5
2	1	4	2	1	1	2	5	5	1
2	5	5	4	3	3	1	2	3	2
2	5	5	4	4	3	4	5	5	5
2	4	2	2	2	2	1	1	1	5
2	2	2	2	3	2	4	3	5	5
1	4	3	3	3	3	3	4	4	3
2	5	5	5	5	5	5	5	5	5
2	1	5	5	5	5	5	5	5	5
2	5	1	1	1	1	3	1	2	4
2	5	5	5	5	5	5	5	5	5
1	3	1	1	1	1	1	1	5	5
1	3	5	5	5	4	5	5	5	4
2	3	4	5	3	3	5	5	5	3
2	5	5	5	5	5	5	5	5	5
1	5	1	3	1	1	3	5	3	5
1	5	5	5	5	5	5	5	5	5
2	5	5	5	5	5	5	5	5	5
1	5	5	5	5	3	5	5	5	5
1	1	5	4	5	4	4	5	5	5
1	2	5	5	4	4	5	5	5	5
1	3	4	4	4	4	4	5	5	5
1	5	5	3	5	2	3	5	5	5
1	5	5	5	5	5	5	5	5	5
1	4	4	3	5	4	4	4	5	5
2	4	3	5	3	3	4	5	5	5
1	5	5	2	5	2	1	2	5	5
1	4	2	1	3	1	5	5	5	5
2	4	5	5	3	3	5	5	5	5
2	3	5	4	3	2	3	5	5	3
1	3	5	4	5	4	5	5	5	5
2	2	3	5	3	3	3	5	3	3
3	1	1	2	1	1	3	2	2	1
2	5	5	5	5	5	5	5	5	5
2	4	5	3	5	3	5	5	5	5
2	5	5	5	4	4	5	5	5	5
2	5	5	5	5	5	4	5	5	5
3	5	5	5	5	5	5	5	5	5
1	2	5	5	5	5	5	5	5	5
1	4	5	5	5	3	5	5	5	5
2	2	4	5	5	5	4	5	5	5
2	5	5	5	5	5	4	5	5	5
2	5	3	5	4	3	3	5	5	5
2	5	3	2	5	5	5	5	5	5
2	4	3	3	1	1	2	4	2	4

2	2	2	2	2	2	3	3	2	1
2	3	1	3	1	1	5	5	3	4
1	3	2	3	3	3	5	5	5	5
2	4	4	5	3	4	4	5	5	5
1	1	3	1	1	1	3	5	5	1
1	3	4	3	5	3	4	3	3	5
1	3	3	2	3	3	2	4	3	5
1	4	4	5	4	5	5	5	5	5
2	5	5	4	5	5	5	5	5	5
2	5	5	4	4	4	5	5	5	5
2	2	2	3	2	3	3	5	5	2
1	5	5	5	3	1	4	4	3	5
1	5	5	5	5	4	5	4	5	5
2	4	5	5	4	4	5	5	5	5
1	5	5	5	5	5	5	5	5	5
2	5	5	5	5	3	1	2	5	5
2	3	3	3	3	2	3	4	4	2
1	4	5	5	5	5	5	5	5	5
1	3	2	5	2	1	2	3	2	3
1	5	5	5	4	3	5	5	5	5
1	3	5	5	5	4	5	4	4	4
2	5	5	5	4	3	3	5	3	4
2	3	4	5	3	3	3	5	4	5
1	3	5	5	5	3	3	5	5	5
2	5	5	5	5	5	5	5	5	5
2	5	5	4	5	4	5	5	5	5
2	4	4	5	5	5	5	5	5	5
2	4	5	5	4	4	5	5	5	5
3	2	3	3	2	1	3	4	4	2
1	3	3	3	3	3	4	4	4	3
2	5	5	5	5	5	5	5	5	5
2	4	4	5	4	4	4	4	4	4
2	5	3	3	3	3	2	3	3	3
2	5	2	2	2	2	2	2	2	5
1	4	4	3	4	3	5	5	5	5
2	5	5	5	5	5	5	5	5	5
2	4	5	4	5	4	5	5	5	5
2	2	2	5	3	2	1	3	3	1
2	5	5	5	4	1	3	4	3	5
2	5	4	4	4	4	4	5	3	4
2	4	5	5	4	4	5	5	5	5
2	4	3	5	5	3	3	4	5	3
1	2	2	1	2	3	3	3	3	5
2	5	5	4	5	4	4	4	5	5
2	4	4	5	4	3	3	5	5	4
2	1	5	5	5	3	5	5	5	5
2	3	3	5	4	4	5	5	5	5

2	3	3	3	3	2	5	5	4	5
2	1	2	1	5	1	4	4	4	2
2	4	4	3	4	4	5	5	5	5
2	4	3	4	3	3	3	4	3	3
1	4	4	4	4	4	4	5	5	5
1	5	1	5	1	1	1	5	1	1
3	3	5	5	4	3	5	5	5	5
1	3	5	4	4	5	3	5	5	3
1	5	5	5	5	4	4	5	5	5
2	4	3	5	3	3	5	5	3	5
2	5	5	5	5	5	5	5	5	3
2	1	1	2	1	2	4	5	1	2
2	4	4	5	4	2	3	3	5	3
2	2	3	3	3	3	3	4	4	3
1	3	5	5	4	4	5	5	5	5
2	5	5	4	5	5	5	5	5	5
2	5	3	3	2	2	4	4	5	4
1	3	2	2	2	2	2	2	2	2
2	3	5	2	5	3	5	5	5	5
2	4	5	5	4	4	5	5	5	5
2	5	4	4	4	3	5	5	5	5
2	5	5	5	4	2	3	5	5	5
2	5	4	5	5	4	5	5	5	5
2	4	5	5	5	4	5	5	5	5
1	5	5	5	5	5	5	5	5	5
1	5	5	5	5	5	5	5	5	5
1	5	5	5	4	1	4	5	5	4
2	5	4	4	3	4	3	5	4	5
2	2	2	3	4	3	1	3	4	5
1	3	3	5	3	3	2	4	3	3
2	3	3	5	2	4	5	5	5	3
2	3	1	2	2	1	2	2	1	4
3	3	4	5	4	4	4	5	5	5
1	3	3	4	2	2	3	3	3	3
3	5	5	5	5	4	5	5	5	5
2	3	5	3	5	3	5	5	5	5
1	5	5	5	4	4	3	5	2	5
2	5	5	5	4	3	5	5	5	4
1	4	1	1	4	3	2	3	5	4
1	5	5	5	5	4	5	5	5	5
2	5	5	5	5	5	4	4	5	5
1	4	5	5	5	3	5	5	5	5
1	4	5	1	5	1	1	2	5	5
2	4	3	5	4	3	5	5	5	4
2	2	1	2	1	2	3	2	2	1
1	5	4	4	4	4	4	5	5	5
1	5	5	5	4	4	5	5	4	5

2	5	5	3	5	3	2	5	5	5
2	3	3	3	3	2	3	4	5	5
3	4	4	4	4	3	5	5	5	4
2	3	3	5	3	4	3	5	5	3
2	4	4	3	4	4	4	5	5	5
1	4	5	5	3	2	2	3	4	5
2	1	5	5	5	5	5	5	5	5
1	4	4	3	4	4	3	4	4	5
2	5	3	2	5	3	2	5	5	5
1	5	4	3	4	3	3	4	4	5
1	4	3	3	3	3	4	4	3	4
3	5	5	5	5	5	5	5	5	5
2	4	4	5	4	4	3	5	5	5
2	5	5	4	5	5	5	5	5	5
2	5	5	5	4	3	4	5	5	4
2	5	5	5	5	4	5	5	5	5
2	4	4	4	4	2	2	3	5	4
2	4	4	4	4	4	4	4	4	4
2	4	5	5	5	5	5	5	5	5
2	2	5	2	5	2	2	2	5	5
2	3	5	5	5	3	3	5	5	3
1	5	5	5	3	5	5	5	2	5
2	5	5	5	5	5	5	5	5	5
2	3	5	5	5	4	5	5	5	4
2	5	3	5	2	2	2	4	2	3
1	5	5	5	4	5	5	4	5	5
2	3	2	5	2	3	5	5	5	5
2	3	2	5	2	3	5	5	5	5
2	4	3	4	4	4	4	4	4	5
1	4	3	1	2	3	1	2	2	2
2	3	5	4	5	4	4	5	5	5
2	5	5	5	5	3	5	5	5	5
2	5	5	4	5	5	5	5	5	5
2	4	5	4	4	3	5	5	5	5
2	5	5	5	5	5	4	5	5	5
1	3	3	3	2	2	3	3	2	3
1	1	2	5	1	1	5	5	5	5
1	5	5	5	5	5	5	5	5	5
2	4	5	5	4	3	2	4	4	5
2	5	5	5	5	5	4	5	5	5
2	4	3	5	4	3	3	4	4	4
2	5	4	5	4	4	4	4	5	4
2	5	5	5	4	4	5	5	5	5
2	4	5	4	4	2	5	5	5	5
1	3	4	4	2	3	3	4	3	4
1	4	5	5	4	3	4	5	5	5
2	4	2	2	3	1	3	5	5	5

2	5	4	4	4	4	4	4	4	4
2	5	5	5	5	5	5	5	5	5
2	5	4	2	3	3	3	3	2	4
1	5	5	5	5	4	5	5	5	5
1	5	5	3	5	3	3	5	5	5
3	5	5	5	5	5	5	5	5	5
2	4	5	5	5	5	5	5	5	5
1	2	3	2	3	2	2	3	3	3
1	2	3	3	2	2	5	5	5	3
2	4	3	5	5	3	4	4	5	5
2	4	5	5	5	4	5	5	5	5
2	1	1	1	1	1	1	1	5	1
2	5	5	5	5	4	5	5	5	5
1	5	5	5	5	4	3	4	3	5
2	5	5	1	5	5	5	5	5	5
1	5	5	5	5	3	2	4	5	5
1	5	5	5	5	4	5	5	5	5
1	5	5	5	5	5	5	5	5	5
2	5	5	5	5	4	5	5	5	5
2	5	5	5	5	4	5	5	5	5
1	3	3	5	3	2	3	5	5	3
1	5	5	4	5	4	3	5	5	5
1	5	5	5	5	5	5	5	5	5
2	5	5	5	3	3	4	5	3	5
2	5	5	4	5	4	5	5	5	5
2	4	3	3	3	3	3	3	2	2
2	4	2	2	2	2	3	4	4	2
2	2	4	4	4	5	4	5	4	5
1	1	5	5	4	4	3	4	5	4
2	3	4	4	5	3	5	5	5	5
1	5	5	5	5	4	5	5	5	5
1	4	5	2	5	4	5	4	5	5
2	5	4	5	5	4	5	5	5	5
1	5	5	4	5	4	5	5	5	5
3	3	3	5	3	2	4	5	5	3
2	3	3	5	1	2	2	5	5	5
1	2	5	5	5	5	5	5	5	5
1	5	5	5	4	4	4	5	5	5
2	3	3	3	2	3	4	3	4	4
1	4	4	4	5	3	5	5	5	4
2	4	4	4	4	4	4	5	5	5
1	4	5	3	4	3	5	5	5	5
1	5	5	5	4	4	3	3	4	5
1	5	5	5	5	5	5	5	3	5
2	2	5	5	5	5	3	5	5	5
2	4	5	5	4	4	4	4	4	5
2	5	5	5	4	4	5	5	5	5

2	5	5	4	4	4	4	4	4	5
2	3	3	4	5	3	5	5	4	5
2	2	4	3	4	4	3	5	5	5
1	5	5	5	5	5	5	5	5	5
2	5	5	5	4	3	5	5	5	5
2	3	5	5	5	3	5	5	5	5
2	4	5	1	4	4	4	4	5	5
2	3	2	3	1	2	2	4	3	4
2	3	5	5	3	2	3	3	5	3
2	3	4	5	4	4	4	4	4	4
2	3	3	3	4	3	3	4	4	5
1	4	5	5	5	3	5	5	5	5
2	3	3	4	3	3	5	5	5	5
2	2	5	3	5	5	3	5	3	4
2	5	5	5	5	4	5	3	5	5
1	4	4	4	4	4	5	5	5	5
2	3	5	5	5	4	3	3	3	5
1	5	5	4	4	4	5	5	5	4
2	2	1	5	1	2	2	5	3	1
1	2	3	5	5	3	3	5	5	5
1	1	5	5	5	3	5	3	5	5
2	3	2	2	2	1	3	5	4	3
2	3	5	5	5	3	3	5	5	5
2	3	3	5	3	4	3	3	5	3
2	4	4	4	5	1	5	5	5	5
1	4	4	3	4	4	4	5	5	5
2	3	5	5	5	3	5	5	5	5
2	4	4	5	4	2	5	5	5	4
1	3	5	5	3	3	3	5	5	5
2	3	4	4	4	4	5	5	5	5
2	5	5	5	5	5	5	5	5	5
1	5	5	5	4	4	5	5	5	5
1	5	4	5	4	5	4	4	3	5
1	1	5	3	4	3	4	5	5	5
2	3	5	3	5	3	2	3	3	5
2	5	5	4	5	4	5	5	5	5
2	4	4	4	5	4	3	5	5	5
1	5	2	2	2	3	2	3	3	2
2	1	5	5	5	5	5	5	5	5
3	5	5	4	5	4	3	3	5	5
2	3	4	5	3	2	5	5	4	2
2	4	5	3	5	4	3	5	5	5
2	2	5	5	5	1	3	5	5	5
1	1	5	5	5	4	5	5	5	5
2	4	4	5	4	4	3	3	4	5
2	2	2	2	1	2	2	4	2	1
2	5	2	5	2	2	2	5	4	5

2	2	2	5	3	2	1	1	1	5
2	3	5	5	4	3	5	5	5	5
2	3	2	5	2	2	2	5	3	5
2	3	5	2	2	5	3	5	3	5
3	4	5	5	5	5	5	5	5	5
2	5	5	5	5	2	3	4	3	5
2	2	2	5	2	2	2	4	5	5
2	3	3	3	2	3	1	4	2	3
2	4	4	4	4	4	4	4	4	4
2	4	5	5	4	4	5	5	5	5
2	4	5	5	4	4	5	5	4	5
2	5	5	5	5	4	5	5	5	5
2	3	2	5	2	2	1	3	2	5
2	4	5	4	4	4	5	5	5	4
1	2	5	5	5	5	5	5	5	5
2	3	5	5	3	3	5	5	5	4
1	5	3	3	5	3	5	5	5	4
2	3	3	4	2	2	3	5	4	2
1	5	2	5	2	3	5	5	1	2
2	3	5	5	4	1	4	5	5	5
1	4	4	4	4	4	4	4	4	4
2	2	2	5	3	2	5	5	5	3
1	4	3	4	4	2	5	5	5	4
2	4	4	4	4	4	4	5	4	5
2	1	3	5	2	1	3	5	5	5
2	4	4	4	4	4	5	5	4	5
2	5	5	4	5	4	4	5	5	5
2	4	5	5	5	5	5	5	5	5
2	5	5	5	5	5	5	5	5	5
2	5	5	5	5	5	5	5	5	5
2	3	5	5	5	5	5	5	5	5
2	1	2	2	2	2	5	2	2	5
2	2	2	2	2	2	5	5	5	2
1	5	5	3	5	3	4	5	5	5
2	5	5	5	5	5	4	5	5	5
1	5	5	5	5	3	4	4	5	5
1	4	4	4	4	4	4	5	5	5
2	3	5	5	5	5	4	5	5	5
2	4	4	4	5	4	4	4	5	5
2	5	5	5	5	4	5	5	5	5
2	5	5	5	4	4	5	5	4	5
1	2	4	5	3	3	4	5	5	3
3	3	3	3	3	3	3	5	5	5
2	4	5	4	5	3	5	5	5	5
2	3	5	5	5	3	5	5	5	5
2	3	4	3	3	3	3	4	4	4
1	4	4	5	4	3	5	5	5	3

1	3	3	3	3	3	4	4	3	4
2	1	4	2	3	3	3	5	3	2
3	5	5	5	5	5	5	5	5	5
2	5	5	4	4	4	4	5	5	5
2	5	1	5	1	1	1	2	1	5
1	3	4	2	4	4	3	5	4	5
1	5	5	4	5	4	5	5	5	5
1	4	4	3	3	3	4	4	4	4
2	5	5	5	4	4	4	5	5	5
2	5	5	5	5	5	5	5	5	3
2	4	4	4	4	5	5	5	5	5
2	5	5	4	3	4	5	5	5	5
1	5	5	5	4	5	5	5	5	5
2	5	5	4	4	3	5	5	5	5
2	3	3	5	3	3	3	5	5	5
1	4	4	4	4	4	5	5	4	4
2	4	3	5	3	2	4	5	4	4
2	2	3	4	4	2	4	5	5	4
1	3	3	3	3	3	3	5	5	5
1	5	5	5	5	4	5	5	5	5
2	5	5	5	5	5	5	5	5	5
2	1	5	5	5	3	2	3	5	5
2	4	3	5	3	3	5	5	4	5
2	5	5	5	5	5	5	5	5	5
2	4	5	5	5	5	5	5	5	5
1	4	4	4	4	4	4	5	5	5
3	4	5	5	5	3	4	5	5	4
2	2	4	2	4	2	3	4	4	5
2	4	3	3	3	2	3	4	4	4
2	4	5	4	4	4	3	4	4	4
2	1	1	5	1	1	2	5	5	5
1	1	1	2	3	1	1	5	5	5
2	2	1	5	1	1	3	3	1	3
2	5	5	5	4	4	4	5	4	5
2	3	5	5	4	4	5	5	5	5
2	4	4	3	5	5	3	5	5	5
1	4	4	4	4	3	3	4	4	5
2	4	5	5	4	4	5	5	5	5
2	3	5	3	4	4	3	5	5	5
2	3	5	5	5	3	5	5	4	3
2	5	4	4	4	4	5	4	4	5
2	5	5	3	5	3	2	3	4	5
1	5	5	5	5	5	5	5	5	5
2	5	5	5	5	4	5	5	5	5
2	5	5	5	5	3	5	5	5	4
2	3	4	4	5	4	4	5	5	5
1	5	2	2	3	3	1	1	1	2

2	5	5	5	5	5	3	4	3	5
2	5	5	3	5	4	5	5	5	5
2	5	5	5	5	3	5	5	5	4
2	5	5	5	4	3	5	5	4	5
1	5	5	4	4	4	3	3	4	5
1	5	3	5	3	4	5	5	2	3
1	3	4	3	3	2	3	3	2	2
1	5	5	5	3	4	3	4	5	5
2	5	4	5	2	1	3	3	3	3
2	4	5	5	5	5	5	5	5	5
3	5	5	5	5	3	5	4	5	5
1	3	5	2	4	3	4	5	5	5
3	5	5	5	5	5	5	5	5	5
2	4	4	3	4	3	3	4	3	5
2	4	5	5	5	4	5	5	5	5
2	5	5	5	5	4	5	5	5	5
2	5	5	5	5	5	5	5	5	5
2	3	3	2	3	2	4	5	3	3
1	2	5	5	4	3	5	5	5	5
2	5	4	5	4	4	5	5	5	5
2	5	5	5	5	4	5	5	5	5
2	3	5	5	5	4	4	5	5	5
3	5	4	5	4	4	5	5	5	4
2	5	5	5	5	5	5	5	5	5
1	3	3	5	3	3	2	4	3	5
1	4	5	5	5	4	4	5	5	5
1	3	4	3	4	3	3	3	3	3
2	5	5	4	5	4	4	4	5	5
1	4	2	5	5	5	5	5	5	4
2	4	4	5	5	5	3	5	5	5
2	5	5	5	5	5	5	5	5	5
2	4	5	4	4	2	4	5	5	5
3	4	5	4	4	2	2	3	4	5
2	4	4	4	4	4	4	5	5	5
2	4	4	5	4	4	4	4	5	5
1	5	5	5	4	4	4	4	4	4
2	3	3	3	3	2	3	2	5	2
1	4	5	5	5	4	5	5	5	4
2	5	5	3	5	5	2	3	3	5
1	4	5	5	5	4	4	5	5	5
2	1	5	5	5	4	5	5	5	5
2	3	5	3	5	3	5	5	5	5
2	4	2	3	4	3	2	3	3	5
3	1	5	5	5	5	5	5	5	5
2	3	4	3	2	3	4	3	2	3
1	2	3	2	2	3	3	4	5	5
2	2	1	5	1	1	5	5	5	1

2	4	5	4	4	4	4	4	4	4
2	3	3	2	3	2	3	3	3	4
2	1	2	2	2	2	2	3	2	2
2	4	5	5	5	4	3	5	5	5
1	5	3	3	3	1	1	5	4	2
2	2	5	5	4	4	4	5	5	3
1	2	4	3	3	3	5	5	5	3
2	5	5	5	5	3	5	5	5	5
1	5	5	1	5	1	5	5	5	4
2	5	5	5	5	5	5	5	5	5
1	4	5	5	5	4	5	5	5	5
2	5	5	5	5	5	5	5	5	5
2	4	3	5	3	3	5	3	3	5
2	3	2	4	1	2	2	5	1	1
3	4	5	5	5	3	5	5	3	4
2	4	5	5	5	4	4	4	5	5
2	5	5	5	5	4	4	4	5	5
2	5	5	5	5	4	5	4	3	5
2	5	5	3	3	3	4	4	5	5
2	5	5	5	5	5	5	5	5	5
1	5	5	5	5	5	3	5	5	5
2	5	5	3	5	2	5	2	5	5
2	5	5	5	5	5	3	5	5	5
1	5	5	5	5	4	4	5	5	5
2	4	5	5	5	5	5	5	5	5
2	3	3	3	4	3	3	3	4	5
2	4	4	5	4	2	3	4	4	2
2	3	3	4	3	3	5	4	5	3
3	5	5	5	5	5	5	5	5	5
1	3	3	4	3	3	4	5	5	3
1	5	5	4	5	5	4	5	5	5
2	3	2	3	4	2	5	3	5	1
2	3	5	5	5	4	5	5	5	5

EVASI_10	EVASI_11	EVASI_12	EVASI_13	EVASI_14	EVASI_15	EVASI_16	EVASI_17	EVASI_18	EVASI_19
5	3	3	5	3	3	5	3	4	5
5	3	5	5	5	3	4	3	3	5
5	3	3	3	3	3	4	4	5	5
5	5	5	5	5	5	3	5	5	3
5	4	4	2	3	3	4	4	5	4
5	5	5	5	5	5	5	5	5	5
3	2	2	3	4	4	3	3	3	3
2	2	1	2	1	3	2	1	2	1
5	5	4	5	5	1	4	5	5	5
5	3	3	5	5	5	5	5	5	5
1	5	2	5	1	5	2	4	3	4
5	2	2	5	3	3	5	2	5	5
5	1	1	3	1	3	3	1	3	1
5	5	5	5	4	3	3	3	3	4
3	5	5	3	3	4	3	5	5	5
5	4	5	4	4	5	5	4	4	4
4	2	2	4	2	1	4	2	2	2
5	5	4	3	4	4	4	5	4	5
5	4	4	3	5	3	3	4	4	4
5	4	4	5	5	4	5	4	4	4
5	4	5	5	5	5	3	4	5	5
5	5	3	5	5	5	5	5	5	5
5	3	3	5	5	5	5	3	5	3
5	3	3	5	5	5	5	3	3	5
5	3	3	5	5	5	5	5	5	5
5	5	4	4	4	5	5	5	4	4
5	5	5	4	4	4	4	5	5	5
5	1	1	2	4	3	5	2	4	1
3	2	4	5	1	3	3	5	5	5
5	5	4	4	4	4	4	4	4	4
5	5	4	4	4	4	4	4	4	4
5	3	4	3	5	1	5	3	5	4
4	4	4	3	4	4	4	4	4	4
5	5	5	5	5	5	5	5	5	5
2	1	1	5	2	2	3	3	5	3
3	5	3	3	3	3	5	5	5	5
3	3	2	1	4	1	2	3	2	2
5	5	5	5	5	5	5	5	5	5
5	5	5	5	5	5	5	5	5	5
5	3	3	4	5	4	1	3	5	5
3	3	2	5	3	2	3	2	3	3
5	5	5	5	5	5	5	5	5	5
5	1	2	4	3	3	5	2	5	3
5	5	5	4	5	5	5	5	5	5
5	5	4	5	5	3	5	5	5	5
5	3	3	5	3	3	3	3	3	5

3	4	3	2	4	2	4	4	5	2
4	4	4	2	4	4	4	4	2	4
4	5	3	5	5	3	4	5	3	3
5	5	5	5	5	5	5	5	5	5
5	5	5	5	5	5	5	5	5	1
5	5	5	4	4	5	5	5	5	4
5	3	4	4	2	3	3	4	4	5
1	5	1	1	1	1	1	1	1	1
4	5	5	5	5	5	5	5	5	5
5	5	5	5	5	5	5	5	5	5
5	3	3	5	5	5	5	3	3	3
5	5	5	5	5	4	5	5	5	5
5	5	5	3	3	3	5	5	5	5
5	5	5	2	5	3	5	5	5	5
5	5	5	5	5	5	5	5	5	5
5	5	5	5	5	5	5	5	5	5
5	4	4	5	5	4	5	4	4	4
3	3	3	4	3	3	3	5	3	4
3	3	2	5	3	3	4	3	4	3
4	4	5	5	5	5	5	5	5	5
5	4	4	3	4	1	5	3	5	5
5	2	2	2	2	5	3	2	3	2
5	2	2	3	5	1	5	1	2	1
5	5	5	5	5	3	5	5	5	5
4	3	3	4	4	4	4	3	4	4
5	1	1	3	1	1	4	3	5	1
4	4	4	4	4	5	4	4	4	4
5	5	4	3	4	5	5	5	5	5
5	5	5	3	5	5	5	5	5	4
5	5	5	4	5	2	5	5	5	5
3	5	3	3	3	5	3	5	4	5
5	5	4	5	5	5	5	5	5	5
5	5	5	5	5	4	5	5	4	5
5	3	3	1	4	4	4	3	3	4
3	2	5	5	5	2	3	5	5	5
4	3	4	3	4	5	5	3	3	2
4	3	5	3	5	5	5	3	5	3
5	3	4	5	5	3	5	5	2	3
1	3	1	2	2	4	1	3	5	3
5	5	5	5	5	5	5	5	5	5
5	1	3	5	3	3	5	1	5	1
5	5	5	5	5	5	5	5	5	5
5	3	5	2	3	1	3	4	3	3
5	2	2	5	5	5	5	5	5	5
5	5	5	3	5	5	3	5	5	4
5	5	5	5	5	4	5	5	5	5
5	5	5	5	5	5	5	5	5	5

3	5	5	3	3	3	3	5	5	5
5	5	3	5	5	3	5	4	3	3
5	3	5	1	4	1	5	2	1	1
5	5	5	5	5	5	5	5	5	5
5	2	2	5	3	3	4	2	3	2
5	3	1	5	3	5	5	2	3	1
5	5	3	5	1	3	5	3	3	5
5	3	3	5	5	1	5	3	3	3
5	5	5	5	4	5	5	5	5	5
5	4	3	3	5	5	4	5	5	4
5	4	5	5	5	5	5	4	5	5
5	4	4	2	5	5	5	4	4	3
5	5	5	5	5	5	5	5	5	5
5	4	4	5	4	4	5	5	5	4
5	5	5	5	5	5	5	5	5	5
5	5	5	2	5	1	5	5	5	5
5	5	1	1	1	1	5	5	5	5
5	5	5	2	5	1	2	5	5	5
2	2	2	2	2	2	5	2	2	2
5	5	5	5	5	5	5	5	5	5
5	3	3	3	4	5	4	3	5	3
5	3	3	5	5	3	5	5	3	3
5	3	5	3	3	4	5	5	5	2
5	1	2	5	5	5	5	2	5	2
5	4	3	4	4	5	5	5	4	5
4	5	3	5	5	1	5	5	3	4
5	1	3	1	1	3	5	1	3	1
4	4	2	3	5	4	5	5	4	5
4	2	2	4	4	4	4	3	4	4
5	1	3	5	3	1	4	3	5	3
5	5	5	5	5	5	5	5	5	5
5	5	5	5	5	5	5	5	5	5
5	5	5	5	5	5	5	5	5	5
5	3	3	3	3	3	3	3	3	3
5	5	5	3	5	5	5	5	5	5
5	5	5	5	5	5	5	5	5	5
5	5	5	5	5	1	5	5	5	5
5	4	5	3	2	3	3	3	5	3
5	5	5	5	5	1	5	5	5	5
5	3	3	5	5	5	2	4	4	4
5	2	2	5	3	3	3	2	5	3
5	5	5	4	5	1	1	5	5	5
5	3	4	3	4	5	4	4	5	5
1	1	1	3	2	2	2	1	1	1
5	5	5	4	5	1	5	5	5	5
5	3	5	5	5	3	5	3	5	5
5	1	1	3	1	5	5	3	5	3

5	5	5	5	5	5	5	5	5	5
5	3	2	5	3	3	5	3	5	5
5	3	2	5	4	5	5	5	2	2
1	1	2	5	1	1	1	4	5	5
3	1	1	2	1	2	3	1	2	1
5	4	5	5	3	4	5	4	5	5
5	2	1	5	1	1	5	1	1	1
4	3	3	2	5	4	3	2	5	3
3	3	3	5	3	3	3	3	4	3
5	5	5	5	5	5	5	5	5	5
5	5	5	5	5	5	5	5	5	5
4	1	1	4	1	5	4	1	1	1
5	5	5	4	5	5	5	5	5	5
5	5	3	3	3	4	5	5	5	5
5	4	4	3	5	4	4	4	4	5
3	5	5	5	5	5	5	5	5	5
5	5	5	1	5	5	5	5	5	5
5	1	3	5	1	5	5	1	3	3
5	5	5	5	5	1	5	5	5	5
5	5	4	3	5	4	5	4	5	5
5	3	3	1	5	3	5	3	5	5
1	3	4	5	5	4	5	4	5	5
5	4	4	3	4	4	5	4	5	5
5	4	5	5	5	5	5	5	5	5
5	2	2	5	3	1	3	3	2	2
5	5	5	5	5	5	5	5	5	5
5	4	5	3	5	5	5	5	5	5
5	3	4	3	4	4	5	5	5	5
5	1	1	3	5	5	5	1	2	1
5	2	1	5	1	5	3	2	4	3
5	3	3	3	5	5	1	5	5	5
3	3	3	4	3	2	3	3	3	3
5	5	5	3	5	5	3	5	5	5
3	3	3	5	3	3	3	3	3	3
1	2	1	2	1	1	3	2	2	2
5	5	5	5	5	5	5	5	5	5
5	5	5	5	5	5	5	5	5	5
5	4	4	5	5	5	5	5	5	5
5	5	5	5	5	5	5	5	5	5
5	5	5	5	5	1	5	5	5	5
5	5	5	1	5	1	5	5	5	5
5	3	3	5	5	5	5	3	5	5
4	5	5	5	5	3	4	5	5	5
5	5	5	5	5	5	5	5	5	5
5	4	3	4	4	1	5	4	5	4
5	5	4	5	5	5	5	5	1	5
5	1	1	3	2	1	5	1	2	2

2	2	2	3	2	2	2	2	2	2
5	3	1	5	1	2	5	2	5	2
3	3	5	5	3	5	5	5	5	5
5	4	5	3	5	3	5	5	4	3
3	3	3	5	1	3	2	3	4	3
5	4	5	2	3	1	3	3	5	3
5	3	3	5	3	1	5	3	3	3
5	5	5	3	5	1	5	5	5	5
5	5	5	5	5	5	5	5	5	5
5	5	5	3	5	1	5	5	5	5
3	3	2	3	3	4	4	3	5	5
5	3	3	2	3	3	4	2	3	2
5	5	4	5	5	5	4	5	5	5
5	5	4	5	4	5	5	5	5	5
5	5	5	2	5	1	5	5	5	5
5	1	1	5	5	5	5	1	1	3
3	2	3	4	2	4	5	3	3	3
5	5	5	3	5	5	5	5	5	5
3	2	1	5	1	3	5	2	2	2
4	5	5	3	5	3	2	5	3	2
4	4	4	4	4	4	4	4	3	4
5	4	3	5	4	4	4	4	4	4
5	3	3	4	5	4	3	3	5	3
5	3	3	3	3	5	3	3	5	5
5	5	5	5	5	5	5	5	5	5
5	4	5	3	5	5	5	5	5	5
5	4	4	4	5	4	5	5	5	5
5	4	5	4	5	5	5	5	5	5
2	3	3	3	2	2	2	2	4	3
3	3	3	2	3	3	3	4	4	4
5	5	5	5	5	5	5	5	5	5
4	4	3	3	3	4	4	4	4	4
5	2	2	4	3	3	5	3	5	3
5	5	2	2	2	2	5	5	2	2
5	5	5	5	4	1	5	5	5	5
5	5	5	4	5	5	5	5	5	5
5	5	5	3	5	1	5	5	4	5
1	2	1	4	1	3	1	3	3	1
5	2	2	1	1	3	4	1	3	2
5	5	4	4	4	5	5	5	4	4
5	5	5	4	5	5	5	5	5	5
5	2	2	1	4	4	5	3	2	4
5	5	1	1	1	3	1	1	1	1
5	3	3	4	4	4	4	3	4	3
4	4	4	5	4	5	5	4	4	4
5	5	5	5	5	1	5	5	5	5
5	4	5	1	2	5	4	4	5	3

5	3	4	4	2	2	5	3	5	4
2	1	3	5	1	1	3	4	4	4
5	5	5	5	5	5	5	5	5	5
3	4	3	3	3	3	3	5	3	3
5	5	5	3	5	5	5	5	5	5
1	1	1	5	1	5	5	1	1	1
5	3	3	5	4	5	5	4	5	4
2	4	3	5	3	1	3	4	4	3
5	4	4	5	5	5	5	4	4	5
5	3	4	3	5	4	5	5	4	5
5	5	4	3	5	5	5	5	5	5
1	3	1	1	2	3	5	4	5	1
5	3	3	1	3	5	5	3	3	3
3	3	3	3	3	2	2	3	3	5
5	3	5	3	4	1	5	5	5	5
5	5	5	4	5	5	5	5	5	5
5	4	3	3	3	3	5	3	4	4
4	2	1	2	1	1	2	2	1	1
5	4	5	5	4	5	4	5	5	5
5	5	5	3	5	1	5	5	5	5
4	4	5	5	5	5	5	5	5	5
5	2	4	3	3	5	5	5	5	5
5	4	5	1	4	4	5	5	5	4
5	5	5	5	5	5	5	5	5	5
5	5	5	5	5	5	5	5	5	5
5	5	5	5	5	5	5	5	5	5
5	3	5	5	4	1	5	4	5	5
5	3	3	3	3	3	5	3	5	3
5	1	1	3	1	1	5	2	2	1
5	3	3	3	4	5	5	3	3	3
5	5	3	4	3	5	5	5	5	5
3	1	1	2	2	2	3	2	2	2
5	4	4	2	4	1	4	4	5	5
3	2	2	3	3	1	5	3	4	2
5	4	5	5	5	5	5	4	5	4
5	5	5	5	5	5	5	5	5	5
5	2	1	5	3	1	4	2	4	2
5	5	5	5	4	4	4	4	5	5
4	3	3	4	3	2	5	2	2	3
5	5	4	5	5	5	5	5	5	5
5	4	4	3	3	2	5	3	3	2
5	3	5	5	5	5	5	4	5	5
5	1	1	5	5	1	5	1	3	1
5	4	5	3	3	3	4	5	4	5
1	3	2	2	2	2	2	3	3	3
5	5	4	5	5	1	5	5	5	4
5	5	5	4	4	5	5	4	5	4

5	2	3	3	4	2	5	2	5	3
5	3	2	4	3	2	2	3	4	3
5	5	5	4	5	5	5	5	5	5
1	3	3	5	2	1	2	3	1	5
5	5	5	4	5	5	5	5	5	5
5	1	1	5	1	3	5	1	3	1
5	5	5	5	5	5	5	5	5	5
5	4	3	5	5	4	5	5	4	5
5	2	2	5	2	5	5	2	3	2
5	3	3	3	5	3	4	4	3	4
3	2	3	4	3	4	4	3	4	3
5	5	5	1	5	5	5	5	5	5
5	3	3	3	5	5	4	4	5	4
5	5	4	4	5	5	5	5	5	5
5	4	4	5	4	4	5	5	1	4
5	5	5	3	5	5	5	5	5	5
4	2	3	3	2	4	4	2	3	4
4	4	3	3	4	3	3	4	4	4
5	5	5	4	5	5	5	5	5	5
5	3	2	5	3	5	5	2	5	3
5	5	3	5	3	1	3	3	5	5
5	4	3	5	5	5	5	5	5	5
5	5	5	5	5	5	5	5	5	5
4	5	4	3	5	3	2	5	4	5
5	1	1	5	3	2	2	1	5	1
5	5	5	4	5	5	4	5	5	4
3	5	4	3	4	5	3	5	5	5
3	5	4	3	4	5	3	5	5	5
4	5	4	4	5	5	5	4	4	4
3	1	1	5	1	3	4	1	1	1
5	5	5	3	5	1	3	4	4	3
5	5	5	5	5	5	5	5	5	5
5	5	4	5	5	5	5	5	5	5
4	4	5	2	4	1	3	5	5	5
5	4	4	5	4	5	5	4	5	5
3	1	2	1	2	3	3	2	2	2
5	1	3	3	5	4	3	4	5	5
5	5	5	5	5	5	5	5	5	5
5	4	3	5	4	5	4	3	4	4
5	4	4	5	5	5	5	4	5	5
5	3	3	3	4	5	5	4	5	4
4	5	4	4	4	4	5	5	5	4
5	5	5	5	5	5	5	5	5	5
5	5	5	5	5	5	5	5	5	5
5	4	4	4	4	4	1	4	4	4
4	5	5	4	5	5	5	5	5	5
5	2	3	3	5	3	5	2	2	3

4	4	4	4	4	4	4	4	4	4
5	5	5	5	5	1	5	5	5	5
3	5	3	5	4	3	4	5	4	3
5	5	5	3	5	5	5	5	5	5
5	3	3	5	3	5	5	3	5	5
5	5	5	5	5	5	5	5	5	5
5	5	5	5	5	5	5	5	5	5
3	2	2	3	3	4	3	2	3	3
2	2	3	3	2	3	2	3	3	5
5	2	2	1	1	1	5	3	4	3
5	5	5	4	5	5	4	5	5	5
1	1	1	5	1	1	1	1	1	1
5	5	5	5	5	5	5	5	5	5
5	5	3	5	5	4	1	3	3	2
5	5	4	3	5	3	5	5	5	4
5	2	2	5	3	3	5	2	1	3
5	5	5	5	5	5	5	5	5	4
5	5	5	5	5	5	5	5	5	5
5	5	5	5	5	5	5	5	5	5
5	5	5	5	5	1	5	5	5	5
5	3	3	3	3	3	3	3	5	3
5	4	3	3	5	1	5	5	3	3
5	5	5	5	5	5	5	5	5	5
5	3	2	3	5	3	3	3	5	3
5	5	5	4	5	3	5	5	4	5
3	2	3	2	3	3	2	2	2	3
2	3	3	4	3	1	2	4	4	4
5	5	4	5	4	4	5	5	4	4
5	3	3	3	5	5	5	4	4	4
5	4	4	3	4	4	3	4	4	5
5	5	5	5	5	5	5	5	5	5
5	5	3	1	3	5	3	5	5	5
5	5	5	5	5	5	5	5	5	5
5	4	4	4	5	4	5	5	5	5
5	4	3	2	4	5	5	5	5	5
5	5	4	1	4	3	3	3	4	3
5	5	5	5	5	5	5	5	5	5
5	4	4	3	5	5	5	4	5	5
5	4	4	3	4	2	5	4	3	4
5	4	5	5	5	4	3	4	5	5
5	4	4	4	5	4	5	5	5	5
5	5	5	5	5	5	5	5	5	5
5	3	2	5	5	5	5	4	3	3
5	5	5	5	5	3	5	5	5	3
5	5	5	5	5	5	5	5	5	5
5	4	4	2	5	2	5	5	3	3
5	5	5	4	5	5	5	5	5	5

5	4	4	5	4	4	5	4	4	4
5	4	4	5	3	5	5	4	5	5
5	5	4	4	5	5	2	5	4	5
5	5	5	1	5	5	5	5	5	5
5	3	5	4	5	5	5	5	5	5
5	4	5	5	5	5	4	5	4	4
5	3	4	3	4	4	4	4	5	5
5	1	1	2	4	2	5	3	4	3
5	2	2	5	2	2	5	3	5	2
5	4	3	5	4	4	5	4	4	4
3	3	3	3	4	1	3	3	5	4
5	5	5	3	5	1	3	5	5	3
4	4	5	3	4	4	4	5	5	5
5	3	4	3	3	3	3	3	3	4
5	5	4	2	5	4	5	5	3	4
5	5	5	5	5	1	5	5	5	5
1	3	2	5	3	3	5	4	3	3
5	5	5	3	5	4	3	5	5	5
1	1	1	4	2	3	1	4	5	5
5	3	5	3	2	3	3	3	5	2
5	5	5	1	5	5	5	5	5	5
3	2	3	1	1	1	3	3	5	3
5	3	3	4	5	5	5	4	4	4
3	3	3	5	3	5	5	3	3	3
5	2	2	5	1	4	3	5	5	3
5	5	4	4	5	5	5	5	5	5
5	5	5	5	5	5	5	5	5	5
4	4	5	5	4	3	5	5	5	4
5	3	2	2	3	2	3	2	5	2
5	5	5	3	5	3	4	5	5	5
5	5	5	5	5	1	5	5	5	5
5	5	4	5	5	1	5	4	4	4
5	4	4	5	4	4	5	4	4	4
5	3	5	1	5	5	3	5	5	5
5	5	3	4	3	4	5	3	3	3
5	5	4	5	5	5	5	5	5	5
5	4	5	5	5	5	5	4	5	5
2	2	2	1	1	2	5	2	2	2
5	5	5	5	5	4	5	5	5	4
5	3	3	3	3	3	5	3	3	3
2	3	3	1	3	4	3	4	5	4
5	4	5	1	4	3	5	5	5	5
5	3	4	5	5	1	5	3	5	5
5	4	4	1	5	5	3	4	5	5
5	4	3	5	4	5	5	3	3	3
1	5	3	5	3	1	5	3	4	3
5	2	1	5	2	1	3	2	3	2

5	3	1	1	2	5	1	2	1	1
5	5	3	5	5	5	5	5	5	5
5	1	2	5	3	3	5	2	2	2
3	3	3	2	5	3	2	3	4	3
5	5	5	3	5	5	5	5	5	5
5	2	3	5	2	2	5	2	4	3
4	1	5	3	5	4	5	1	5	3
3	1	1	3	3	2	3	1	1	1
3	3	3	5	4	4	4	4	4	4
5	5	5	5	2	5	5	5	5	5
5	2	4	4	5	5	5	3	3	3
5	5	5	5	5	1	5	5	5	5
3	2	1	1	3	1	5	1	5	2
4	4	4	5	4	5	4	5	5	4
5	5	5	5	5	5	5	5	5	5
4	5	4	2	4	3	3	5	4	5
5	5	5	2	5	3	3	5	5	3
2	2	3	3	3	2	5	3	3	2
5	5	3	5	3	5	3	5	2	3
5	3	4	5	5	5	5	5	5	5
5	4	4	3	5	5	5	5	5	5
2	3	4	1	5	1	3	3	5	5
4	3	2	3	4	2	4	4	4	4
5	4	4	1	5	1	5	4	4	4
5	3	3	5	1	1	5	5	5	5
5	5	5	5	4	5	3	5	4	4
5	5	5	5	5	5	5	5	5	5
5	5	5	5	5	5	5	5	5	5
5	5	5	5	5	5	5	5	5	5
5	5	5	5	5	5	5	5	5	5
4	5	5	3	5	1	5	5	5	5
5	2	2	5	1	1	5	5	4	2
3	4	2	3	3	3	3	4	5	5
5	2	2	5	5	5	5	3	5	3
5	5	5	5	5	1	5	5	5	5
5	5	5	3	5	5	5	5	5	5
5	4	4	3	5	5	5	5	5	5
5	5	5	3	5	4	5	5	4	5
5	4	4	4	5	4	5	4	4	5
5	5	5	5	5	3	5	5	5	5
5	5	5	4	5	5	5	5	4	5
3	3	4	2	5	5	3	5	5	5
5	5	5	5	5	5	5	5	5	5
5	5	5	5	5	5	5	5	5	5
5	4	5	3	5	5	5	5	5	5
4	4	4	4	4	1	4	4	4	4
3	3	5	5	5	1	3	5	5	5

3	3	3	2	3	3	3	3	4	4
3	2	2	1	3	2	4	2	3	3
5	5	5	4	5	5	5	5	5	5
5	4	3	4	3	4	5	4	4	4
4	1	1	5	1	1	5	1	1	1
5	3	3	2	4	3	5	3	3	3
5	5	5	3	5	5	5	5	5	5
4	3	3	4	4	4	4	3	4	4
5	4	3	3	4	1	5	4	4	4
5	3	5	3	5	2	3	5	5	5
5	3	3	3	4	4	5	5	5	4
5	4	5	5	5	1	5	5	3	5
5	5	4	2	5	4	5	5	5	5
5	5	5	3	5	5	5	5	5	5
5	3	3	2	5	5	3	3	5	3
4	4	3	4	4	3	4	4	4	4
4	3	3	3	3	3	4	3	4	4
5	5	5	3	3	1	2	4	4	4
5	3	3	2	5	3	5	3	5	3
5	5	5	1	5	1	5	5	5	5
5	5	5	5	5	5	5	5	5	5
5	1	2	2	5	5	5	2	1	1
5	3	3	3	5	5	5	3	5	4
5	5	5	3	5	5	3	5	5	5
5	5	5	2	5	5	5	5	5	5
5	5	5	5	5	5	5	5	5	5
3	4	5	3	3	1	5	5	5	5
5	2	2	3	5	5	5	2	4	3
4	2	2	2	3	3	4	3	4	4
5	3	3	3	4	3	3	3	3	3
5	3	3	1	5	2	5	2	5	3
5	1	1	5	1	5	5	1	3	3
1	4	3	5	5	1	4	5	1	1
5	4	4	3	4	4	4	4	5	5
5	5	4	4	5	5	5	5	5	5
5	3	3	5	5	5	5	3	5	5
5	2	3	5	5	5	3	4	3	3
5	4	4	5	5	4	5	5	3	5
5	3	3	1	4	4	5	3	4	5
5	5	4	3	3	3	1	5	5	5
5	5	4	2	4	5	5	5	5	5
5	2	2	5	3	3	5	2	3	2
5	5	4	3	4	3	5	4	5	5
5	5	5	5	5	5	5	5	5	5
5	5	5	4	4	4	4	4	4	4
5	5	5	5	5	4	5	5	5	5
5	1	1	2	2	1	5	1	1	1

5	3	2	2	3	5	5	3	3	2
5	5	5	3	5	5	5	5	5	5
5	3	5	5	5	5	5	4	5	5
5	4	5	3	4	5	3	5	5	5
5	3	3	3	5	1	3	3	4	4
3	4	4	5	3	5	4	4	4	3
3	3	2	3	2	1	5	3	4	3
5	2	3	1	5	4	5	4	4	3
3	2	3	3	1	5	5	3	3	3
5	4	4	3	5	5	5	4	3	3
5	4	3	5	5	5	5	4	4	5
5	4	5	5	4	1	5	4	4	5
5	5	5	5	5	5	5	5	5	5
4	2	3	2	5	3	4	3	4	3
5	5	5	5	5	5	5	5	5	5
5	5	5	5	5	5	5	5	5	5
5	5	5	3	5	5	5	5	5	5
3	2	3	5	3	1	1	3	2	5
5	3	4	1	5	5	5	5	5	5
5	5	5	5	5	5	5	5	5	5
5	5	5	5	5	1	5	5	5	5
5	5	5	3	4	5	3	5	5	5
4	5	4	3	5	5	5	5	5	4
5	5	5	3	5	5	5	5	5	5
5	3	3	4	4	4	5	3	4	3
5	5	4	5	5	3	5	4	4	4
5	3	4	5	5	4	4	2	3	2
5	4	4	5	4	1	5	3	3	3
5	5	5	5	5	5	5	5	5	4
5	3	3	5	5	5	5	3	3	3
5	4	4	5	5	5	5	4	5	5
5	3	4	5	4	3	3	4	4	4
5	3	2	2	2	1	5	3	5	3
5	5	5	5	5	5	5	5	5	5
5	5	5	5	5	5	5	5	5	5
5	4	3	5	4	4	5	5	4	4
5	5	3	3	5	3	3	2	5	5
5	3	5	3	4	5	5	5	5	5
5	2	2	5	3	1	5	3	3	3
5	4	4	4	5	4	5	5	5	5
4	5	5	3	5	1	5	5	5	5
5	4	5	4	5	3	4	4	4	5
5	2	2	3	3	5	5	3	2	2
5	5	5	5	5	5	5	5	5	5
2	4	3	5	5	4	5	5	4	3
5	3	3	1	3	1	5	3	5	3
1	3	2	5	1	1	1	3	5	5

4	5	5	5	5	4	5	5	5	5
5	2	3	3	5	2	3	2	4	2
3	3	3	1	5	1	5	1	1	1
5	3	5	2	4	5	5	5	5	5
5	1	1	4	1	5	2	1	3	1
5	4	5	3	5	3	5	3	5	5
4	3	3	1	2	5	1	3	3	5
5	5	5	5	5	5	5	5	5	5
1	3	1	5	1	1	3	5	5	5
5	5	5	5	5	5	5	5	5	5
5	3	4	5	3	5	5	4	5	5
5	5	5	5	5	5	5	5	5	5
5	4	2	4	3	2	5	3	2	3
1	2	2	3	1	2	3	2	4	1
4	4	5	3	5	2	4	4	4	4
4	3	4	3	4	3	4	4	5	5
5	4	3	3	5	5	5	5	5	2
5	5	5	3	5	5	5	5	4	3
5	3	2	5	4	5	5	3	3	3
5	5	3	3	5	5	5	5	5	5
5	3	2	1	5	1	5	5	5	5
5	5	3	5	5	2	5	5	5	2
5	1	1	3	3	3	5	1	1	2
5	5	4	5	5	5	5	5	5	5
5	5	5	3	5	5	4	5	5	5
5	2	3	3	5	5	5	2	5	3
3	2	3	3	1	1	5	3	3	3
4	4	4	3	4	4	4	4	4	4
5	5	5	5	5	1	5	5	5	5
1	3	3	3	3	3	3	3	5	3
5	5	3	5	5	5	5	5	4	3
3	2	3	5	2	4	3	3	5	3
5	4	5	4	4	4	5	5	5	5

EVASI_20	EVASI_21	EVASI_22	EVASI_23	EVASI_24	EVASI_25	EVASI_26	EVASI_27	EVASI_28	EVASI_29
5	5	5	5	5	5	5	3	3	1
5	5	3	5	5	3	3	3	3	1
5	4	3	5	2	4	5	4	3	5
5	5	5	5	3	3	5	5	5	1
5	4	4	5	2	3	4	2	4	3
5	5	5	5	5	5	5	5	5	5
3	2	2	3	3	3	3	3	3	3
2	1	1	1	3	1	1	1	1	1
5	5	4	5	5	5	4	5	5	3
5	5	5	5	5	5	5	5	3	1
1	2	1	4	1	3	1	5	1	1
5	5	3	5	4	4	3	5	3	5
5	2	1	3	3	1	1	3	1	5
4	4	5	5	3	3	4	4	3	3
3	5	4	3	4	5	3	5	3	3
5	5	5	4	4	4	5	5	4	3
3	2	2	3	4	2	3	2	2	2
5	5	4	5	5	5	5	4	5	3
5	4	5	5	3	4	5	5	5	3
5	4	4	5	4	5	5	4	4	3
5	5	5	5	3	5	5	5	5	5
5	5	5	5	5	3	5	3	5	3
5	3	3	5	3	3	5	3	3	5
5	5	5	5	5	3	5	3	5	5
5	5	3	5	5	4	5	4	5	5
5	4	5	4	4	4	5	4	5	5
5	5	5	5	5	5	5	5	5	4
5	1	1	1	4	2	2	3	1	5
3	5	3	5	3	5	3	5	3	1
5	4	4	5	4	4	5	4	4	4
5	4	4	5	4	4	5	4	4	4
5	4	3	4	5	4	5	3	4	5
4	4	4	4	3	4	4	4	3	4
5	5	5	5	5	5	5	5	5	5
2	3	2	2	3	3	2	3	3	1
3	5	3	5	3	5	5	5	3	2
3	2	2	1	2	2	5	5	3	5
5	5	5	5	5	5	5	5	5	5
5	5	5	5	4	5	5	5	5	5
5	5	3	5	5	5	5	5	3	5
3	2	2	3	3	3	3	3	2	3
5	5	5	5	5	5	5	5	5	5
5	3	2	5	3	2	5	3	3	5
5	5	5	5	4	5	5	5	5	5
5	5	5	5	5	5	5	5	5	1
5	5	5	5	3	3	5	5	5	5

3	2	1	3	3	3	3	5	2	3
4	4	4	4	5	4	4	4	4	4
5	5	5	3	5	5	5	5	5	3
5	5	5	5	5	5	5	5	5	4
5	4	5	5	5	4	5	4	4	5
5	5	4	4	5	5	5	5	5	4
4	2	4	4	5	5	3	2	2	5
1	1	1	2	1	2	1	1	1	1
4	4	4	5	3	4	4	5	4	4
5	5	5	5	5	5	5	5	5	5
5	3	3	5	3	3	5	3	3	1
5	5	5	4	4	4	4	5	5	5
3	5	5	5	5	5	5	5	5	5
5	5	4	5	5	5	5	5	5	5
5	5	5	5	5	5	5	5	2	5
5	5	5	5	5	5	5	5	2	5
5	4	4	5	5	4	5	5	5	4
3	3	3	3	3	3	3	2	3	2
3	3	2	3	3	2	3	3	3	2
5	5	5	5	4	5	5	5	5	4
5	5	5	5	5	5	5	5	4	5
5	3	3	3	3	3	3	3	3	5
5	3	1	5	4	1	5	3	5	5
5	5	5	5	5	5	5	5	5	3
4	4	4	4	4	4	4	4	4	2
5	1	1	3	5	1	1	1	1	5
4	4	4	4	4	4	4	4	4	5
5	5	5	5	5	5	5	5	3	5
5	5	5	5	4	5	5	5	5	3
5	3	4	5	3	3	3	3	3	2
3	3	3	4	4	5	3	5	3	3
5	5	5	5	5	5	5	5	5	4
5	5	5	4	5	5	5	5	5	5
5	4	3	5	4	3	3	4	3	3
2	5	2	5	2	5	3	5	3	3
5	1	3	5	3	2	5	2	5	4
5	4	3	5	5	4	5	4	3	4
5	4	2	3	5	3	5	5	2	3
3	2	3	4	2	3	1	3	1	1
5	5	5	5	5	5	5	5	5	5
5	1	3	1	5	1	3	3	3	5
5	5	5	5	5	5	5	5	5	3
3	3	3	5	3	3	5	2	4	3
5	5	5	5	5	5	5	5	3	5
5	1	5	5	5	5	5	5	3	5
5	5	5	5	5	5	5	5	5	5
5	5	5	5	5	5	5	4	5	5

3	3	5	5	3	5	3	5	3	5
5	5	4	5	3	4	5	5	5	4
5	1	3	1	1	1	5	1	4	5
5	5	5	5	5	5	5	5	5	5
5	3	2	5	1	2	5	2	3	1
5	1	3	5	3	1	5	1	3	5
3	5	3	5	5	5	5	3	3	5
5	3	3	3	5	3	3	3	3	4
5	5	5	5	5	5	5	5	5	5
5	1	1	4	2	4	5	5	4	3
5	5	5	5	4	4	5	5	4	5
5	2	3	4	4	4	5	4	3	4
5	5	5	5	5	5	5	5	5	5
4	4	4	4	5	4	4	4	4	4
5	5	5	5	5	5	5	5	5	5
5	5	5	3	4	5	3	3	3	5
5	5	5	5	2	2	5	5	3	5
5	5	5	5	2	5	5	5	5	4
2	2	2	2	1	2	2	4	2	2
5	5	5	5	5	5	5	5	5	5
4	3	4	2	5	2	5	2	3	5
3	3	5	5	2	3	5	5	5	3
5	2	2	5	5	3	5	3	2	1
5	2	1	2	5	3	1	5	1	5
5	5	4	5	5	5	5	4	5	4
3	4	5	3	5	4	5	4	3	1
5	1	3	3	5	1	5	1	2	2
5	5	2	4	2	5	5	5	2	5
4	3	3	4	4	3	4	3	3	3
5	4	3	1	2	1	5	1	3	1
5	5	5	5	5	5	5	5	5	4
5	5	5	5	5	5	5	5	5	5
5	5	5	5	5	5	5	5	5	5
3	3	3	3	3	3	5	5	3	5
5	5	5	5	3	5	5	5	5	3
5	5	5	5	3	5	5	5	5	3
5	5	5	5	5	5	5	5	5	5
5	3	3	5	3	5	4	5	4	5
5	5	5	5	5	5	5	5	5	5
5	4	4	4	4	3	5	4	4	5
5	2	2	4	3	3	4	3	2	5
5	2	4	5	5	5	5	5	5	5
5	5	5	5	5	5	4	5	4	3
1	1	1	1	2	1	2	1	1	1
5	5	5	5	5	5	5	5	5	5
5	5	5	5	5	5	5	5	5	4
1	3	1	5	5	2	1	5	1	5

5	5	5	5	5	5	5	5	5	5
5	3	1	5	3	3	5	5	5	4
5	2	5	5	1	1	5	1	5	5
1	1	1	5	1	5	1	5	1	1
2	2	1	3	2	1	3	2	2	2
4	5	5	5	5	5	5	5	4	4
5	1	1	2	1	1	1	1	1	1
5	3	5	5	5	3	5	5	4	4
3	3	3	3	3	3	3	4	3	3
5	5	5	5	5	5	5	5	5	5
3	5	5	5	5	5	5	5	5	1
5	1	1	3	1	1	1	1	1	4
5	5	5	5	4	5	5	5	5	5
5	5	5	5	5	5	4	3	5	2
5	4	4	4	3	3	4	4	4	3
5	5	5	5	5	5	5	5	5	3
5	5	5	5	5	5	5	5	5	5
5	2	1	2	5	2	2	2	2	5
5	5	5	5	5	5	5	5	5	5
5	5	5	5	5	5	5	5	5	5
5	3	3	5	3	5	3	4	2	5
1	5	4	5	5	5	5	5	4	1
4	4	4	5	4	4	5	5	4	4
5	5	4	5	5	4	5	5	5	5
4	2	3	3	3	2	5	3	2	5
5	5	5	5	5	5	5	5	5	5
5	5	5	5	5	5	5	5	5	5
5	5	4	4	4	4	4	5	4	4
5	2	2	4	3	1	5	1	3	5
2	1	1	5	5	3	1	4	1	1
5	5	3	5	3	5	5	5	3	5
2	3	2	3	4	3	2	3	2	3
5	5	5	5	5	5	5	5	5	5
3	3	3	3	5	3	3	3	3	1
2	1	1	3	2	3	1	1	1	1
5	5	5	5	5	5	5	5	5	5
5	4	4	4	3	5	4	5	5	5
5	5	4	5	3	5	5	5	5	3
5	5	5	5	5	5	5	5	5	5
5	5	5	5	5	5	5	5	5	4
5	5	5	5	5	5	5	5	5	5
5	5	3	5	5	5	5	3	3	5
5	4	5	5	5	5	5	5	5	4
5	5	5	5	5	5	5	5	5	5
5	4	3	4	4	4	4	5	4	4
5	5	5	5	5	5	5	5	5	1
5	2	1	2	4	2	5	3	1	5

2	2	1	1	2	2	2	2	2	2
5	3	2	3	5	5	3	2	2	1
3	5	5	5	3	5	5	5	3	3
5	5	4	5	4	3	4	2	4	5
3	3	3	5	5	5	5	5	2	1
5	5	3	5	5	5	5	5	3	1
5	3	3	3	5	3	3	3	3	1
5	5	5	5	5	5	5	5	5	3
5	5	5	5	5	5	5	5	5	5
5	5	5	5	5	5	5	5	5	5
3	4	3	5	5	4	3	5	3	2
2	2	2	2	3	3	3	1	1	2
5	4	5	5	5	3	5	5	5	3
4	5	5	5	4	5	5	5	4	3
5	5	5	5	3	5	5	5	5	5
5	1	1	5	5	1	5	1	3	5
2	3	2	3	4	3	3	3	3	1
5	5	5	4	5	5	5	5	5	5
2	2	2	2	3	2	1	1	1	1
5	4	5	5	3	2	5	5	5	4
4	4	4	4	4	3	4	3	4	4
4	4	4	4	3	4	4	4	4	4
5	3	3	4	5	4	4	3	3	3
5	5	3	5	4	3	5	3	3	4
5	5	5	5	5	5	5	5	5	5
5	4	4	5	4	5	5	5	5	4
5	4	4	5	4	5	4	4	5	4
5	5	4	5	5	5	5	5	5	5
2	3	2	3	3	2	2	4	2	1
4	4	3	4	3	4	3	4	3	3
5	5	5	5	5	5	5	5	5	5
4	4	4	4	3	4	3	3	3	3
4	2	3	2	3	2	4	3	3	5
4	2	2	2	5	2	2	2	2	5
5	5	5	5	5	4	5	4	4	4
5	5	5	5	5	5	5	4	5	4
5	4	5	4	5	4	5	4	5	5
1	1	3	5	4	1	3	1	1	1
1	1	1	4	5	3	1	3	1	5
5	4	4	5	4	4	5	4	4	3
5	5	5	5	3	5	5	5	5	4
4	2	2	4	3	4	4	2	2	4
5	1	3	3	5	1	5	1	3	3
4	4	3	4	5	3	4	4	3	5
4	4	4	4	4	4	4	4	4	4
5	5	5	5	5	5	5	5	5	5
3	3	3	5	3	3	4	5	3	1

4	3	3	3	3	3	5	5	3	3
2	1	1	2	3	4	1	4	1	1
5	5	4	4	3	4	5	5	5	5
3	3	4	3	3	3	3	2	3	2
5	5	5	5	5	5	5	4	5	5
1	1	1	5	5	1	1	1	1	5
5	4	4	5	5	4	4	5	4	5
2	2	4	4	3	3	4	3	4	1
5	5	4	5	5	5	5	5	4	4
5	4	5	5	3	5	5	5	5	4
5	5	4	4	3	5	4	5	3	3
1	3	1	3	5	4	1	5	1	5
5	3	3	5	3	3	5	2	5	5
3	3	2	3	3	3	3	3	2	2
5	5	5	5	3	4	5	3	5	3
5	5	5	5	4	5	5	5	5	4
4	4	3	3	4	4	3	4	3	3
3	1	1	1	3	1	1	1	2	3
4	5	4	5	4	4	4	4	4	1
5	5	5	5	4	5	5	5	4	1
5	5	4	5	3	5	5	5	4	4
5	5	5	5	3	5	5	5	4	2
5	5	4	4	5	5	4	5	5	4
5	5	5	5	5	5	5	5	5	4
5	5	5	5	5	5	5	5	5	3
5	5	5	5	5	5	5	5	5	5
4	5	5	5	5	5	5	5	5	2
5	3	3	3	3	3	4	5	3	5
5	2	1	5	2	1	1	2	2	5
4	3	3	4	4	2	4	4	3	5
3	4	3	5	5	5	5	3	3	1
2	2	2	2	3	2	2	2	2	2
5	5	4	5	4	5	5	5	5	5
1	3	3	2	5	1	4	2	2	1
5	5	4	5	4	3	5	4	4	5
5	5	5	5	5	5	5	5	5	3
4	2	3	5	5	2	5	3	3	5
4	4	4	4	2	4	4	4	4	3
3	4	3	3	3	2	3	3	3	2
5	5	5	5	5	5	5	5	5	5
5	3	4	5	3	3	5	3	5	5
5	4	4	5	3	5	5	5	4	5
5	1	1	5	4	1	5	4	5	5
5	4	5	5	3	5	5	4	3	5
1	1	1	1	2	2	1	2	2	1
4	5	4	4	5	5	5	5	5	4
5	5	5	5	4	4	4	5	5	3

5	3	3	5	5	2	5	5	3	5
4	3	3	5	3	3	4	3	3	4
5	5	4	5	4	5	4	4	4	4
3	3	5	5	3	4	5	5	4	1
5	5	5	5	5	5	5	5	5	5
5	3	1	5	5	1	1	5	1	2
5	5	5	5	4	5	5	5	5	1
5	5	4	4	4	5	5	5	4	4
5	2	2	5	5	2	3	3	2	3
4	3	3	4	5	3	5	3	4	3
3	3	3	3	3	3	3	4	3	3
5	5	5	5	5	5	5	5	5	5
4	3	3	4	3	3	4	4	3	4
5	5	5	4	5	5	5	4	5	4
5	5	5	5	5	5	5	5	5	1
5	5	5	5	3	5	5	5	5	3
4	2	2	4	4	4	4	3	2	2
4	1	4	4	3	5	5	5	3	5
5	5	5	5	5	5	5	5	5	5
5	3	3	5	5	2	5	3	3	5
5	5	5	5	5	5	3	5	3	3
5	5	5	5	5	5	5	5	5	5
5	5	5	5	5	5	5	5	5	5
4	5	5	5	2	5	5	4	3	4
5	1	1	1	5	1	1	1	1	5
5	5	5	5	3	4	5	5	5	4
4	5	3	5	4	5	3	5	3	3
4	5	3	5	4	5	3	5	3	3
5	5	5	5	5	5	5	5	5	5
1	1	2	2	4	1	2	1	2	1
5	4	5	3	4	3	5	5	5	4
5	5	5	5	5	5	5	5	5	5
5	5	5	5	5	5	5	5	5	5
4	5	5	5	3	5	4	5	4	4
5	5	5	4	5	4	5	4	4	5
2	2	3	3	3	1	2	3	3	3
5	2	1	2	2	1	3	5	2	5
5	5	5	5	3	5	5	5	5	5
5	3	4	3	5	3	5	4	3	5
5	4	4	5	3	5	5	5	5	5
5	4	3	4	2	3	4	4	3	5
4	5	5	5	5	4	4	5	4	2
5	5	5	5	5	5	5	5	5	4
5	5	5	5	5	5	5	5	5	5
4	2	2	4	3	4	5	5	5	5
5	5	5	4	5	5	5	5	5	5
5	1	1	2	5	2	2	3	1	5

4	4	4	4	4	4	4	4	4	3
5	5	5	5	5	5	5	5	5	4
4	4	4	4	5	4	4	3	4	3
5	5	5	5	5	5	5	5	5	5
5	5	5	5	5	5	5	3	5	5
5	5	5	5	5	5	5	5	5	5
5	5	5	5	5	5	5	5	5	5
4	2	2	3	3	2	3	2	2	3
3	3	3	3	3	3	2	4	2	1
5	3	3	5	4	3	5	3	3	3
5	5	5	5	3	5	5	5	5	5
1	1	1	1	3	1	1	1	1	1
5	5	5	5	3	5	5	5	5	3
5	3	3	3	5	3	5	3	4	5
5	5	4	5	5	5	5	5	5	5
5	5	2	2	2	1	5	2	2	5
5	1	5	5	5	5	5	5	5	5
5	5	5	5	5	5	5	5	5	5
5	5	5	5	2	5	5	5	4	3
5	1	5	5	5	5	5	5	5	5
3	5	3	5	3	3	3	3	3	3
5	3	5	5	3	5	5	3	5	2
5	5	5	5	5	5	5	5	5	5
5	3	3	3	3	3	5	5	3	5
5	5	5	5	4	5	5	4	5	5
2	2	2	2	3	2	3	5	3	3
5	4	3	4	4	4	4	4	4	4
5	4	4	5	4	4	4	5	5	4
4	3	3	5	5	5	5	5	5	1
4	4	4	4	3	4	3	3	3	3
5	5	5	5	5	5	5	5	5	4
3	5	5	5	3	5	5	5	4	3
4	4	5	4	5	5	5	5	5	5
5	5	4	5	5	5	5	5	5	5
3	3	4	5	5	5	5	5	5	3
5	4	2	5	5	5	5	4	2	4
5	5	5	5	5	5	5	5	5	5
5	5	4	5	4	5	4	4	4	5
5	3	4	4	3	4	4	4	4	3
3	4	4	5	3	5	5	5	4	3
5	1	5	5	5	5	5	4	4	4
5	5	5	5	5	5	5	5	5	5
5	4	4	5	2	3	3	3	3	5
5	5	5	5	5	5	5	5	5	5
5	5	5	5	5	5	5	5	5	5
5	5	5	3	3	3	5	3	5	3
5	5	5	5	4	5	5	5	5	4

4	4	4	4	4	4	4	4	4	5
5	5	4	5	3	5	5	5	3	4
5	3	4	5	5	5	5	5	5	5
5	5	5	5	3	5	5	5	5	5
5	5	3	5	3	5	5	5	5	5
5	5	5	5	3	4	5	5	5	5
4	4	4	5	3	4	4	4	3	2
4	1	1	3	3	2	2	3	1	5
5	3	2	5	5	3	3	3	2	3
5	5	4	4	5	4	4	4	5	1
5	4	4	4	5	5	5	4	5	4
5	5	5	5	5	5	5	5	5	1
4	5	4	4	4	5	4	5	3	3
4	4	3	4	2	3	4	3	3	3
5	5	4	5	4	5	5	5	5	5
5	5	5	5	5	5	5	5	5	5
5	3	3	3	2	3	3	3	3	3
5	5	5	5	3	5	5	5	5	5
3	1	1	1	3	5	2	5	1	2
3	5	5	3	3	5	3	5	3	3
5	5	5	5	3	5	5	5	5	4
5	3	2	2	3	3	1	5	2	3
5	4	4	5	3	3	5	5	4	1
3	3	3	3	5	3	3	3	3	3
5	3	1	5	2	5	1	5	1	2
5	5	5	5	5	5	5	5	5	5
5	5	5	5	3	5	5	5	5	3
4	4	4	5	5	5	4	5	4	3
5	2	3	5	5	2	1	5	5	5
5	5	5	5	5	5	5	5	5	4
5	5	5	5	4	5	5	5	5	5
5	5	5	5	5	5	5	5	5	3
5	4	4	4	4	4	4	4	4	4
5	5	5	5	5	5	5	5	5	5
5	2	2	2	1	2	5	3	5	3
5	5	4	5	3	5	5	5	5	5
5	5	5	5	3	5	5	5	5	4
2	2	2	2	1	2	3	2	2	4
5	5	5	4	5	5	5	5	5	5
4	3	3	3	4	3	4	3	3	4
3	4	3	5	3	4	3	5	3	3
5	5	4	4	5	4	5	5	4	5
5	5	3	4	2	3	3	3	3	5
4	5	4	4	1	5	5	5	4	4
5	3	3	4	3	3	4	2	3	4
1	1	2	2	5	4	2	3	2	1
3	1	1	2	1	2	3	3	3	3

5	1	2	1	5	1	2	1	1	5
5	5	5	5	5	5	5	5	5	5
5	1	2	3	5	2	3	2	2	5
5	3	3	3	2	3	5	2	5	5
5	5	5	5	4	5	5	5	5	3
5	3	2	3	3	3	2	3	3	5
5	5	2	5	1	2	5	3	4	5
2	2	1	2	1	1	2	1	2	1
4	4	4	4	4	4	4	4	4	3
5	5	5	5	5	5	5	5	5	1
5	3	3	4	2	4	4	4	5	5
5	5	5	5	5	5	5	5	5	1
5	5	1	3	3	1	5	1	2	5
4	4	4	5	5	5	4	5	4	4
5	5	5	5	5	5	5	5	5	5
4	4	4	5	3	5	3	5	4	3
5	4	3	5	2	5	5	5	5	5
3	2	2	2	2	2	2	3	2	2
2	5	2	2	5	5	3	3	2	5
4	4	5	5	3	5	4	5	4	1
5	5	5	5	5	5	5	5	4	4
3	5	5	5	5	5	5	5	3	3
4	3	4	4	3	4	4	5	3	2
5	4	4	4	4	5	5	5	5	4
1	5	1	3	1	3	1	5	1	3
5	5	4	4	5	5	5	4	5	3
5	5	5	5	5	5	5	5	5	5
5	5	5	5	5	5	5	5	5	5
5	5	5	5	5	5	5	5	5	5
5	5	5	5	5	5	5	5	5	5
5	4	5	5	3	5	5	5	5	5
5	2	1	3	5	3	5	5	5	5
3	4	3	5	3	5	5	5	5	5
5	3	3	5	5	3	5	3	3	5
5	5	5	5	5	5	5	5	5	5
5	5	5	5	5	5	5	5	5	3
4	5	4	4	5	5	4	5	4	5
5	5	5	4	3	5	5	5	5	5
5	4	4	5	5	4	5	5	4	4
5	5	5	5	5	5	5	4	5	5
5	4	4	4	4	4	4	5	5	3
5	5	3	5	3	5	4	5	3	5
5	5	5	5	5	5	5	5	5	5
5	5	5	5	5	5	5	5	5	5
4	4	5	5	3	5	5	5	3	4
3	4	4	4	2	4	4	4	4	4
5	5	5	5	5	5	5	3	5	3

3	3	3	3	2	3	3	4	3	3
3	3	2	2	2	2	3	2	2	4
5	5	5	5	5	5	5	5	5	4
5	3	3	5	4	3	5	3	4	5
5	1	1	1	5	1	1	1	1	1
5	2	4	4	1	3	4	5	2	3
5	5	5	5	4	5	5	5	5	4
4	4	3	4	2	3	4	4	3	4
5	4	3	5	3	4	5	4	5	5
5	5	5	5	3	5	5	5	3	5
5	4	4	5	2	5	5	5	4	4
5	5	5	5	4	5	5	5	5	4
5	5	5	5	5	5	5	5	5	5
5	5	5	5	4	5	5	5	5	3
5	5	3	4	2	3	5	5	5	3
4	4	4	4	3	4	4	4	4	3
3	4	3	3	4	4	2	4	2	3
5	4	4	5	5	4	4	3	4	3
5	3	3	5	5	3	5	5	3	3
5	5	5	5	5	5	5	5	5	5
5	5	5	5	5	5	5	5	5	5
5	1	2	5	5	2	5	2	2	5
5	4	5	4	5	5	4	5	3	5
5	3	5	5	5	5	5	5	5	5
5	2	5	5	5	5	5	5	5	5
5	5	5	5	3	5	5	5	5	5
5	5	5	5	3	5	5	5	4	2
5	3	2	4	4	2	4	3	4	5
3	3	2	4	2	3	3	3	3	3
5	3	3	4	1	3	5	3	3	4
5	3	3	3	5	2	4	5	4	5
5	2	1	5	5	1	1	4	1	2
1	1	3	2	3	3	1	2	1	1
4	5	4	4	3	4	4	5	4	4
5	5	5	5	5	5	5	5	5	2
5	2	5	2	5	5	5	5	5	5
5	5	4	4	3	3	5	5	3	5
5	3	5	5	5	5	5	5	5	3
5	1	4	5	5	5	5	4	4	5
3	4	4	4	3	5	4	5	5	3
5	4	4	4	5	5	5	5	4	4
5	2	2	4	4	2	5	2	2	5
5	3	4	5	5	5	5	5	3	4
5	5	5	5	5	5	5	5	5	5
5	4	4	5	4	5	4	4	4	3
5	5	5	5	5	5	5	4	4	5
1	1	1	1	2	1	2	1	2	2

5	3	4	3	3	2	4	3	4	4
5	5	5	5	4	5	5	5	5	5
5	5	5	5	5	5	5	5	5	4
5	5	5	5	3	5	5	5	4	3
5	4	4	4	3	4	5	4	5	5
3	3	3	4	3	4	3	4	4	3
3	2	3	3	2	3	2	3	3	3
5	3	4	5	5	3	5	3	4	5
3	3	1	3	3	3	1	3	1	1
5	4	5	5	3	3	5	4	5	3
5	4	4	5	5	5	5	5	5	5
5	5	5	4	5	4	5	4	5	1
5	5	5	5	5	5	5	5	5	5
5	2	3	5	3	3	4	5	3	5
5	5	5	5	5	5	5	5	5	5
5	5	5	5	5	5	5	5	5	5
5	5	5	5	5	5	5	5	5	4
3	2	2	4	2	5	3	3	2	2
5	5	4	5	3	5	5	5	4	5
5	5	4	5	5	5	5	5	4	4
5	1	5	5	5	5	5	5	5	1
5	5	5	5	3	4	5	5	5	5
5	5	5	5	4	4	5	5	5	4
5	5	4	5	2	5	5	5	5	4
5	4	3	3	4	3	5	4	4	5
4	3	4	4	5	4	5	4	4	5
4	3	3	3	1	2	4	3	3	3
5	3	3	5	5	3	5	3	4	5
5	5	5	5	4	5	5	5	4	5
5	4	5	3	5	3	5	3	5	5
5	5	4	5	4	4	4	5	5	5
4	4	3	4	3	3	5	4	5	4
5	5	5	4	5	4	5	5	5	3
5	5	5	5	5	5	5	5	5	4
5	5	5	5	5	5	5	5	5	5
4	4	4	4	4	4	5	4	4	4
4	4	4	3	5	5	5	3	3	3
5	4	4	5	3	5	5	5	3	5
5	3	3	3	5	2	5	2	5	3
5	5	5	5	5	5	5	5	5	4
5	5	5	5	3	5	5	5	5	4
4	4	4	5	3	4	4	5	5	5
5	2	3	3	5	2	5	2	2	5
5	5	5	5	5	5	5	5	5	5
3	2	4	4	5	3	3	4	4	1
5	5	3	3	5	3	3	5	3	3
1	5	1	5	4	5	1	5	1	1

5	5	5	4	4	5	5	5	5	4
5	2	2	3	4	3	4	3	2	3
3	2	2	3	3	1	1	1	1	2
5	4	4	5	2	5	5	5	3	3
2	1	1	1	3	1	1	1	1	5
5	4	3	5	4	5	5	5	5	5
3	2	3	5	1	3	3	5	3	3
5	5	5	5	5	5	5	5	5	5
1	5	5	5	5	5	1	5	5	5
5	5	5	5	5	5	5	5	5	5
5	5	3	5	5	5	5	5	5	5
5	5	5	5	5	5	5	5	5	5
4	2	3	3	3	4	4	4	3	5
1	1	1	3	1	2	1	3	1	1
5	3	4	4	3	3	4	4	5	2
4	4	4	4	5	4	4	5	4	5
4	4	5	5	4	2	5	3	5	3
5	5	5	5	3	3	5	3	5	4
4	3	3	3	4	3	5	4	3	4
5	5	5	5	5	5	5	5	5	3
5	3	3	5	5	5	5	5	5	5
5	5	5	5	3	2	5	5	5	3
4	3	3	5	2	1	4	3	2	5
5	5	5	5	4	5	5	5	5	5
5	5	5	5	3	5	5	5	5	3
5	3	2	3	5	2	5	3	3	5
3	3	3	3	3	3	1	3	1	5
4	4	4	4	3	4	3	4	3	3
5	5	5	5	5	5	5	5	5	5
1	3	3	5	3	3	3	5	3	1
5	5	5	5	4	2	5	5	4	4
2	2	3	3	4	3	1	1	1	3
5	5	4	5	4	5	5	5	5	5

EVASI_30	EVASI_31	EVASI_32	EVASI_33	EVASI_34	EVASI_35	EVASI_36	EVASI_37	EVASI_38	GDS_1
1	3	1	3	5	2	3	1	2	1
1	3	3	5	5	2	3	3	3	0
4	5	3	5	2	3	2	1	2	1
1	5	5	3	1	3	1	1	3	0
4	4	2	5	4	4	4	1	5	1
1	5	1	5	5	5	1	1	5	0
4	3	5	3	2	2	3	3	2	1
2	1	3	3	1	1	3	3	1	1
3	5	1	1	1	5	1	1	5	0
3	5	3	5	3	3	1	3	2	0
2	1	4	2	5	1	5	1	2	0
3	5	3	5	5	2	1	1	2	0
3	5	3	4	1	1	3	1	1	0
2	4	2	5	4	3	3	3	4	0
3	3	3	3	5	4	3	3	5	0
3	4	2	5	5	4	2	1	4	0
2	3	3	4	5	2	2	4	2	1
2	5	3	3	2	5	2	1	4	0
3	5	2	3	4	4	1	1	3	0
3	5	3	5	5	4	2	1	4	0
1	5	1	5	3	3	1	1	4	0
3	5	3	5	1	3	1	3	3	0
3	5	3	3	3	3	1	2	3	0
3	5	3	5	5	3	1	5	3	0
5	5	3	5	5	3	1	3	3	0
2	5	2	4	4	4	2	2	5	0
4	5	3	4	4	4	3	3	5	0
5	5	3	5	4	1	1	1	1	1
2	2	3	3	5	2	4	1	2	0
4	5	3	4	4	4	2	2	4	0
4	5	3	4	4	4	2	2	4	0
2	5	1	4	5	3	1	1	1	0
3	4	3	4	4	4	3	3	3	0
2	5	2	5	5	5	1	1	5	1
3	3	4	3	3	3	3	2	3	0
3	3	3	5	5	3	3	1	3	0
5	5	1	5	5	1	5	4	5	1
3	5	3	5	5	5	1	1	5	0
3	5	2	5	5	4	1	5	5	0
5	5	5	5	5	2	5	1	2	0
3	3	5	3	3	2	3	3	3	0
2	5	1	5	5	5	1	1	5	0
5	4	4	4	5	2	1	2	1	0
1	5	1	5	5	5	1	1	5	0
4	5	5	5	5	3	1	1	5	0
3	5	3	3	3	2	3	2	2	0

5	4	3	3	1	2	4	4	1	0
1	4	3	5	2	4	2	2	3	0
3	5	3	3	5	3	3	3	3	0
1	5	1	5	5	5	1	1	5	0
5	5	1	5	5	5	1	1	5	1
5	5	1	4	5	4	2	1	5	0
4	2	4	4	3	5	5	5	4	0
1	1	3	1	1	1	1	5	5	0
5	4	5	5	1	5	4	1	5	0
1	5	1	5	5	5	1	2	5	0
4	3	4	3	2	3	3	2	2	0
4	4	3	3	4	4	4	1	4	0
5	5	5	5	4	5	3	3	5	0
3	3	5	5	5	5	2	5	3	0
3	5	3	5	5	5	1	3	5	0
3	5	3	5	5	5	1	3	5	0
4	5	5	4	4	3	3	1	4	0
2	3	3	3	3	3	4	3	3	0
2	3	5	5	3	3	3	3	3	1
3	5	3	5	4	5	3	3	5	0
3	5	3	5	5	3	1	2	2	0
5	5	3	5	5	3	1	1	2	1
1	5	2	5	5	1	1	3	1	0
3	5	3	5	5	5	3	1	4	0
1	4	1	4	5	3	1	1	2	0
1	5	1	5	5	1	5	5	1	1
2	5	2	4	2	4	1	1	5	0
4	3	5	5	5	5	3	3	5	1
3	5	3	4	5	4	3	1	5	1
4	3	3	4	2	2	4	4	3	1
4	3	5	3	3	3	4	3	4	0
2	5	2	4	5	4	1	1	2	0
1	5	1	5	5	5	1	1	4	0
4	4	3	4	4	3	3	2	4	0
1	3	3	3	1	3	3	5	2	0
3	5	3	4	5	3	2	4	3	1
3	5	3	5	5	3	3	1	3	0
1	5	3	5	5	3	1	1	3	0
5	1	5	2	2	1	4	2	1	1
4	5	2	5	5	3	1	3	4	0
5	5	3	5	5	1	1	1	1	1
3	5	1	5	3	5	1	3	5	0
3	5	4	3	5	3	2	3	4	1
5	5	1	5	5	5	5	5	5	0
3	5	1	5	1	4	1	1	5	0
3	5	1	5	5	4	2	4	5	0
2	5	1	4	5	4	1	1	5	0

3	4	3	5	3	3	5	3	4	0
1	5	1	5	1	5	1	1	2	0
5	5	3	5	5	1	3	4	2	0
2	5	2	5	5	5	1	1	5	0
4	5	3	3	3	1	3	4	2	0
3	3	1	5	5	2	1	1	1	0
5	3	5	2	1	1	1	5	3	1
1	5	2	3	1	2	1	4	2	1
3	5	2	5	5	5	1	1	5	0
3	4	3	5	5	2	1	3	4	0
2	5	3	5	4	4	1	2	4	0
2	5	2	4	4	4	2	3	4	0
1	5	1	5	5	5	1	1	5	0
3	4	1	3	5	4	1	1	5	0
1	5	1	5	5	5	1	1	5	0
1	5	3	5	5	3	1	2	2	1
5	5	4	5	5	1	1	2	1	0
3	5	2	5	3	5	1	1	5	0
5	2	5	5	2	2	4	3	2	1
1	5	1	5	5	5	1	1	5	0
3	5	3	5	5	3	1	3	5	0
3	5	1	3	3	5	3	3	2	0
5	3	5	5	5	5	3	4	2	1
4	5	5	5	5	1	1	5	1	0
3	4	3	4	5	3	1	1	3	0
1	3	1	4	5	4	3	1	3	0
3	5	5	5	1	2	1	1	2	1
4	4	4	5	3	2	1	3	4	0
3	4	3	3	2	3	2	2	2	0
5	3	1	5	5	1	3	3	1	0
1	5	2	5	3	5	1	2	5	0
3	5	3	5	5	5	3	1	5	0
5	5	3	5	5	5	3	3	5	1
3	5	3	3	3	3	3	3	3	0
3	5	3	3	5	5	2	2	5	0
2	5	1	5	5	5	1	1	5	0
3	5	5	5	5	3	3	1	5	0
3	5	5	4	4	4	3	5	4	0
5	5	3	4	3	5	1	1	5	0
5	5	3	5	5	2	2	1	3	1
5	5	5	5	5	2	3	1	1	0
4	3	3	2	1	3	1	1	3	0
2	5	1	3	4	5	1	1	3	0
2	1	3	1	2	1	4	2	3	1
3	5	2	5	5	5	1	1	5	0
1	5	1	5	5	5	1	1	5	0
5	5	3	5	5	1	3	3	1	0

1	5	1	5	2	4	1	1	4	0
5	5	4	5	4	2	2	3	1	0
5	5	1	5	1	1	3	1	3	1
5	1	5	2	1	1	5	1	2	0
3	3	3	3	1	1	3	3	2	1
3	4	3	3	4	4	3	3	5	0
3	5	2	5	5	1	1	1	1	0
5	5	4	5	4	5	2	4	5	1
4	4	4	4	4	3	3	1	3	0
2	5	3	5	5	5	1	1	5	0
5	4	5	5	1	5	1	1	5	1
1	4	1	4	4	1	2	5	1	1
4	5	4	5	4	4	2	3	3	0
3	5	3	5	3	5	3	3	5	0
3	4	3	5	5	5	1	3	5	0
1	4	3	3	2	3	3	1	5	1
1	5	1	5	1	5	1	1	5	0
1	5	1	5	5	3	1	3	3	1
1	5	3	5	5	5	1	1	5	0
3	5	3	5	5	5	3	1	4	0
5	5	4	5	1	2	1	1	3	1
1	5	2	3	5	3	1	1	4	0
3	4	4	4	4	4	3	3	4	0
3	5	4	5	5	5	1	3	4	0
5	5	1	5	3	2	1	3	2	0
5	5	3	5	5	5	1	1	5	0
5	5	4	5	5	4	1	3	5	0
3	4	3	4	4	4	3	1	3	0
1	5	1	5	5	1	1	2	2	1
3	3	1	4	5	1	5	1	1	0
4	5	3	5	5	3	3	2	5	0
4	3	4	5	2	2	3	1	2	0
3	5	3	5	5	5	1	1	5	0
3	3	5	2	1	3	4	4	2	0
1	1	3	1	1	1	5	1	1	0
1	5	3	5	5	5	1	1	4	1
3	3	3	3	5	5	1	1	5	0
1	5	1	5	5	4	1	1	5	0
1	5	2	5	1	4	1	1	4	0
2	5	1	5	5	5	1	1	5	0
1	5	1	1	5	5	1	1	5	0
1	5	1	5	5	3	1	1	3	0
3	5	3	5	5	5	3	3	5	0
1	5	1	5	5	5	1	1	5	0
3	4	4	4	5	4	2	1	3	0
4	5	2	5	5	5	1	1	5	0
3	4	1	5	5	1	1	3	1	0

2	2	2	2	2	2	2	2	2	1
3	3	3	3	2	1	3	1	1	0
3	5	3	5	3	5	1	2	3	0
4	4	2	4	2	4	3	2	5	1
1	5	1	5	3	3	2	1	3	0
3	5	5	5	4	4	3	1	5	0
4	3	4	3	5	3	3	5	3	0
1	5	3	5	5	3	1	1	5	0
1	5	2	5	5	5	1	3	5	0
2	5	1	5	5	5	1	1	5	0
4	3	5	4	5	3	4	1	3	1
2	2	3	2	5	2	4	1	2	0
3	3	3	5	4	3	1	2	4	0
3	5	3	4	4	3	2	2	4	0
3	5	1	5	5	5	1	1	5	0
3	5	1	5	5	1	1	1	1	0
3	3	4	3	2	2	3	1	1	1
4	5	2	5	5	5	1	1	5	0
1	1	1	2	5	1	4	1	1	0
2	5	3	3	1	2	1	1	5	1
3	4	3	4	4	4	2	3	4	0
3	4	3	4	4	4	2	3	3	0
2	5	2	3	3	3	3	2	3	0
4	5	3	5	2	3	1	4	4	0
1	5	1	5	5	5	1	1	5	0
3	5	4	5	5	4	1	1	3	0
3	5	5	5	3	4	2	2	5	0
2	5	3	5	5	4	3	1	5	0
3	2	2	2	3	2	4	3	2	0
2	3	4	4	3	3	3	2	2	0
3	5	2	5	5	5	2	3	5	0
3	4	3	4	4	3	2	2	3	0
4	5	4	5	4	2	2	2	2	0
1	5	1	5	5	2	1	1	1	0
2	5	3	5	4	4	1	3	5	1
3	5	2	5	5	4	1	1	4	0
3	4	1	5	1	4	1	3	4	0
3	3	3	5	3	1	4	3	3	1
5	5	2	5	2	1	3	5	1	1
3	5	1	5	5	4	1	1	4	0
2	5	3	5	2	5	1	1	3	0
4	4	4	4	4	2	1	4	2	1
3	3	5	3	1	1	5	5	1	1
5	5	4	5	5	4	1	4	3	1
2	4	1	4	3	3	1	1	2	0
1	5	1	5	1	4	1	1	3	0
5	3	2	3	1	3	2	4	4	1

5	3	5	5	3	3	5	5	2	0
1	1	2	1	1	1	1	2	1	0
3	5	3	5	4	4	1	5	5	0
3	3	3	3	4	3	3	1	4	1
1	5	1	5	5	5	1	1	5	0
1	5	1	5	5	1	1	1	1	1
3	5	3	5	5	3	2	2	3	0
1	2	3	3	3	2	3	1	3	0
2	5	2	5	4	4	1	1	3	0
1	4	2	5	5	4	1	2	4	1
3	5	1	3	3	5	1	1	5	0
5	1	4	5	1	2	5	1	1	1
4	5	2	5	3	2	1	1	2	1
3	3	3	3	3	2	2	3	3	0
3	5	3	3	2	3	3	1	5	0
3	5	3	4	3	5	3	3	5	0
3	4	3	3	3	3	3	3	3	0
4	3	5	4	1	1	2	2	1	1
1	5	1	1	5	5	1	3	5	0
3	5	3	3	3	3	3	1	3	0
4	5	3	5	5	4	2	1	5	0
3	5	3	5	5	2	2	1	3	0
3	5	2	5	5	4	2	1	4	0
1	5	3	4	3	4	3	1	5	0
3	5	1	5	5	3	1	1	5	0
1	5	3	4	5	5	1	1	5	0
2	5	3	2	4	4	1	1	5	0
3	5	5	5	5	3	4	4	2	0
5	5	3	5	4	1	1	5	1	1
3	4	3	5	5	3	1	1	3	1
3	3	3	5	3	3	3	1	5	0
2	2	2	2	2	2	2	2	2	0
3	5	1	5	5	5	2	1	5	0
1	4	1	2	4	1	3	1	2	1
2	5	3	5	5	3	1	1	5	0
1	5	1	5	5	5	1	3	5	0
2	4	5	4	3	1	1	1	1	0
3	4	3	4	4	3	3	2	3	1
1	3	3	3	3	2	4	4	2	1
1	5	2	5	5	5	3	1	4	0
1	5	2	5	4	4	1	3	3	1
3	5	3	3	5	5	1	1	4	0
1	5	1	5	1	1	1	3	1	1
3	5	2	5	1	4	1	3	4	1
5	1	5	3	1	3	5	5	3	1
3	5	3	4	4	4	3	1	4	0
3	5	3	3	4	4	2	1	3	0

5	5	3	5	5	3	1	2	3	0
5	5	4	5	4	2	4	4	2	1
3	4	4	5	4	4	3	3	5	0
3	3	3	1	1	3	3	1	5	0
2	5	2	5	4	5	1	2	5	0
2	2	5	5	5	2	4	4	1	1
1	5	1	5	5	5	1	1	5	0
3	5	2	5	1	3	2	1	3	0
5	4	1	5	5	1	1	5	1	1
3	5	3	4	5	3	1	1	3	1
3	3	2	3	3	3	3	3	3	0
1	5	1	5	5	5	1	1	5	0
3	4	3	3	4	3	3	3	3	1
2	5	1	5	1	4	1	1	3	0
1	5	3	5	1	4	1	1	4	0
3	5	3	5	5	5	3	2	3	0
4	4	3	3	3	2	1	1	2	0
3	5	3	5	1	3	3	1	3	0
1	5	3	5	5	5	1	1	5	0
5	5	3	5	5	3	1	3	3	0
4	3	1	3	3	1	1	1	3	0
5	5	4	5	5	5	1	1	3	0
2	5	2	5	5	5	2	1	5	0
3	5	3	5	1	4	3	1	5	0
1	4	3	5	5	1	1	1	1	0
3	5	3	4	5	4	2	3	5	0
5	3	3	5	1	4	5	5	4	0
5	3	3	5	1	4	5	5	4	0
1	5	3	5	5	5	2	3	4	0
1	1	1	3	2	1	3	2	1	0
3	5	3	5	4	3	1	3	5	0
5	5	3	5	5	5	3	3	5	0
3	5	3	5	5	5	1	1	5	0
3	4	2	4	5	4	1	1	5	1
2	5	1	5	5	3	1	2	4	0
4	3	4	3	1	1	4	3	2	1
3	5	3	5	5	2	3	3	3	1
2	5	3	5	5	5	2	1	5	0
4	5	2	5	4	3	2	2	3	0
2	5	2	5	5	4	2	1	4	0
3	5	5	5	5	4	1	1	5	1
2	4	1	4	5	4	3	1	3	0
1	5	2	5	5	5	2	2	5	0
3	5	3	5	5	5	1	1	5	0
5	5	1	5	1	3	5	1	2	0
3	5	3	5	2	4	2	3	5	0
3	5	5	5	3	1	1	1	1	1

1	4	2	3	5	4	2	1	4	0
4	5	2	5	5	5	2	2	4	0
2	4	3	4	4	3	3	1	3	1
3	5	3	5	5	5	1	5	3	0
5	5	3	5	5	3	1	5	3	0
1	5	1	5	5	5	1	1	5	0
1	5	1	3	5	5	1	1	5	0
4	4	3	4	3	2	4	1	2	0
3	3	3	2	4	2	4	3	3	0
5	5	3	5	5	3	3	1	3	1
3	5	4	5	5	5	1	2	4	1
1	1	1	1	1	1	5	1	1	1
1	5	1	5	5	4	1	1	5	0
1	5	1	5	5	2	1	1	2	0
2	5	1	5	5	5	1	2	5	0
3	5	4	5	4	3	1	4	1	0
1	4	1	5	5	4	1	1	5	0
1	5	1	5	5	5	1	1	5	0
4	5	4	4	5	5	1	1	5	0
1	5	1	5	5	5	1	1	5	0
3	5	1	5	3	1	3	5	5	1
1	5	1	2	5	4	1	1	5	0
1	5	3	5	5	5	1	1	5	0
1	5	1	5	2	2	1	1	2	1
3	5	1	5	5	5	1	1	5	0
2	5	2	5	2	2	3	1	2	1
5	4	2	5	4	2	4	1	3	1
1	4	1	5	1	4	1	2	4	0
1	5	1	5	5	3	1	1	4	0
4	4	4	4	4	3	2	1	5	1
2	5	4	5	3	5	1	2	5	0
3	4	4	5	5	4	2	1	4	1
3	5	3	5	2	5	2	1	5	0
1	4	1	4	1	4	2	2	4	0
5	5	5	4	5	2	4	1	2	1
4	5	5	5	5	3	4	5	2	1
5	5	5	5	5	5	3	3	2	0
3	5	3	4	4	4	2	2	4	0
4	5	4	5	2	3	2	1	4	0
3	4	3	3	4	4	3	1	5	0
3	5	3	4	3	4	2	3	5	0
5	5	3	5	5	3	1	1	5	0
5	5	1	5	5	1	3	3	1	1
3	5	1	5	5	3	1	1	5	0
3	5	2	5	1	2	1	1	4	0
3	5	3	3	2	3	2	3	3	1
3	5	2	5	5	3	3	3	4	0

4	4	4	4	4	4	2	3	4	0
3	4	3	3	3	3	3	3	4	0
4	5	3	5	3	3	3	3	4	0
5	5	1	5	5	5	1	1	5	0
3	5	3	5	4	5	3	2	5	0
5	4	1	5	1	5	4	4	5	0
3	4	3	3	2	3	2	2	3	0
3	5	3	5	5	1	1	1	1	1
3	5	3	3	5	2	3	1	2	1
1	5	1	2	5	3	2	1	3	0
3	5	3	5	4	4	1	1	5	0
3	5	3	5	1	1	1	1	5	1
3	5	4	3	4	4	4	1	3	0
3	3	4	3	4	3	1	2	3	1
4	5	5	5	5	5	1	1	4	0
3	5	3	5	5	5	1	3	5	0
3	3	1	3	2	2	3	3	2	0
3	5	3	5	4	5	3	1	5	1
1	3	4	1	1	1	2	2	1	0
3	3	1	5	3	5	2	3	5	0
5	3	1	5	5	5	1	1	5	0
5	5	5	4	4	2	3	4	2	0
2	5	3	5	5	3	2	1	2	0
3	3	3	3	3	1	3	3	1	1
4	5	5	5	1	2	3	1	3	0
3	5	3	5	5	4	1	1	5	0
5	5	3	3	5	5	2	3	3	0
2	4	1	2	4	4	3	3	5	0
5	5	3	5	1	3	3	1	2	0
4	5	3	5	5	5	2	2	5	1
1	5	1	5	3	4	1	1	5	0
1	5	1	3	5	5	1	1	5	0
3	5	2	5	4	4	2	1	4	0
1	5	5	5	5	5	1	1	5	0
4	3	3	5	5	3	3	3	3	0
3	5	3	3	2	4	2	1	3	0
3	5	2	5	5	5	2	3	5	0
5	2	3	5	5	2	3	2	2	1
3	5	1	4	5	4	1	1	5	1
4	4	3	3	4	3	2	2	3	0
5	3	1	3	4	3	3	2	2	1
3	5	3	5	5	4	2	3	4	0
5	5	5	5	4	3	4	5	5	0
5	4	1	5	4	3	3	1	5	0
3	5	3	5	4	3	2	1	5	0
1	2	2	1	5	2	4	1	1	0
3	3	3	4	1	2	3	3	1	0

5	3	3	5	5	2	1	3	5	1
3	5	1	5	5	3	1	1	5	0
1	5	3	5	5	1	1	3	1	1
3	5	5	4	1	3	1	1	2	1
3	5	3	5	2	5	1	1	5	0
3	5	3	5	3	1	1	1	1	1
5	5	5	5	3	3	4	4	2	1
3	3	3	3	3	1	1	3	1	1
1	4	1	3	4	3	1	1	2	0
1	5	1	5	5	5	3	3	5	0
4	4	4	5	4	2	3	3	3	1
1	5	2	5	5	5	1	1	5	0
5	5	3	5	2	1	1	5	1	1
1	4	1	4	5	4	2	4	4	0
1	5	1	5	1	5	1	1	5	0
3	3	4	3	1	3	4	2	3	1
3	5	3	4	5	5	1	1	4	0
3	3	3	5	1	1	3	1	1	0
5	5	1	5	5	3	3	2	3	1
2	5	5	5	5	1	3	1	4	0
3	4	3	3	4	4	2	3	4	0
5	5	5	5	1	3	3	3	5	0
4	4	3	4	1	2	2	2	3	1
1	4	1	4	5	5	1	1	5	0
5	5	5	5	5	3	5	5	3	0
1	5	3	5	3	4	2	1	5	0
3	5	3	5	5	5	1	3	4	0
2	5	2	4	5	4	1	1	5	0
1	5	1	5	5	3	1	1	4	0
1	5	1	5	5	4	1	1	5	0
3	5	1	5	5	4	1	3	4	1
1	5	2	5	5	1	1	1	1	1
5	5	5	5	5	3	3	1	3	1
5	5	1	5	5	1	1	5	3	0
2	5	3	5	5	5	1	1	5	0
3	5	3	5	5	5	2	2	5	0
4	4	3	4	2	4	2	3	4	0
5	5	4	5	5	3	5	5	5	0
3	5	4	5	5	4	3	3	3	0
3	5	3	5	2	5	2	3	5	0
1	5	2	4	4	4	2	1	4	0
3	3	3	2	2	3	3	3	2	0
5	5	5	5	5	5	5	5	5	0
3	5	3	5	5	4	1	3	5	0
4	5	2	5	3	2	2	1	3	0
4	4	3	4	3	3	3	3	1	0
3	5	3	3	5	3	2	1	3	0

4	3	4	3	3	2	2	4	2	0
4	4	5	4	3	2	3	2	1	0
1	5	3	5	5	5	1	1	5	0
3	4	3	5	4	3	3	2	5	0
1	5	1	5	1	1	1	1	1	0
4	5	2	5	4	3	1	1	2	1
4	5	4	5	5	4	2	1	4	1
3	4	3	4	4	3	2	2	3	0
2	4	2	5	5	3	1	1	4	0
4	5	3	4	5	5	1	1	4	0
3	4	3	5	3	3	1	1	3	0
1	5	2	3	2	5	1	1	5	0
3	5	2	5	5	3	3	1	3	0
3	4	4	4	4	4	3	2	4	0
4	5	3	5	3	2	3	3	3	1
3	4	4	4	3	2	1	2	2	1
3	3	4	3	1	3	2	1	3	1
2	4	3	2	4	4	1	1	4	0
2	5	1	5	2	3	3	1	2	0
1	5	1	5	5	5	1	1	5	0
1	5	1	5	5	5	1	1	4	0
5	5	1	5	5	2	1	1	2	0
1	5	1	5	5	4	1	1	5	0
3	5	3	5	5	5	1	1	5	0
1	5	1	5	5	5	1	1	5	0
3	5	2	5	2	5	2	1	5	0
3	3	4	2	3	4	3	2	3	0
3	5	1	5	5	2	1	2	2	1
4	3	3	3	1	2	3	3	2	0
3	4	3	3	3	3	2	3	3	0
4	5	5	4	1	1	3	5	1	1
5	5	4	5	5	1	1	1	1	0
3	3	5	1	1	2	5	1	1	1
3	4	1	4	3	4	2	3	4	0
1	5	1	5	5	4	2	1	3	0
1	5	2	5	5	2	3	3	2	0
3	5	4	3	3	3	2	3	3	0
3	3	3	3	1	2	3	3	3	0
5	5	1	5	2	4	1	2	5	0
5	5	3	5	3	4	1	1	3	0
3	5	3	5	2	4	1	1	4	0
1	5	1	5	5	2	1	3	3	1
3	4	2	5	1	4	3	2	3	0
1	5	1	5	2	5	1	1	4	0
3	5	3	4	3	4	2	1	4	0
1	5	2	5	5	4	2	2	2	0
2	5	4	5	5	1	2	2	1	1

5	5	3	5	5	3	1	5	2	0
3	4	1	4	5	5	1	3	5	0
3	4	2	4	4	4	3	2	5	0
3	5	3	5	5	3	2	3	3	0
3	5	2	5	3	3	1	3	3	0
3	3	4	5	5	5	3	1	4	0
4	4	4	5	4	1	1	3	2	1
5	5	4	5	5	3	1	3	2	0
2	3	3	3	4	3	1	1	3	0
3	5	2	5	5	3	1	3	3	1
5	5	4	5	5	4	1	5	4	0
1	5	2	5	5	3	2	1	4	1
2	5	1	5	1	5	1	1	5	0
4	5	3	5	2	3	3	2	3	0
3	5	3	5	5	5	2	1	5	0
1	5	2	5	5	5	1	1	5	0
4	5	3	5	4	4	3	5	5	0
3	5	1	5	3	2	1	3	2	0
4	5	2	5	2	5	2	2	5	0
3	5	3	5	3	5	2	2	4	0
1	5	4	5	5	5	1	1	5	0
5	5	4	5	3	4	1	1	5	0
2	4	3	5	5	4	1	1	4	0
2	5	3	5	3	4	2	3	5	0
3	5	3	5	5	3	2	3	3	1
1	5	1	5	5	3	1	2	3	0
3	4	5	5	1	1	3	3	2	1
1	5	1	5	5	3	1	1	3	0
5	5	3	5	5	4	1	1	5	0
4	5	1	5	1	5	1	1	3	0
1	5	1	5	4	1	1	1	4	0
2	4	2	4	5	4	2	2	5	1
1	5	1	5	5	4	1	1	3	0
1	5	1	5	5	4	1	1	5	0
3	5	1	5	5	5	1	1	5	0
4	4	3	5	5	4	2	2	5	0
5	5	5	5	1	2	2	1	5	0
3	5	4	5	5	3	3	2	4	0
1	5	2	5	5	3	1	1	3	0
4	4	4	5	5	4	3	1	4	0
1	4	3	4	5	3	3	1	5	0
4	5	4	5	5	4	2	1	4	0
3	5	2	5	5	2	1	1	2	1
1	5	1	5	5	5	1	1	5	0
1	3	3	2	3	3	3	1	1	1
1	5	5	5	3	3	2	3	3	1
2	1	1	3	2	1	4	3	2	1

2	4	1	5	5	4	1	1	4	0
3	4	3	3	1	1	2	3	1	0
2	3	1	1	1	1	1	1	1	1
5	5	5	5	1	3	1	1	3	0
5	3	5	3	3	1	3	3	1	1
3	5	3	5	5	4	3	3	5	0
5	3	5	3	5	2	4	4	2	1
3	5	3	5	5	3	2	2	5	0
5	1	5	5	1	5	5	5	5	0
1	5	1	3	5	3	1	1	5	0
1	5	1	5	5	2	1	1	3	0
2	5	2	5	5	5	2	1	5	0
2	5	2	5	5	1	2	1	1	1
3	1	4	1	1	1	5	1	1	1
1	5	3	3	3	5	1	3	5	1
3	4	3	5	4	4	2	3	4	0
4	4	1	5	5	2	3	1	1	0
2	5	3	4	5	5	3	1	4	0
3	5	3	4	4	2	1	3	1	1
3	5	3	5	5	5	1	3	5	0
3	5	1	5	5	5	1	3	5	0
2	5	1	5	1	2	3	3	2	0
5	5	4	5	3	1	1	5	1	1
4	5	2	5	5	3	1	4	4	1
3	5	3	5	5	5	2	2	5	0
4	5	1	5	5	3	3	5	4	0
3	3	3	3	3	2	1	3	3	0
2	3	3	3	3	3	2	2	3	0
1	5	3	5	5	4	1	2	5	0
3	3	3	3	1	3	1	1	3	0
1	4	1	4	5	4	1	1	4	0
3	3	4	3	4	2	3	1	1	0
4	5	5	5	4	5	3	3	4	1

GDS_2	GDS_3	GDS_4	GDS_5	GDS_6	GDS_7	GDS_8	GDS_9	GDS_10	GDS_11
0	1	1	1	1	1	1	1	0	0
0	0	1	0	1	1	1	1	0	0
1	1	1	1	1	1	1	1	0	1
0	0	0	0	1	0	1	1	0	0
1	1	1	0	1	0	1	0	0	1
0	0	0	0	0	0	0	1	0	0
1	1	1	1	0	1	1	1	1	0
0	0	1	1	1	1	1	1	1	1
0	0	1	0	0	0	0	0	0	0
1	0	0	0	1	0	0	0	0	0
0	0	1	0	1	0	0	1	0	0
0	0	0	0	1	0	0	1	0	0
1	0	0	0	0	0	0	1	0	0
0	0	0	0	1	0	0	1	0	0
0	0	1	0	1	0	0	0	0	0
0	0	0	0	0	0	0	1	0	0
0	0	0	0	0	0	0	0	0	0
0	0	0	0	0	0	0	0	0	0
0	0	0	0	1	0	0	0	0	0
0	0	0	1	0	0	0	1	0	1
0	0	1	0	1	1	1	1	0	0
1	1	0	0	1	0	1	0	0	0
0	0	0	0	0	0	0	1	0	0
1	0	0	0	0	0	0	1	0	0
1	0	0	0	0	0	0	1	0	0
0	0	0	0	0	0	0	1	0	0
0	0	0	0	0	0	0	0	0	0
1	1	1	1	0	1	1	1	0	0
0	0	1	0	0	0	0	1	0	0
0	0	0	0	1	0	0	0	0	0
0	0	0	0	1	0	0	0	0	0
0	0	0	0	0	0	0	1	0	0
1	0	1	0	1	1	0	0	0	0
0	0	0	0	1	0	0	0	0	0
0	0	0	0	0	0	0	1	0	0
1	1	1	1	1	1	0	0	0	0
1	1	1	0	1	0	0	1	0	0
0	0	0	0	0	0	0	1	0	0
0	0	0	0	1	0	0	0	1	0
0	0	0	1	1	1	1	1	0	0
1	0	0	0	0	1	0	1	0	0
0	0	0	0	0	0	0	1	0	0
1	1	0	0	1	1	1	1	1	0
0	0	0	0	0	0	0	0	0	0
1	0	0	0	0	0	0	1	0	0
1	0	0	0	1	0	0	1	0	0

1	1	1	0	1	0	1	0	1	0
1	0	1	0	1	0	0	1	0	0
0	0	0	0	1	0	0	1	0	0
0	0	0	0	0	0	1	1	0	0
0	1	0	0	1	1	1	1	0	0
0	0	1	0	0	1	0	0	0	1
1	1	1	0	1	0	1	1	0	0
1	0	0	0	0	0	0	1	0	0
1	0	0	0	0	0	0	1	0	0
0	0	0	0	0	0	0	0	0	0
1	0	1	0	1	0	1	1	1	0
0	0	0	0	0	0	0	0	0	0
0	1	1	1	1	1	0	1	0	0
1	0	1	0	1	0	0	1	0	0
1	0	0	0	1	0	0	1	0	0
1	0	0	0	1	0	0	1	0	0
0	1	1	0	0	0	1	1	0	0
1	0	0	0	0	0	0	1	0	0
1	1	1	1	1	1	1	1	0	0
1	0	0	0	0	0	0	0	0	0
1	0	1	0	1	0	1	1	0	0
1	1	0	1	1	1	1	1	0	0
1	1	1	0	1	0	0	1	0	0
1	0	0	0	0	0	0	1	0	0
0	0	0	0	0	0	0	1	1	0
1	1	1	1	1	1	1	0	0	0
1	0	0	0	1	0	0	1	0	0
1	1	1	0	0	0	1	0	0	0
0	0	0	0	0	0	0	0	0	0
0	0	0	0	1	0	1	1	0	0
1	0	1	0	0	1	0	1	0	0
0	0	0	0	0	0	0	1	0	0
0	0	1	0	0	0	0	1	0	0
0	0	1	0	1	1	0	1	0	0
0	0	0	0	0	0	0	1	0	0
1	1	1	1	1	0	1	1	1	0
1	0	0	0	0	0	0	1	1	0
0	0	0	0	0	0	0	0	0	0
1	1	0	0	1	0	0	1	0	0
1	0	0	0	1	0	0	0	0	0
1	1	1	1	1	1	1	1	1	0
0	0	0	0	0	0	0	1	0	0
1	1	0	0	0	0	1	0	1	0
0	0	1	0	1	0	0	1	0	0
0	0	0	0	0	0	0	1	0	0
0	0	0	0	0	0	0	0	0	0
0	0	0	0	0	0	0	0	0	0

1	1	1	0	1	1	1	1	1	0
0	0	0	0	0	0	0	0	0	0
1	1	1	0	1	1	0	0	0	0
0	0	0	0	0	0	0	0	0	0
1	1	1	1	1	1	1	1	1	0
1	0	1	0	1	1	1	1	0	0
1	1	1	0	0	1	0	1	0	0
0	1	1	0	0	0	1	1	0	0
0	0	0	0	0	0	0	1	0	0
0	0	0	0	1	0	0	0	0	0
0	0	0	0	0	0	0	0	0	0
1	0	0	0	0	0	0	1	0	0
0	0	0	0	0	0	0	0	0	0
0	0	0	0	0	0	0	0	0	0
0	0	0	0	0	0	0	0	0	0
1	1	0	0	1	1	1	0	0	0
0	1	1	0	1	1	1	1	1	0
0	0	0	1	0	0	0	1	0	0
1	0	1	1	0	1	1	1	0	0
0	0	0	0	0	0	0	0	0	0
0	0	1	0	1	0	0	0	0	0
0	0	0	0	1	1	0	1	1	0
0	1	1	1	1	0	1	0	1	0
1	0	0	0	1	0	0	0	0	0
1	0	0	0	0	0	0	0	0	0
1	0	1	1	0	0	0	0	0	0
0	1	1	1	1	1	1	1	0	0
1	1	1	0	0	0	0	0	0	0
1	0	0	0	0	0	1	0	0	1
1	0	1	0	1	1	1	1	0	0
0	0	0	0	0	0	0	0	0	0
0	0	0	0	0	0	0	0	0	0
1	1	1	1	1	1	1	1	1	1
0	0	0	0	0	0	0	1	0	0
1	0	1	0	1	1	0	0	0	0
0	0	0	0	1	0	0	1	0	0
1	1	1	0	1	0	1	1	1	0
1	0	1	0	1	1	1	1	1	0
0	0	1	0	1	0	0	0	0	0
1	0	0	0	1	0	1	1	0	0
1	0	0	0	0	0	0	1	0	0
0	0	0	0	0	0	0	1	0	0
0	0	0	0	0	0	0	1	0	0
0	1	1	1	1	1	1	1	0	0
1	0	0	0	1	0	1	0	0	0
0	0	1	0	1	0	1	1	1	0
1	0	0	0	0	0	1	1	0	0

0	0	0	0	0	0	0	1	0	0
1	0	1	0	0	0	0	1	0	0
1	1	1	1	1	1	1	0	0	0
1	0	1	0	0	0	0	1	1	0
1	1	1	1	1	1	1	1	1	0
1	0	0	0	0	0	0	1	0	0
0	0	0	0	1	0	0	0	0	0
1	1	0	1	0	1	1	1	0	0
1	0	0	0	0	0	0	1	1	0
1	0	1	0	0	0	0	0	0	0
0	0	0	1	0	0	0	0	0	0
1	1	1	1	1	1	1	0	0	0
0	0	0	0	1	0	0	1	0	0
1	0	0	0	0	0	0	0	0	0
0	0	0	0	1	0	0	1	0	0
1	1	0	1	1	1	0	0	0	0
0	0	0	0	0	0	0	0	0	0
1	1	1	0	0	0	1	0	0	0
0	0	0	0	0	0	0	1	0	0
0	0	0	0	0	0	0	0	0	0
0	0	0	0	0	0	0	0	0	0
1	0	0	0	1	0	1	1	0	0
0	0	0	0	1	0	0	0	0	0
1	0	0	1	1	1	0	0	0	0
0	1	1	0	0	0	1	1	0	0
0	0	0	0	0	0	0	1	0	0
1	1	1	0	1	1	0	0	0	0
0	0	0	0	0	0	0	1	0	0
1	1	1	1	1	1	1	1	0	0
1	0	0	0	0	1	1	0	0	0
0	0	0	1	1	1	0	1	0	0
0	0	0	0	0	0	0	1	0	0
0	0	1	0	1	0	0	1	0	0
0	0	0	0	0	0	0	0	0	0
0	0	0	0	0	0	0	1	0	0
0	0	0	0	1	0	0	0	0	0
1	0	1	0	0	0	0	1	1	0
1	0	0	0	0	0	0	0	0	0
0	0	0	0	0	0	0	1	0	0
0	0	0	0	0	0	0	1	0	0
0	1	0	0	1	0	0	1	0	0
0	0	0	0	0	0	0	0	0	0
0	0	0	0	1	0	0	1	1	0
0	0	0	0	0	0	0	0	0	0
0	0	0	0	0	0	0	0	0	0
0	0	1	1	1	1	1	1	1	0
0	0	0	0	0	0	0	0	0	0

1	1	0	0	0	0	0	1	0	0
1	1	1	0	1	0	0	0	1	0
1	0	0	0	0	0	0	0	1	0
1	1	1	1	0	1	1	1	0	0
1	1	0	0	1	0	1	1	0	0
0	1	0	0	1	0	0	1	0	0
1	1	1	0	1	1	1	1	1	0
0	0	1	0	1	0	0	1	0	0
1	0	1	0	0	0	0	1	0	0
0	0	0	0	0	0	0	0	0	0
1	1	1	1	1	1	1	1	0	0
0	0	1	0	1	0	0	0	0	0
1	0	1	0	0	0	0	1	0	0
0	0	0	0	1	0	0	1	1	0
0	0	0	0	1	0	0	1	0	0
0	0	1	0	1	0	0	1	0	0
1	1	1	1	1	1	1	1	0	0
0	0	1	0	0	0	0	0	0	1
0	0	0	0	0	1	0	1	0	0
1	1	1	1	1	0	0	1	0	0
0	0	0	0	1	0	0	0	1	0
1	0	0	0	0	0	0	0	0	0
0	0	0	0	0	0	0	1	0	0
0	0	0	0	0	0	0	1	0	0
0	0	0	0	0	0	0	0	0	0
1	0	0	0	1	0	0	0	1	0
0	0	0	0	0	0	0	1	0	0
0	0	0	0	0	0	0	0	0	0
1	0	0	0	1	1	0	0	0	0
0	0	1	0	0	0	0	0	0	0
0	0	0	0	0	0	0	1	0	0
1	0	1	0	1	0	1	0	1	0
0	0	1	0	1	0	1	0	1	0
0	0	0	0	0	0	0	0	0	0
1	1	0	1	0	1	0	0	0	0
0	0	0	0	0	0	0	0	0	0
1	0	0	0	1	0	1	0	0	0
0	1	1	1	1	1	1	1	0	1
1	1	1	1	0	1	1	1	1	1
0	0	1	0	0	0	0	0	0	0
0	0	0	0	0	0	0	0	0	0
1	1	0	0	1	0	1	0	0	0
1	1	1	1	1	1	1	1	0	0
1	0	1	0	1	1	1	0	1	0
0	0	0	0	0	0	0	1	1	0
0	0	0	0	0	0	0	1	0	0
1	1	1	1	0	0	0	1	0	1

1	1	1	0	0	1	0	1	1	0
0	0	0	0	0	0	0	0	0	0
0	0	0	0	0	0	0	1	0	0
0	1	0	0	1	1	1	1	0	0
1	0	0	0	1	0	0	0	0	0
1	1	1	1	0	1	1	0	0	1
0	0	0	0	0	0	0	1	0	0
0	0	0	0	0	0	0	1	0	0
0	0	0	0	1	0	0	0	0	0
1	1	1	1	1	1	1	1	1	0
1	0	1	1	1	1	0	1	0	0
0	1	0	0	0	0	1	1	0	0
1	1	1	1	1	1	1	1	0	0
1	0	0	0	0	1	0	1	0	0
1	0	0	0	0	0	0	1	1	0
0	0	0	0	1	0	0	0	0	0
1	0	0	0	1	0	1	1	0	0
1	1	1	1	1	1	1	0	0	0
0	0	0	0	0	0	0	0	0	0
0	0	1	0	0	0	0	1	0	0
1	0	0	0	0	0	0	0	0	0
1	0	0	0	0	0	0	0	0	0
0	0	0	0	1	0	0	0	0	0
1	0	0	0	0	0	0	0	0	0
0	0	0	0	0	0	0	1	0	0
0	0	0	0	1	0	0	0	0	0
0	0	0	0	0	0	0	1	0	0
1	0	0	0	0	0	0	1	0	0
1	1	0	1	0	1	1	1	1	0
1	1	0	1	1	1	1	1	0	0
1	0	0	0	0	0	0	0	0	0
1	0	0	0	1	0	1	0	0	0
0	0	0	0	0	0	0	0	0	0
1	0	1	0	1	1	0	1	0	0
0	0	0	0	0	0	0	0	0	0
0	0	0	0	0	0	0	1	0	0
1	0	0	0	0	0	0	1	0	0
0	0	0	0	0	0	1	1	1	0
1	1	1	1	1	1	1	0	0	0
1	0	0	0	0	1	0	0	0	0
1	1	0	0	0	1	1	1	0	0
1	0	1	0	1	0	0	1	1	0
1	0	0	0	1	0	1	0	0	0
0	0	0	0	0	0	0	1	0	0
1	1	1	1	1	1	1	1	0	0
0	0	0	0	1	0	0	0	0	0
0	0	0	0	0	0	0	0	0	0

0	0	0	0	0	0	0	0	0	0
1	1	1	1	1	1	1	1	1	0
0	0	0	0	0	0	1	0	0	0
1	0	0	0	1	1	0	0	0	0
0	0	0	0	0	0	0	0	0	0
1	1	1	1	1	1	1	1	0	0
1	0	0	0	0	0	0	0	0	0
0	0	0	0	1	0	0	0	0	0
1	1	1	1	1	1	1	0	0	0
1	1	1	1	0	1	1	1	0	0
0	0	0	0	0	0	0	1	0	0
0	0	0	0	0	0	0	1	0	0
1	0	1	0	1	0	0	1	1	0
0	0	1	0	0	0	0	1	0	0
0	0	0	0	0	0	0	1	0	0
1	0	1	1	1	1	1	0	1	0
0	0	0	0	1	1	1	0	0	0
0	0	0	0	0	0	0	1	0	0
0	1	0	0	0	0	0	1	0	0
1	0	0	0	1	0	1	1	1	0
0	0	1	0	0	0	1	1	0	0
1	0	0	0	1	0	0	1	0	0
0	0	0	0	0	0	0	0	0	0
0	0	0	0	1	0	0	0	0	0
1	0	1	0	0	0	1	1	0	0
0	0	0	0	1	0	0	1	0	0
1	0	1	0	1	0	0	0	0	0
1	0	1	0	1	0	0	0	0	0
0	0	0	0	1	0	0	1	0	0
0	1	1	1	1	1	1	1	0	0
0	0	0	0	0	0	0	1	0	0
1	0	0	0	0	0	0	1	0	0
0	0	1	1	1	1	1	1	1	1
1	0	0	0	1	0	0	1	0	0
0	0	0	0	0	0	0	0	0	0
1	1	1	1	1	1	1	1	1	1
1	1	0	1	0	1	1	1	0	1
0	0	0	0	0	0	0	1	0	0
0	0	0	0	0	0	0	1	0	0
0	0	1	0	0	0	0	0	0	0
1	1	1	1	1	1	1	1	1	1
0	0	1	0	0	1	0	0	0	0
0	0	0	0	1	0	0	0	0	0
0	0	0	0	0	0	0	0	0	0
1	1	1	0	1	0	0	1	1	0
0	0	0	0	1	0	0	1	0	0
0	1	1	1	1	1	1	1	1	0

0	0	0	0	0	0	0	1	0	0
0	0	0	0	0	0	0	0	0	0
0	0	0	0	1	0	0	1	0	0
0	1	1	0	1	0	0	0	0	0
0	0	0	0	1	0	0	1	0	0
0	0	0	0	0	0	0	0	0	0
0	0	0	0	0	0	0	1	0	0
1	1	0	0	1	1	0	1	0	0
1	1	1	1	1	1	1	1	1	0
1	0	1	1	1	1	1	0	1	1
1	0	1	1	0	1	0	0	0	0
1	1	0	1	1	1	1	1	0	0
0	0	0	0	1	0	0	0	0	0
0	0	0	0	0	0	0	1	0	0
0	0	0	0	0	0	0	1	1	0
1	0	1	0	1	0	0	1	0	0
0	0	0	0	0	0	0	1	0	0
0	0	0	0	0	0	0	0	0	0
0	0	0	0	1	0	0	0	0	0
1	0	0	0	0	0	0	1	0	0
1	1	0	1	0	1	1	1	0	0
0	0	0	0	1	0	1	1	0	0
0	0	1	0	0	0	0	1	0	0
0	1	1	1	0	1	0	1	0	0
0	0	0	0	1	0	0	0	0	0
1	1	0	0	1	0	1	1	0	0
1	1	1	0	1	0	1	1	0	0
0	0	0	0	0	0	0	1	0	0
0	0	0	0	0	0	0	0	0	0
1	0	1	0	1	0	0	0	0	0
0	0	0	0	0	0	0	0	0	0
1	1	0	0	0	0	0	1	0	0
0	0	0	0	0	0	0	1	1	0
0	0	0	0	0	0	0	0	0	0
1	0	1	0	1	1	0	1	0	0
1	1	1	1	1	1	1	1	1	1
0	0	0	0	0	0	0	1	0	0
0	0	0	0	0	0	0	1	0	0
0	0	0	0	1	0	0	0	0	0
0	0	0	0	1	0	0	0	0	0
0	0	0	0	1	0	0	0	0	0
0	0	0	0	0	0	0	1	0	0
1	1	1	0	0	0	1	1	0	0
0	0	0	0	0	0	0	1	0	0
0	0	0	0	0	0	0	1	0	0
1	0	0	0	1	0	1	0	0	0
0	0	0	0	0	0	0	0	0	0

1	0	0	0	1	0	0	1	1	0
1	0	0	0	0	0	0	1	0	0
1	0	0	0	1	0	0	1	1	0
0	0	0	0	1	1	0	1	0	0
0	0	0	0	0	0	0	0	0	0
0	0	0	0	0	0	0	0	0	0
1	0	1	0	1	0	0	0	0	0
1	0	1	0	1	0	0	1	0	0
1	1	1	1	0	1	1	1	0	0
0	0	0	0	0	0	0	1	0	0
1	0	1	0	0	0	0	1	0	0
0	1	1	1	1	1	1	1	0	0
0	0	1	0	0	0	0	0	0	0
1	1	1	0	1	0	1	1	1	0
1	0	1	0	1	0	1	1	1	0
1	1	1	1	0	0	0	1	0	0
0	0	1	0	0	0	0	0	0	0
1	0	1	0	1	0	0	0	0	0
1	0	0	0	0	0	0	1	1	0
1	0	0	0	1	1	0	0	0	0
0	1	0	0	0	0	0	0	0	0
1	0	0	0	0	0	0	1	0	0
0	0	1	0	0	0	0	1	0	0
1	1	1	1	0	1	1	1	0	0
1	0	1	0	0	0	1	1	1	0
0	0	1	0	0	1	0	0	0	0
0	0	0	0	1	0	0	1	0	0
0	0	0	0	0	0	0	1	0	0
0	0	0	0	1	0	0	0	1	0
1	1	0	0	1	0	0	0	0	0
0	0	0	0	0	0	0	0	0	0
0	0	0	0	0	0	0	1	0	0
0	0	1	0	0	0	1	0	0	0
0	0	0	0	0	0	0	0	0	0
0	0	0	0	0	0	0	0	0	0
1	0	0	0	1	0	0	1	0	0
0	0	0	0	0	0	0	1	0	0
0	1	1	1	0	1	1	1	0	0
0	0	0	0	0	0	0	1	0	0
1	0	0	0	1	1	0	1	0	0
1	1	1	1	1	1	0	0	0	0
1	0	0	0	0	0	0	1	1	0
1	0	1	0	0	0	0	1	0	0
1	0	1	0	0	0	0	1	0	0
0	0	0	0	0	1	0	1	0	0
1	1	0	0	1	0	1	1	0	0
1	0	0	0	0	0	0	1	0	0

1	1	0	0	1	0	1	0	1	0
0	0	0	0	0	0	0	0	0	0
1	1	1	0	0	0	1	0	0	0
1	1	1	1	1	1	1	0	1	0
1	0	1	0	0	0	0	1	0	0
1	0	0	1	0	1	0	1	0	0
1	1	1	1	1	1	1	0	1	1
1	1	1	1	1	1	1	1	0	0
0	1	0	0	0	0	0	1	1	0
1	0	0	0	0	0	0	0	0	0
1	1	1	1	1	0	1	1	0	0
0	1	0	0	0	1	0	0	0	0
0	1	1	1	1	1	1	1	0	0
0	0	0	1	0	1	0	0	0	0
0	0	0	0	0	0	0	0	0	0
1	1	1	1	1	1	1	1	1	1
1	0	0	0	1	0	0	0	0	0
1	1	1	1	1	0	0	0	0	0
0	1	1	0	0	0	0	0	0	0
1	1	1	1	1	0	1	1	0	0
0	0	1	0	1	0	0	0	0	0
1	0	1	0	0	0	0	1	0	0
1	1	1	1	1	1	1	0	0	0
0	0	0	0	0	0	0	1	0	0
1	1	0	0	0	0	0	1	1	0
1	0	0	0	0	0	0	0	0	0
1	0	0	0	1	0	0	1	0	0
0	0	0	0	0	0	0	0	0	0
0	0	1	0	0	0	0	1	0	0
0	0	1	0	0	0	0	1	0	0
1	0	0	0	1	0	0	1	1	0
1	1	1	0	0	1	1	0	0	0
1	1	1	1	1	1	1	1	1	0
0	0	1	0	0	0	0	1	0	0
0	0	0	0	0	0	0	0	0	0
0	0	0	0	0	0	1	0	0	0
0	0	0	0	1	0	0	0	0	0
0	0	0	0	0	0	1	1	0	0
1	0	0	0	1	0	0	0	0	0
0	0	0	0	0	0	0	0	0	0
0	0	0	0	1	0	0	1	0	0
0	0	0	0	1	0	0	1	0	0
1	0	0	0	0	0	0	0	0	0
0	0	0	0	0	0	0	1	0	0
0	0	0	0	1	0	0	0	1	0
0	0	0	0	0	0	0	1	0	0
0	0	0	1	0	0	0	0	0	0

0	0	0	0	1	0	0	1	1	0
0	1	1	1	1	1	0	0	0	0
0	0	0	0	0	0	0	0	0	0
1	0	0	0	1	0	0	1	0	0
0	0	0	0	0	0	0	0	0	0
1	1	1	1	0	1	0	1	0	0
1	1	1	1	1	0	0	1	1	1
0	0	0	0	0	0	0	1	0	0
0	0	1	0	1	0	0	1	0	0
1	0	0	0	1	0	0	1	1	0
0	0	0	0	0	0	0	1	0	0
0	0	0	0	0	0	0	1	0	0
0	0	0	0	0	1	0	0	0	0
1	0	1	0	0	0	0	0	0	0
1	0	1	1	1	1	0	1	0	0
1	1	1	1	1	1	0	1	0	1
1	0	0	0	1	0	0	0	0	0
0	0	0	0	0	0	0	0	0	0
0	0	0	0	0	0	0	1	1	0
0	0	0	0	0	0	0	0	0	0
0	0	0	0	0	0	0	0	0	0
0	0	0	0	0	0	0	1	1	0
0	0	0	0	1	0	0	0	0	0
0	0	0	0	0	0	0	1	0	0
0	0	1	0	1	1	1	1	1	0
0	0	1	0	0	0	0	0	0	0
0	0	0	0	1	0	0	0	0	0
1	1	1	1	0	1	1	1	0	1
1	0	0	0	1	0	0	1	0	0
1	0	1	0	1	0	1	0	0	0
0	1	1	1	1	1	1	1	1	0
1	0	0	0	0	0	0	1	0	0
1	1	1	1	1	1	1	1	1	0
0	0	0	0	1	0	0	0	0	0
0	0	0	0	0	0	0	1	0	0
0	0	0	0	1	0	0	1	0	0
0	0	0	0	1	0	1	1	0	0
0	0	0	0	1	0	0	1	0	0
0	0	0	0	1	0	0	0	1	0
1	1	0	0	0	1	0	0	0	0
0	0	0	0	0	0	0	0	0	0
1	0	1	0	1	1	1	1	0	0
0	0	1	0	1	1	0	1	1	0
0	0	0	0	1	0	0	0	0	0
1	0	0	0	0	0	0	1	0	0
0	0	1	0	1	0	1	1	1	0
1	1	0	0	1	1	1	1	0	0

0	0	0	0	1	0	0	1	0	0
0	0	1	0	0	0	0	1	0	0
0	0	0	0	0	0	0	1	0	0
1	0	0	0	1	0	0	0	1	0
0	0	0	0	1	0	0	0	0	0
0	0	0	0	0	0	0	0	0	0
1	1	0	0	0	0	0	1	0	0
1	1	1	0	1	1	1	1	0	0
1	1	1	1	0	0	0	1	0	0
1	0	0	0	0	0	1	0	0	0
0	0	1	0	1	0	0	1	0	0
0	0	1	0	0	0	1	1	0	0
0	0	0	0	0	0	0	1	0	0
0	0	0	0	0	0	0	1	0	0
0	0	0	0	0	1	0	1	0	0
0	0	0	0	0	0	0	0	0	0
0	0	0	1	1	1	0	0	0	0
1	1	1	0	0	1	0	1	0	0
1	0	0	0	1	0	0	0	0	0
0	0	0	0	0	0	0	0	0	0
0	1	0	0	1	1	1	0	0	0
0	0	0	0	0	0	0	0	0	0
0	0	1	0	1	0	0	0	0	0
1	0	0	0	0	0	0	1	0	0
1	1	0	1	1	1	1	0	0	0
0	0	0	0	0	0	0	1	0	0
1	1	1	1	0	1	1	1	1	0
0	0	0	0	0	0	0	0	0	0
0	0	0	0	0	0	0	1	1	0
0	1	0	0	1	1	1	1	0	0
0	0	0	0	0	0	0	0	0	0
1	0	0	0	0	0	0	0	0	0
0	0	0	0	0	0	1	0	0	0
0	0	0	0	0	0	0	0	0	0
0	0	1	0	0	0	0	1	1	0
0	0	0	0	0	0	0	0	0	1
1	0	1	0	0	0	0	1	0	0
0	0	1	0	1	0	0	0	1	0
1	0	0	0	0	0	0	0	0	0
0	0	1	0	0	0	0	0	0	0
0	0	0	0	1	0	1	0	1	0
1	0	0	1	1	1	0	0	1	0
0	1	1	0	0	0	0	0	0	0
0	0	0	0	0	0	0	1	0	0
0	1	1	0	1	1	1	1	0	0
0	1	0	0	0	1	0	0	0	0
1	1	1	1	1	1	0	1	0	0

0	0	0	0	0	0	0	1	0	0
1	0	0	0	1	0	0	1	0	0
1	1	1	1	1	1	1	1	1	1
1	1	0	0	0	0	0	1	0	0
1	1	1	1	1	1	1	1	0	0
0	0	0	0	0	0	0	1	1	0
1	1	1	1	1	1	1	1	0	0
0	0	0	0	0	1	0	1	1	0
0	1	1	1	1	1	0	1	1	0
0	0	0	0	0	0	0	0	0	0
0	0	0	0	0	0	0	1	0	0
0	0	0	0	0	0	0	0	0	0
1	0	0	1	0	1	0	0	0	0
1	1	1	1	1	1	1	1	0	0
1	1	0	1	0	0	1	0	1	0
0	0	0	0	1	0	0	1	0	0
0	0	0	0	1	0	0	1	0	0
0	0	1	0	0	0	0	1	0	0
1	1	1	1	0	1	1	1	0	0
0	0	0	0	1	0	0	0	0	0
0	0	1	0	1	0	0	1	0	0
1	0	0	0	1	1	0	1	0	0
1	1	1	1	1	1	1	1	0	0
1	0	1	1	0	1	0	1	1	0
0	0	0	0	0	0	0	1	0	0
0	0	0	0	1	0	1	1	0	0
1	1	0	0	0	1	0	1	0	0
0	0	0	0	0	0	0	0	0	0
0	0	0	0	0	0	0	0	0	0
0	1	0	0	1	1	1	1	0	0
0	0	0	0	0	0	0	0	0	0
1	0	1	1	0	1	0	1	1	0
1	0	1	1	1	0	1	0	0	0

GDS_12	GDS_13	GDS_14	GDS_15	old_1	old_2	old_3	old_4	old_5	old_6
0	0	0	1	4	4	5	4	3	4
0	0	0	0	5	4	4	5	5	5
1	1	1	1	5	5	4	3	4	5
0	0	1	0	2	4	4	4	4	5
0	0	1	1	4	4	4	1	4	1
1	0	0	0	5	5	5	5	5	5
0	1	1	1	3	2	4	3	4	3
1	1	1	1	2	2	2	2	3	5
0	0	0	0	4	5	4	2	4	5
0	0	0	0	5	5	4	4	5	1
0	0	0	0	4	5	4	3	3	2
1	0	0	0	3	3	4	4	4	2
0	0	0	0	4	4	3	4	3	2
0	0	0	0	3	3	4	3	3	3
0	0	0	0	4	5	5	4	4	4
0	0	0	0	4	5	4	4	4	5
0	0	0	0	4	4	5	5	4	2
0	0	0	0	4	4	4	4	4	4
0	0	0	0	5	5	4	4	4	3
0	0	0	0	4	5	5	4	4	5
0	0	0	0	3	3	4	4	3	3
0	0	0	0	5	5	3	2	4	2
0	0	0	0	5	4	4	3	4	5
0	0	0	0	4	4	4	4	4	4
0	0	0	0	4	4	4	4	4	4
0	0	0	0	4	5	5	4	5	3
0	0	1	0	4	4	5	4	4	4
0	1	1	1	5	5	4	2	2	2
0	0	0	1	4	5	4	1	4	5
0	0	0	0	3	4	3	3	3	3
0	0	0	0	3	4	3	3	3	3
0	0	0	0	5	5	1	4	4	5
0	1	0	0	2	2	3	3	4	3
1	0	0	0	5	5	4	4	4	5
0	0	0	0	4	4	4	4	4	4
0	1	0	1	3	3	5	5	4	1
0	1	1	1	2	2	5	2	4	2
0	0	0	0	4	4	3	2	4	5
0	0	0	0	4	4	5	5	5	5
0	1	0	0	4	4	4	4	4	1
0	1	0	0	3	3	4	4	4	3
0	0	1	0	5	5	5	5	5	4
1	1	1	1	3	2	2	2	2	5
0	0	0	0	4	4	4	4	4	3
0	0	0	0	5	5	4	1	5	5
0	0	0	0	3	3	4	3	4	2

0	1	0	0	2	2	4	4	3	4
1	0	0	0	2	2	3	3	3	3
0	0	0	0	4	4	5	5	5	4
0	0	0	0	4	5	4	4	4	4
0	0	1	1	5	5	4	2	2	4
0	0	0	0	4	4	3	4	4	5
0	0	1	1	5	5	4	3	2	5
0	0	0	0	3	4	4	3	4	3
0	1	0	0	5	5	5	4	5	5
0	0	0	0	5	5	4	1	4	5
0	1	0	1	3	2	3	4	4	2
0	0	0	0	2	2	4	4	4	2
0	0	0	0	4	4	4	4	4	2
0	0	0	0	2	4	4	4	4	5
0	0	1	0	3	3	3	4	4	5
0	0	1	0	3	3	3	4	4	5
0	0	0	0	1	1	5	4	5	5
0	0	0	0	4	4	5	5	4	4
0	1	1	0	4	4	4	3	4	4
0	0	0	0	5	5	4	4	4	5
0	0	0	1	4	4	4	3	3	4
0	1	1	1	3	4	4	4	4	4
0	0	0	0	3	2	4	2	2	4
0	0	0	0	3	4	4	4	4	5
0	0	0	1	1	1	4	3	3	5
0	0	1	1	3	5	1	2	1	4
0	0	1	0	4	3	4	4	4	5
0	0	0	0	2	3	4	3	4	4
0	0	1	0	2	5	4	4	4	4
0	1	0	0	5	5	4	4	4	4
0	0	0	0	5	5	5	4	4	5
0	0	0	0	4	4	4	3	3	3
0	0	0	0	5	5	4	4	4	5
0	0	0	0	3	3	5	4	4	3
0	0	0	0	5	5	4	3	3	2
0	1	0	0	3	3	4	2	2	3
0	0	0	0	2	2	4	3	4	5
0	0	0	0	5	5	5	5	5	5
0	1	0	0	4	5	4	2	4	3
0	0	0	0	4	4	4	4	4	2
1	0	1	0	5	4	4	4	4	5
0	0	0	0	5	5	4	4	4	4
0	0	0	1	4	4	4	2	4	5
0	0	1	1	2	2	4	4	3	2
0	0	0	0	5	5	4	2	4	5
0	0	0	0	5	5	5	4	4	5
0	0	0	0	5	5	5	4	4	5

1	1	1	1	4	5	4	2	4	3
0	0	0	0	4	5	5	4	4	5
0	0	0	0	1	2	4	5	5	5
0	0	0	0	4	4	4	4	5	3
0	0	1	1	5	5	4	4	2	2
0	0	0	0	4	4	5	2	2	4
0	1	1	1	5	4	2	1	1	4
0	0	0	0	5	4	2	3	3	5
0	0	0	0	5	5	4	4	5	5
0	0	0	0	2	2	5	3	5	2
1	1	0	0	4	4	4	4	4	4
0	0	0	0	4	4	4	3	4	3
0	0	0	0	4	4	5	5	5	5
0	0	0	0	4	5	5	5	5	4
0	0	0	0	5	5	5	1	5	5
0	0	0	1	5	5	4	5	4	1
1	0	1	1	2	4	2	1	4	4
0	0	0	0	4	4	4	4	4	3
0	0	0	0	5	5	5	4	4	4
0	0	0	0	5	5	4	4	4	5
0	0	1	0	3	3	3	3	3	2
0	0	0	0	5	5	4	2	4	4
1	0	1	1	1	4	1	2	5	3
0	0	0	0	2	5	4	4	4	1
1	0	0	0	5	5	4	1	4	3
0	0	0	1	4	4	4	4	1	5
0	1	0	1	5	5	4	1	2	5
0	1	0	0	3	2	3	2	2	5
0	0	0	0	3	4	4	2	4	4
0	0	0	1	4	5	4	4	3	2
0	0	0	0	4	4	5	5	5	5
0	0	0	0	5	5	1	5	4	5
0	1	1	1	2	2	5	5	1	2
0	1	0	0	3	4	3	3	3	3
1	0	0	0	4	5	4	4	3	2
0	0	0	0	4	5	3	4	5	5
1	1	1	1	4	2	4	3	2	2
1	1	1	1	2	2	2	3	3	3
0	0	0	0	4	4	4	4	4	2
0	1	0	0	4	5	5	4	3	5
0	0	1	0	4	4	4	4	4	5
0	0	1	0	5	4	4	4	4	4
0	0	0	0	4	4	4	5	4	4
1	1	1	1	4	4	2	2	2	2
0	0	0	0	5	5	4	4	3	5
1	0	0	0	4	4	5	3	4	1
1	0	0	0	5	5	5	2	3	5

0	0	0	0	4	5	5	5	4	5
0	0	0	0	3	3	2	2	4	2
1	1	1	1	1	1	4	3	5	4
1	1	0	0	4	4	4	4	3	4
1	1	1	1	3	4	3	1	4	2
0	0	0	0	4	4	4	3	5	5
0	0	0	0	4	4	5	5	3	5
0	0	1	1	4	4	5	3	3	5
0	0	0	0	4	4	4	4	4	5
0	0	1	0	3	4	5	5	5	5
0	0	0	0	1	5	5	4	4	5
1	0	1	1	4	5	5	1	1	1
0	0	0	0	3	3	3	3	3	2
0	0	1	0	5	5	4	4	5	5
0	0	0	0	5	5	4	2	4	2
1	1	1	0	3	5	2	2	2	5
0	0	0	0	5	5	4	5	5	5
1	0	1	1	5	5	3	3	3	5
0	0	0	0	2	5	4	3	4	5
0	0	0	0	5	5	4	5	4	4
0	0	0	0	5	5	4	4	4	5
0	1	1	0	1	2	5	1	5	2
0	0	0	0	4	4	4	4	4	4
0	0	0	0	5	5	2	4	4	3
0	0	1	0	3	3	4	3	4	4
0	0	0	0	3	4	5	2	4	5
0	1	0	0	4	3	3	3	3	3
0	0	0	0	4	4	5	4	4	4
0	0	1	1	5	5	5	3	3	1
0	1	0	0	2	2	5	5	5	5
1	1	1	1	4	3	4	3	4	4
1	0	1	0	5	5	5	5	5	5
0	0	0	1	5	5	4	3	4	3
0	0	0	0	4	3	5	4	5	2
0	0	0	0	4	5	4	2	3	5
0	1	0	0	3	3	4	3	4	3
0	0	0	0	4	3	4	3	4	4
0	0	0	0	5	5	4	4	4	5
0	0	0	0	4	4	1	5	4	5
0	0	0	0	5	5	5	5	5	5
0	1	0	0	5	4	3	4	2	5
0	0	0	0	5	5	5	5	5	5
0	0	0	0	4	4	4	4	4	2
0	0	0	0	5	4	5	5	5	5
0	0	0	0	5	5	4	4	4	4
0	0	0	0	5	5	4	4	4	4
0	0	0	0	3	5	5	4	5	5

1	0	0	0	5	5	4	4	4	4
0	1	0	0	5	4	3	3	2	5
0	1	0	0	3	2	5	5	4	5
1	1	0	1	3	4	4	3	3	4
0	0	0	0	4	4	4	4	4	5
0	0	0	0	4	4	4	4	4	5
0	1	0	0	2	4	4	4	3	2
0	0	0	0	4	4	4	3	2	2
1	0	0	0	5	5	5	5	5	5
1	0	0	0	4	5	4	4	4	5
0	1	0	0	3	3	3	2	2	2
0	0	0	0	2	3	4	1	1	2
1	0	1	1	5	5	4	3	4	3
0	0	0	0	4	3	5	2	4	4
0	0	0	0	5	5	4	4	4	5
0	0	0	0	5	5	4	4	3	4
0	0	0	1	3	4	4	2	3	4
0	0	1	0	5	5	5	5	5	5
0	0	1	0	1	3	5	4	3	5
1	1	0	0	4	5	5	2	1	3
1	0	1	0	4	4	4	4	4	2
0	0	0	0	4	4	4	4	4	4
0	0	0	0	3	4	4	2	4	4
0	1	0	0	3	2	4	4	4	4
0	0	0	0	5	5	5	5	5	5
0	0	0	0	3	3	5	4	4	2
0	0	1	0	3	4	4	4	4	4
0	0	0	0	4	5	5	5	5	4
0	1	0	1	4	5	4	2	3	2
0	0	0	0	4	5	5	4	4	5
0	0	0	0	4	4	5	5	5	3
1	1	0	0	3	3	4	3	3	4
0	1	0	0	3	4	4	3	4	4
0	0	0	0	5	4	4	4	4	5
0	1	1	0	5	5	4	3	3	5
0	0	0	0	4	5	5	5	4	3
0	0	0	1	4	4	5	2	3	3
1	0	1	1	5	5	4	1	2	2
1	0	1	0	5	5	3	1	3	5
0	0	0	0	5	5	5	5	5	5
0	0	0	0	5	5	4	5	5	5
1	0	0	0	4	5	4	2	3	5
1	1	1	1	3	4	4	4	2	2
0	0	0	0	3	3	4	4	4	3
0	0	1	0	1	1	4	4	2	5
0	0	0	0	5	5	4	1	4	5
0	1	1	0	3	5	5	4	4	4

1	1	0	1	2	2	3	2	2	4
0	0	0	0	5	5	1	1	1	5
0	0	1	0	5	5	4	5	4	5
0	0	0	0	5	5	5	4	4	3
0	0	1	0	5	5	4	1	5	5
1	1	1	1	5	5	4	2	4	5
1	0	0	0	4	5	4	3	4	4
0	0	0	0	3	5	4	4	3	4
0	0	0	0	5	5	5	5	5	5
0	0	1	0	2	2	3	3	4	3
0	0	0	0	4	4	4	3	3	5
1	1	1	0	2	4	4	4	4	4
1	1	1	1	5	5	4	3	2	2
0	0	1	0	4	3	3	3	3	3
0	1	0	0	2	2	5	1	4	5
0	1	0	0	4	4	4	4	3	4
0	0	0	0	5	5	5	5	4	2
1	0	0	0	4	4	4	4	3	3
0	0	0	0	5	5	3	3	3	5
0	0	1	0	4	4	5	5	3	5
0	1	0	0	3	4	5	4	5	5
0	0	0	0	4	4	3	3	3	5
0	0	0	0	4	4	4	3	4	2
0	0	0	0	5	5	5	5	5	3
0	0	0	0	4	5	5	5	4	5
0	0	0	0	5	5	5	5	5	5
0	0	0	0	4	4	5	5	5	4
0	0	1	0	4	4	4	4	3	5
0	1	0	0	1	2	3	3	1	5
0	1	1	0	4	4	3	2	2	4
0	0	0	0	3	5	4	4	4	5
0	0	1	1	4	3	3	3	4	3
0	0	0	0	4	5	5	3	4	4
0	1	0	0	4	4	4	2	3	4
0	0	0	0	5	5	5	5	5	4
0	0	0	0	3	4	5	3	4	4
1	0	0	0	4	3	4	4	4	5
0	0	0	1	4	4	2	2	3	2
0	1	0	0	4	4	4	4	3	3
1	0	0	0	2	5	4	4	4	5
1	0	1	1	3	4	4	4	4	4
0	0	0	1	3	3	5	3	4	3
0	0	1	0	4	5	5	1	4	5
0	1	0	0	4	4	5	5	5	5
1	0	1	1	4	5	3	2	4	2
0	0	0	0	4	4	5	5	4	4
0	0	0	0	5	5	4	3	4	5

0	0	0	0	3	5	5	5	5	5
1	1	1	1	2	2	3	2	3	4
0	0	0	0	5	5	4	2	4	4
0	1	0	0	5	5	4	3	2	2
0	0	0	0	5	5	5	4	4	5
0	0	1	1	4	5	2	2	1	4
1	0	0	0	5	5	5	5	4	5
0	0	0	0	4	4	4	5	5	3
0	0	0	0	3	4	5	4	4	5
0	1	0	0	4	4	4	2	4	2
0	0	0	0	4	4	4	3	3	5
0	0	0	0	5	5	5	4	4	5
1	1	0	1	3	3	4	4	4	3
1	0	0	0	4	4	3	4	4	5
0	0	0	0	3	4	4	1	4	5
0	0	0	0	4	4	5	3	4	3
0	1	0	0	3	4	4	3	3	3
0	0	0	1	4	3	4	3	4	4
0	0	0	0	4	4	4	2	2	4
1	0	0	0	5	5	5	5	3	5
1	0	0	0	5	5	4	1	3	5
0	0	0	0	5	3	4	5	5	2
0	0	0	0	5	5	5	5	5	5
0	0	0	0	3	4	4	3	4	4
0	0	0	1	4	4	4	3	4	5
0	0	0	1	5	2	4	4	3	4
0	0	0	0	5	5	4	4	4	2
0	0	0	0	5	5	4	4	4	2
0	0	0	1	4	4	4	4	4	3
0	0	0	0	5	5	5	3	4	4
0	0	0	0	2	4	4	4	4	4
0	0	0	0	3	3	4	4	5	5
0	0	0	0	3	3	4	3	2	5
0	1	0	0	3	4	4	4	4	5
0	0	0	0	5	5	4	4	4	5
1	1	0	1	3	3	2	2	2	5
1	0	1	1	3	3	4	2	4	4
0	0	0	0	5	5	5	5	5	5
0	0	0	0	4	4	5	5	4	5
0	1	0	0	4	5	5	5	5	5
0	1	1	0	4	4	4	2	4	2
0	0	0	0	4	5	5	5	5	4
0	0	0	0	5	5	4	4	4	4
1	0	0	0	2	5	4	3	4	3
0	0	0	1	3	5	4	3	2	4
0	0	0	0	4	4	3	2	2	3
0	0	1	1	4	5	4	2	4	4

0	0	0	0	5	5	4	4	4	5
0	0	0	0	5	5	2	2	4	4
0	0	0	0	4	4	5	4	4	5
0	0	0	0	4	5	4	4	3	2
0	0	0	0	5	5	5	4	1	5
0	0	0	0	5	5	5	5	5	5
0	0	0	0	5	5	5	5	5	5
0	1	0	0	4	3	4	3	3	4
1	1	1	1	3	4	3	3	3	5
1	1	1	1	2	3	4	2	4	5
0	0	1	1	5	5	4	4	4	5
0	0	1	0	5	5	5	5	1	2
0	0	0	0	5	5	5	3	5	4
0	0	0	0	5	5	4	4	4	5
0	0	0	0	5	5	4	4	4	4
1	0	0	0	3	2	2	4	2	4
0	0	0	0	5	5	1	5	4	5
0	0	0	0	5	5	5	5	5	3
0	0	0	0	3	4	4	4	4	4
0	0	0	0	5	3	4	4	4	5
0	1	0	1	1	2	5	4	4	5
1	0	0	0	4	4	5	2	4	4
0	0	0	1	4	5	5	4	5	5
0	1	0	0	5	5	4	4	4	5
0	0	0	0	4	5	5	5	4	5
0	1	1	1	4	4	3	3	3	1
0	1	0	0	2	2	4	2	4	4
0	0	0	0	5	5	1	1	4	5
0	0	0	0	2	5	4	4	4	5
1	1	0	0	3	4	4	4	4	2
0	0	0	0	4	4	4	4	4	5
0	1	0	0	5	4	5	5	4	5
0	0	0	0	3	4	4	4	3	4
0	0	1	0	3	4	2	4	4	5
1	1	0	0	5	5	3	2	4	1
1	1	1	1	2	2	5	5	4	5
0	1	0	0	4	3	1	2	4	5
0	0	0	0	5	5	5	5	5	4
0	0	0	0	2	3	4	4	4	4
0	0	0	0	4	5	4	4	4	4
0	0	0	0	3	4	5	4	4	3
0	0	0	0	5	5	3	4	4	5
0	0	0	1	5	5	3	1	1	5
0	0	0	0	5	5	1	5	5	5
0	0	0	0	3	5	5	5	4	5
0	1	0	0	3	4	4	4	4	3
0	0	0	0	5	5	4	4	5	4

0	0	0	0	4	4	4	4	4	3
0	0	0	0	4	4	4	3	3	4
0	0	1	0	3	4	2	4	2	4
0	0	1	0	3	3	4	3	2	5
0	1	0	0	5	5	4	2	4	4
0	0	0	0	5	5	4	1	4	3
0	0	0	0	2	4	4	3	4	2
0	1	0	0	5	5	3	3	3	4
0	0	0	0	5	5	4	4	3	4
0	0	0	0	5	5	4	4	4	5
0	0	0	1	3	3	3	3	3	3
0	1	0	1	4	4	5	2	3	1
0	0	1	0	5	5	4	4	4	5
0	0	1	1	4	4	3	2	2	4
0	0	0	0	3	3	4	4	3	2
0	0	0	0	3	5	4	4	4	5
0	0	0	0	5	5	4	3	4	5
0	0	0	0	4	4	4	4	4	2
0	1	1	0	3	2	4	4	4	2
0	1	0	0	3	3	4	1	4	3
1	0	0	1	5	5	4	3	1	1
1	0	1	0	4	5	4	4	4	5
0	0	0	0	5	5	4	1	4	2
0	1	1	1	4	5	3	4	3	3
0	1	1	0	4	4	5	4	3	5
0	0	0	0	4	5	4	3	4	4
0	0	0	0	2	2	5	4	4	5
0	0	0	0	4	4	4	4	4	4
1	0	0	0	5	5	4	3	4	3
0	1	1	1	3	3	4	4	4	2
0	0	0	0	5	5	4	4	4	5
0	0	1	0	5	5	5	4	5	5
0	0	0	0	4	4	4	3	4	4
0	0	0	0	5	5	4	5	5	5
1	0	1	0	5	5	1	5	4	5
0	1	0	0	5	5	5	5	5	3
0	0	0	0	5	5	4	4	5	5
1	1	1	0	3	3	4	3	3	3
1	0	0	0	3	3	4	4	4	5
1	1	1	0	2	2	3	3	3	4
1	1	1	0	5	5	4	2	4	2
0	0	0	0	4	4	4	3	4	5
0	0	0	0	4	4	4	5	4	5
0	0	0	0	5	5	4	1	4	5
0	0	0	0	4	2	4	4	4	4
1	1	0	0	2	5	4	2	1	5
0	0	0	0	4	4	4	4	4	5

0	0	0	0	2	2	3	3	3	2
0	0	0	0	5	5	4	4	2	5
0	0	0	1	4	4	4	4	4	5
0	1	0	1	4	5	5	3	4	4
0	0	0	0	2	2	5	5	5	5
0	1	0	0	4	5	4	4	4	3
1	1	1	1	2	1	5	2	2	5
0	0	1	1	3	2	3	3	2	4
0	0	1	0	1	2	4	3	3	5
1	0	0	0	4	5	5	4	4	5
1	1	0	1	2	2	2	4	2	4
0	0	0	1	2	2	5	5	4	5
1	1	1	0	5	5	2	1	1	3
0	0	0	1	5	5	5	5	5	5
0	0	0	0	4	4	4	4	4	5
1	1	1	1	2	2	4	4	3	2
0	0	0	0	2	3	3	1	4	5
0	0	0	0	4	4	4	2	2	2
0	0	0	0	4	4	1	2	1	5
0	1	0	0	3	4	4	4	4	4
1	0	0	0	4	4	4	4	4	2
0	0	0	0	4	5	1	3	4	4
0	0	0	0	4	4	4	3	3	2
0	0	0	0	5	5	4	4	4	5
0	0	0	0	4	4	4	4	3	2
0	0	0	0	4	5	5	4	4	5
0	0	0	0	4	4	4	4	4	4
0	0	0	0	4	4	4	4	4	4
1	0	1	0	5	5	5	5	5	5
0	0	0	0	5	5	5	5	5	5
0	1	0	0	2	4	4	4	3	5
0	0	1	1	5	5	3	1	3	5
0	1	1	1	5	3	3	3	4	2
0	0	0	0	5	2	4	5	5	5
1	0	0	0	4	4	4	4	4	4
0	0	0	0	3	3	4	4	4	2
1	0	0	0	4	4	4	4	4	2
1	0	0	0	4	5	4	4	5	1
0	0	0	0	3	3	4	3	3	3
0	0	0	0	4	4	1	1	3	4
0	0	0	0	4	4	4	5	4	4
0	0	0	0	3	3	4	4	4	4
0	0	0	0	5	5	1	1	1	5
0	0	0	0	2	2	4	5	5	5
0	0	0	0	4	5	5	5	5	2
0	0	0	0	3	5	5	5	5	5
0	0	0	0	4	4	3	1	4	5

0	1	0	0	5	5	3	4	4	1
0	0	0	1	5	5	4	4	4	4
0	0	0	0	4	4	5	5	5	5
1	0	0	1	3	3	4	3	4	4
0	0	0	0	5	5	5	5	5	5
0	1	1	1	3	2	5	3	3	4
1	1	0	1	3	4	5	2	5	3
0	0	0	0	4	3	4	3	3	3
0	0	0	0	3	2	4	4	4	4
0	0	0	0	3	5	5	1	4	5
0	0	0	0	4	5	4	4	4	4
0	0	0	0	5	5	4	4	4	5
0	0	0	0	5	5	4	5	4	3
0	1	0	0	4	3	4	4	4	4
0	1	0	0	4	4	3	3	4	3
1	1	1	1	4	4	4	2	4	2
0	1	0	1	3	3	4	2	4	2
0	0	0	0	5	5	4	4	4	4
0	0	0	0	3	4	5	4	4	3
0	0	0	0	5	5	5	5	5	5
0	0	0	0	4	5	4	5	4	5
0	0	0	0	4	4	4	4	3	4
1	0	0	0	2	2	4	4	4	4
0	0	0	0	5	5	4	3	4	4
0	0	0	1	5	4	5	2	1	5
0	0	0	0	5	5	5	1	5	5
0	0	0	0	4	4	5	4	4	2
1	1	1	1	3	3	4	2	3	4
0	1	0	0	5	5	4	4	4	4
0	1	0	0	3	3	4	3	3	3
0	0	0	0	2	2	3	2	4	2
1	1	0	0	2	5	5	3	4	5
1	1	1	1	4	2	4	1	4	2
0	0	0	0	5	5	4	4	4	3
0	0	1	0	5	5	5	5	4	5
0	0	0	0	5	5	5	4	4	5
0	0	0	0	5	5	4	3	4	2
0	0	0	0	3	5	4	3	4	2
0	0	0	0	4	4	4	4	2	5
0	1	0	0	3	4	5	4	5	4
0	0	0	0	5	5	5	5	5	5
0	0	0	1	5	5	4	5	4	5
0	0	0	1	4	3	4	4	4	3
0	0	0	0	4	5	4	4	4	5
0	0	0	0	4	4	1	5	4	3
0	0	0	0	3	2	4	3	4	1
1	0	1	0	5	5	5	5	3	2

0	0	0	0	4	3	5	5	5	4
0	0	0	0	5	5	5	4	4	4
0	0	0	0	3	4	4	4	4	5
0	1	0	0	3	4	5	3	4	3
0	0	0	0	4	4	4	3	4	4
1	0	0	0	3	3	5	5	5	4
1	1	0	0	3	3	4	4	3	4
0	0	1	1	2	3	4	4	2	4
0	0	0	0	5	5	5	3	3	5
0	0	0	0	4	4	5	5	4	4
0	0	0	0	5	5	5	2	4	5
0	0	0	1	3	4	3	4	2	5
0	0	0	0	5	5	5	5	5	5
0	0	0	1	4	5	4	4	4	5
0	0	0	0	4	4	4	4	4	5
0	0	0	0	5	5	4	4	4	4
0	0	0	0	4	5	1	4	4	4
0	1	0	0	4	3	3	2	3	2
1	0	0	1	5	4	3	3	2	5
0	0	0	0	4	4	4	4	4	4
0	0	0	0	3	2	5	5	5	5
0	0	0	0	5	5	4	4	4	4
0	0	0	0	4	5	5	5	4	4
0	0	0	0	2	4	4	3	4	5
0	1	1	0	4	4	4	3	2	4
0	0	0	0	4	4	4	4	4	4
1	1	1	1	2	2	2	2	2	5
0	0	0	0	4	5	5	4	4	5
0	1	0	0	3	3	4	4	4	5
1	0	0	0	5	5	3	4	4	4
0	0	0	0	5	5	4	4	4	5
0	1	0	0	3	3	4	4	4	4
0	0	0	0	2	2	3	2	4	5
0	0	0	0	5	5	4	4	4	5
0	0	0	0	5	5	4	5	5	5
0	0	0	0	4	3	4	4	4	4
0	0	1	0	5	5	3	5	4	4
0	0	0	1	5	5	4	3	4	2
0	0	0	0	5	5	5	3	4	5
0	0	0	0	5	5	4	4	4	3
0	0	0	1	5	5	4	5	3	4
0	1	1	0	3	3	4	3	3	3
0	0	0	0	4	4	4	3	4	4
0	0	0	0	4	4	4	4	4	5
0	1	1	1	5	4	5	3	4	4
0	0	0	0	4	5	4	1	2	5
0	0	1	1	2	4	3	2	2	2

0	0	0	0	5	5	4	4	4	5
0	0	0	0	2	3	3	3	3	4
1	1	1	1	4	4	2	2	2	4
0	0	0	1	5	5	3	1	4	5
0	1	1	0	5	4	3	2	1	2
0	1	0	0	3	4	4	3	4	5
1	1	1	0	2	2	2	2	2	2
1	0	0	0	5	4	1	5	5	4
0	0	0	0	4	2	3	4	4	5
0	0	0	0	5	5	5	3	5	3
0	0	0	0	4	5	4	3	4	3
0	0	0	0	4	5	4	4	4	5
0	1	0	0	5	5	4	2	4	5
0	1	1	1	4	5	3	2	3	2
0	0	0	0	4	4	4	3	3	5
0	0	0	0	4	4	3	2	4	4
0	0	0	0	5	5	5	2	4	3
0	0	0	0	3	5	5	4	5	3
0	1	1	0	5	5	4	4	3	4
0	0	0	1	3	3	4	4	4	5
0	0	0	0	4	4	1	5	4	5
0	0	0	0	5	4	4	4	3	5
1	0	1	1	4	5	2	1	2	2
1	1	0	1	2	2	4	4	4	2
0	0	0	0	5	5	5	4	3	4
0	0	0	0	4	4	4	4	3	4
0	0	0	0	5	5	4	4	4	5
0	0	0	0	3	3	4	3	4	4
0	0	0	0	5	5	5	5	5	5
0	1	1	0	5	5	3	3	3	4
0	0	0	0	5	5	5	3	4	4
0	0	0	0	3	3	4	3	4	4
0	1	0	1	3	2	4	3	4	2

old_7	old_8	old_9	old_10	old_11	old_12	old_13	old_14	old_15	old_16
4	5	2	5	5	5	3	4	5	4
4	4	2	5	3	4	4	3	4	4
5	5	4	3	3	2	1	3	2	2
5	4	4	4	4	4	4	4	4	4
1	1	1	4	4	2	3	4	4	4
5	5	4	5	5	5	5	5	5	5
3	3	3	2	3	3	3	3	4	3
4	2	2	2	3	3	2	2	2	3
4	5	3	5	1	4	1	5	4	5
1	1	1	5	4	4	3	4	3	3
4	3	2	4	4	4	2	4	4	4
2	2	2	3	3	4	4	4	4	4
3	2	2	4	3	3	4	4	5	4
3	2	2	4	4	3	2	3	4	4
4	4	4	5	4	4	4	4	4	4
5	5	4	4	4	4	4	4	5	4
2	3	1	4	4	4	3	4	4	4
5	4	2	4	4	4	4	3	4	4
2	2	2	5	4	4	4	3	4	4
5	5	5	5	4	4	4	4	5	4
2	4	4	4	2	4	4	4	5	4
2	2	2	5	2	4	2	4	4	4
5	4	4	5	4	4	4	4	5	4
4	4	4	4	4	4	2	2	3	3
4	4	4	4	3	3	3	3	3	3
3	5	2	5	4	4	5	4	5	5
4	4	4	4	3	4	3	3	4	4
2	2	1	5	2	1	2	2	1	2
4	4	2	5	3	2	2	4	4	2
3	2	2	3	3	3	3	3	4	3
3	2	2	3	3	3	3	3	4	3
5	5	5	5	4	4	4	4	4	4
2	2	2	2	3	3	3	4	4	3
5	4	4	5	4	5	5	5	5	4
4	4	4	4	3	4	3	4	5	5
1	1	2	3	3	3	3	3	3	3
1	2	2	2	4	5	5	5	3	2
5	5	5	5	3	4	4	3	5	4
3	5	1	5	5	5	5	5	5	4
2	2	1	4	4	3	2	3	2	3
3	3	2	2	4	4	4	4	4	4
4	4	4	5	5	5	5	5	5	5
4	4	2	3	4	2	2	3	2	3
5	3	2	4	4	4	3	3	4	4
5	5	5	5	4	4	5	5	5	5
3	3	2	4	3	3	4	4	4	4

4	4	2	2	3	3	3	4	4	4
3	3	2	3	3	3	3	3	4	4
4	4	3	2	5	3	3	3	1	4
4	3	2	5	4	4	4	4	4	4
2	2	2	5	2	2	2	2	2	2
5	5	5	2	4	4	5	4	5	4
4	5	5	5	2	3	2	2	3	3
2	2	2	3	4	4	4	4	3	1
5	5	1	5	2	4	4	4	5	4
5	5	5	5	4	4	4	4	5	5
2	2	1	4	3	2	4	4	3	4
4	4	4	2	4	4	4	4	4	4
2	2	2	1	5	4	2	4	1	1
5	2	2	2	4	5	3	2	5	1
4	4	1	3	3	3	3	4	1	1
4	4	1	3	3	3	3	4	1	1
3	5	3	1	4	5	5	5	5	4
5	4	4	4	4	4	3	4	5	4
5	3	2	4	4	3	4	4	4	4
5	5	3	5	4	4	4	4	5	5
4	4	3	5	3	4	2	2	4	4
5	5	3	5	2	2	2	3	2	3
5	5	2	3	4	2	2	1	4	4
5	5	5	5	1	4	4	4	1	1
5	5	5	2	3	2	3	3	4	4
5	5	4	5	1	1	2	2	3	1
5	2	5	2	4	4	3	4	4	4
5	5	5	4	3	3	2	4	3	4
5	5	5	5	4	4	4	4	5	4
3	3	2	5	4	3	4	4	4	1
5	2	5	5	4	4	4	3	4	3
2	2	3	4	3	4	4	3	5	5
5	5	5	4	2	4	4	1	4	1
2	2	2	4	4	4	4	4	5	4
2	3	2	5	3	2	3	2	3	3
3	3	2	4	3	2	1	2	2	3
5	5	3	4	4	3	2	2	2	4
5	5	5	1	5	5	5	5	5	5
1	1	1	5	4	4	3	4	4	4
2	3	3	4	4	4	4	4	4	4
5	5	1	5	3	3	3	4	5	4
3	4	3	5	4	4	4	4	5	5
4	4	2	5	4	3	2	4	4	4
2	2	1	1	4	4	4	5	4	4
5	5	5	4	4	4	4	4	5	4
5	5	2	5	4	4	4	4	4	5
5	5	5	5	5	4	4	4	5	5

5	2	2	4	2	2	4	2	4	2
5	5	2	5	5	5	4	5	5	5
5	5	1	3	4	4	3	4	4	3
4	5	4	4	4	4	5	4	5	5
2	2	2	4	4	4	2	4	2	3
4	2	1	5	4	3	1	3	5	3
4	5	3	5	1	4	2	3	3	3
4	5	3	5	3	4	1	3	4	4
5	5	5	5	5	5	5	5	5	5
2	2	2	3	4	2	3	4	4	4
4	4	4	5	4	4	3	4	4	4
4	4	5	4	4	2	2	3	4	3
5	5	5	5	5	5	4	5	5	5
5	4	4	5	4	4	4	4	5	4
5	5	5	5	4	5	5	5	5	5
1	1	1	4	2	3	2	3	3	3
2	2	2	2	4	4	2	2	4	3
2	2	2	4	3	4	4	3	5	5
3	2	2	5	4	3	3	5	4	4
4	4	2	5	4	4	1	4	5	5
4	2	2	3	4	4	3	3	5	5
5	4	4	5	4	4	2	4	4	1
3	5	5	1	2	2	4	2	5	4
2	2	2	5	4	4	4	4	1	1
5	3	5	5	4	3	3	4	4	1
5	4	5	5	2	4	4	4	5	5
5	4	2	5	2	3	2	3	4	2
5	5	5	5	1	1	3	3	5	2
4	5	2	3	3	3	3	3	3	4
5	2	5	4	4	4	4	4	5	3
4	4	1	5	4	5	4	4	5	1
4	5	5	5	1	1	4	3	4	5
3	2	2	2	3	4	4	4	5	3
3	3	3	3	3	3	3	3	4	4
4	2	4	5	3	2	2	3	3	2
5	3	1	5	5	5	5	4	5	5
5	5	2	2	4	4	3	4	1	1
3	3	1	4	2	2	2	2	3	1
2	2	5	5	4	4	2	4	5	5
5	5	2	5	3	3	3	3	4	4
5	5	2	5	4	4	4	4	4	4
5	5	2	4	4	4	4	4	1	1
4	5	2	4	4	4	4	4	5	4
2	2	2	4	2	1	1	2	2	2
5	5	3	5	4	3	2	3	3	4
5	5	1	5	5	5	3	4	5	4
5	5	2	5	4	4	4	4	5	5

5	5	3	5	4	4	4	4	4	3
2	4	2	5	2	2	4	4	4	4
2	2	1	1	4	2	3	2	2	2
4	4	2	2	4	3	4	4	4	4
1	1	2	5	3	1	1	3	2	1
5	5	2	4	3	3	4	4	4	4
5	5	1	5	4	4	2	4	5	5
5	5	1	5	2	2	2	2	1	3
4	4	1	4	4	3	3	4	5	4
3	4	1	5	5	5	5	5	5	5
5	5	2	5	4	4	4	4	4	5
1	3	1	5	1	1	1	1	1	1
2	2	2	4	3	3	3	4	4	4
3	5	5	2	5	4	3	4	3	4
2	2	2	5	3	3	3	3	5	4
5	5	5	5	4	4	4	1	4	3
5	5	5	5	4	1	2	1	1	4
5	5	4	5	4	2	2	2	3	2
5	5	4	5	4	4	4	4	4	4
4	4	5	4	4	4	4	4	4	5
5	5	2	5	4	4	4	4	4	4
2	3	2	4	4	4	4	4	4	4
4	4	2	3	4	4	4	3	3	4
3	3	3	5	3	3	4	4	4	4
5	3	5	4	2	4	4	5	5	4
5	5	4	4	3	4	2	4	5	5
2	2	2	4	3	3	3	3	2	2
4	5	4	4	4	4	4	4	4	4
1	2	1	5	4	3	1	4	4	4
5	5	1	5	4	3	2	4	5	4
3	4	3	2	4	3	5	3	3	3
5	5	2	5	4	4	4	4	5	4
4	4	2	5	4	4	4	4	3	4
2	2	2	5	4	4	3	4	4	4
5	4	4	3	4	4	3	3	4	4
2	1	1	3	3	3	2	4	2	4
2	2	2	3	3	4	4	4	5	5
5	5	1	5	4	4	4	4	4	4
5	5	2	4	1	1	4	4	1	1
5	5	4	5	5	5	5	3	4	4
5	4	5	5	1	4	4	4	4	4
5	5	4	5	5	5	3	4	3	3
2	2	1	4	3	4	4	3	5	5
5	5	5	5	5	5	4	5	5	4
5	5	4	5	4	4	4	4	3	4
4	5	3	4	3	4	4	4	1	1
5	5	1	5	3	3	3	3	3	4

5	5	2	5	4	4	3	2	2	2
5	5	4	5	3	4	3	2	4	3
5	5	4	5	4	2	4	3	5	4
5	4	2	5	4	2	2	3	2	2
5	5	2	4	4	4	3	4	1	1
1	5	1	2	4	5	3	2	3	4
2	4	2	3	4	3	3	3	4	4
2	1	2	5	4	4	4	4	4	4
5	5	4	5	5	5	5	5	5	5
5	5	4	4	4	4	4	5	5	5
2	3	2	3	3	2	2	2	3	4
2	2	2	4	4	4	4	4	4	4
4	4	2	5	4	4	4	3	5	5
4	4	4	3	4	4	4	4	5	4
5	5	2	5	4	3	4	4	5	1
4	4	2	5	4	4	2	3	1	1
4	4	2	4	4	3	2	4	3	3
5	5	2	5	4	5	5	5	5	5
4	2	1	3	3	3	3	4	3	2
2	4	1	5	4	2	3	5	2	3
3	3	2	4	3	3	4	4	4	4
4	4	4	5	4	4	3	4	4	4
4	5	3	4	4	4	3	3	4	4
4	4	2	4	3	4	2	3	4	3
5	5	5	5	5	5	5	5	5	5
1	2	1	4	3	4	3	4	4	4
4	5	4	4	4	4	3	4	4	4
4	5	4	5	4	4	5	4	5	5
2	2	2	4	3	3	3	3	3	3
5	5	1	5	4	4	4	4	4	4
3	3	2	4	4	4	3	3	4	4
4	4	4	4	3	3	3	3	1	3
2	2	1	4	4	3	4	3	3	4
4	4	4	5	4	4	4	4	5	4
5	5	4	5	3	2	4	4	3	2
2	2	1	5	5	5	5	5	5	4
1	1	1	3	4	4	3	3	3	3
4	3	2	5	2	2	1	2	3	4
5	5	4	5	2	1	4	3	1	2
5	5	2	5	4	4	4	4	4	4
5	4	4	5	4	4	4	4	5	5
5	5	2	5	2	3	2	2	3	2
2	1	1	4	3	4	1	3	3	3
2	3	2	3	4	4	3	3	4	4
5	5	5	3	3	3	3	4	4	4
5	5	5	5	5	5	4	4	5	5
4	5	1	5	4	3	3	4	3	4

4	4	4	4	2	2	2	2	3	3
5	5	2	5	1	4	3	4	4	5
5	5	3	5	4	4	4	4	5	5
2	2	2	5	4	3	2	3	4	3
5	5	2	5	5	4	5	5	5	5
5	5	5	5	1	1	1	1	4	1
5	3	2	5	4	3	4	4	4	3
5	5	2	4	4	4	3	3	3	4
5	5	1	5	5	4	4	4	5	5
3	3	2	4	3	2	2	2	3	3
5	5	2	5	4	4	3	3	3	3
4	4	2	4	4	4	3	2	3	4
2	2	1	5	4	2	1	3	4	2
3	5	3	4	3	3	4	3	3	3
5	5	3	2	3	3	4	4	3	3
3	4	2	5	4	4	4	4	5	4
2	2	2	5	4	4	4	4	3	3
4	3	2	4	2	2	2	4	4	3
5	5	5	5	3	3	3	4	4	4
5	5	2	5	4	4	4	4	3	3
5	4	3	4	3	3	3	3	2	3
5	5	2	4	4	4	4	4	4	4
2	2	2	4	4	4	4	4	4	4
1	3	5	5	4	4	4	4	5	5
5	5	4	5	1	4	5	5	5	5
5	5	5	5	3	4	5	5	5	5
4	4	4	4	2	4	5	4	5	3
5	5	2	5	4	4	4	4	4	4
5	5	3	3	1	1	1	4	2	1
4	2	2	3	2	2	2	2	3	3
5	5	5	5	1	4	4	1	4	4
3	3	3	3	3	3	3	3	3	3
1	5	2	5	4	4	4	3	4	4
3	4	2	4	4	2	2	3	4	3
4	5	4	5	4	4	4	4	5	4
5	5	5	4	2	3	3	2	3	3
5	5	4	4	4	5	2	3	5	4
2	4	4	3	3	2	2	2	3	3
2	4	2	4	2	3	2	3	4	3
4	4	2	5	4	5	4	4	5	5
1	2	1	4	4	4	2	2	4	4
1	1	1	3	4	3	3	3	4	4
5	5	5	5	4	4	3	4	3	4
5	5	5	5	4	3	3	4	3	3
4	4	2	5	2	3	2	3	3	2
5	4	4	5	5	2	4	3	4	4
4	4	2	5	4	4	4	4	4	4

5	5	2	5	5	5	5	5	5	5
4	4	2	3	2	2	2	3	4	2
4	5	2	5	4	4	3	4	4	3
2	2	2	5	3	1	2	3	3	2
5	5	5	5	4	4	5	4	5	5
4	5	2	4	2	1	1	1	2	2
5	5	5	5	1	5	1	1	5	5
2	2	2	5	4	4	4	3	4	4
4	5	1	3	4	2	2	2	4	4
2	2	2	5	2	2	3	3	2	3
5	4	2	3	3	3	3	4	4	4
5	5	2	5	4	5	4	4	5	5
4	4	4	4	3	4	4	3	4	4
2	3	2	4	4	4	3	4	3	4
5	5	5	5	4	4	2	4	4	5
2	2	4	4	2	2	3	3	3	3
3	4	3	4	3	3	3	3	3	3
5	3	3	3	3	4	3	3	4	4
4	3	4	5	4	4	3	3	5	5
5	5	5	5	3	3	4	3	2	4
4	5	3	4	3	3	2	3	5	5
1	2	1	5	3	4	4	3	5	4
5	5	5	5	4	4	4	4	5	4
5	4	2	5	2	3	2	2	4	4
4	3	2	4	4	4	3	3	4	3
5	4	2	4	3	4	3	2	4	4
2	1	1	5	5	5	4	5	4	3
2	1	1	5	5	5	4	5	4	3
3	3	2	4	4	4	4	4	4	4
2	2	1	5	3	3	3	4	3	3
2	2	2	2	4	4	3	4	5	4
5	5	5	4	4	4	4	4	5	5
5	4	2	3	5	4	3	4	4	4
4	5	4	4	4	3	4	4	3	3
5	5	5	5	4	4	4	4	5	5
4	5	2	4	2	2	1	1	2	2
3	5	2	4	4	1	1	1	1	2
5	5	2	5	4	4	4	4	5	5
4	4	3	4	4	3	4	3	3	1
5	5	4	4	4	4	3	3	3	3
2	1	1	5	2	3	4	4	3	3
3	5	3	5	5	4	3	4	4	4
4	4	2	5	4	4	4	4	5	5
2	2	2	5	4	4	3	3	4	4
5	5	5	4	2	2	4	2	1	1
4	2	1	4	3	4	3	2	3	3
2	4	2	3	3	4	2	3	1	2

5	5	3	5	4	4	4	4	4	4
4	4	4	5	4	4	4	4	4	4
5	5	5	4	5	5	4	5	4	4
2	1	1	5	4	4	4	4	5	3
5	3	3	5	5	3	3	3	5	4
5	5	5	5	5	5	5	5	5	5
5	5	4	5	5	5	4	5	5	5
4	4	3	5	4	3	3	3	3	3
5	5	5	4	2	1	1	2	1	3
5	5	1	4	3	3	2	3	4	2
2	5	2	5	4	2	2	1	3	2
2	1	1	5	4	5	1	4	4	4
4	4	1	5	5	5	4	4	5	5
5	5	5	5	4	4	4	4	4	4
4	5	3	4	4	4	4	4	5	3
5	4	3	4	3	4	3	2	1	3
4	5	5	5	4	5	5	5	4	1
3	3	2	5	4	3	3	4	4	4
5	5	2	4	4	4	4	4	1	3
4	5	4	4	4	4	4	5	5	5
2	5	2	5	4	4	4	4	5	5
5	5	2	5	4	3	4	4	3	2
5	5	1	2	3	3	3	4	5	5
5	4	4	5	3	3	3	4	4	2
4	5	5	5	4	4	5	4	4	5
1	1	1	4	4	4	2	3	3	2
5	5	2	1	3	3	2	2	2	2
4	4	3	5	1	1	4	4	1	4
5	5	2	4	4	4	4	4	4	5
2	3	2	4	3	4	3	3	3	3
5	5	2	4	2	4	4	4	4	4
5	5	2	5	3	3	1	2	2	3
4	3	2	4	4	4	4	4	4	4
5	5	4	4	2	4	3	4	4	4
1	5	4	4	3	2	2	3	2	2
5	5	2	2	3	1	1	2	4	3
5	5	3	5	4	4	2	5	5	4
5	5	5	5	4	4	4	4	5	5
4	4	2	3	4	4	4	4	4	4
4	4	4	5	4	4	4	4	5	5
3	2	2	4	3	4	3	4	4	4
5	5	4	4	4	5	4	5	5	5
5	5	5	5	2	1	1	1	2	2
5	5	3	5	5	5	4	4	4	5
5	5	2	5	3	5	4	4	4	4
4	3	1	4	3	4	2	4	3	4
5	3	2	5	4	4	4	4	5	4

3	3	2	4	2	3	2	2	4	4
4	5	2	4	3	4	4	4	5	5
4	3	3	4	1	2	2	3	1	1
5	5	2	2	3	3	3	4	5	5
4	3	2	5	4	4	4	4	3	3
4	5	1	5	4	2	4	4	5	4
2	2	2	3	4	4	4	4	4	4
2	3	3	4	3	4	4	3	3	3
4	4	4	5	4	3	3	3	4	2
5	4	2	5	5	4	4	4	5	5
3	2	1	3	3	4	3	2	4	3
2	4	2	5	4	1	2	2	3	3
5	5	5	5	4	4	4	4	4	4
3	3	2	4	4	4	2	3	4	3
2	2	2	4	3	4	3	4	4	4
5	5	1	3	3	3	4	3	4	3
4	5	5	5	1	4	3	3	4	4
2	1	5	5	2	2	4	4	4	4
3	5	3	3	2	3	1	2	1	3
2	2	5	2	2	2	4	2	5	4
2	2	2	5	3	4	2	2	4	3
3	5	2	4	3	3	3	2	3	4
1	1	1	5	3	4	4	4	4	4
3	3	2	4	3	2	2	2	4	4
5	5	3	3	4	2	3	1	1	2
4	3	4	5	3	3	3	3	4	4
5	4	1	1	5	4	4	5	5	5
3	2	2	5	4	4	4	4	4	4
2	2	3	3	4	3	4	4	5	4
2	2	2	2	4	2	2	2	2	2
4	5	4	5	4	5	4	4	5	5
5	5	3	5	4	4	4	4	5	5
4	3	2	5	4	4	3	3	4	4
5	5	5	5	5	5	5	5	5	5
5	5	4	5	5	4	4	4	5	5
2	3	2	5	4	4	4	4	5	4
5	5	3	5	5	4	2	4	4	4
2	3	2	2	3	3	3	3	3	3
5	5	4	4	4	4	4	4	4	4
5	4	2	2	4	4	4	2	5	2
2	2	2	5	4	2	3	4	3	2
5	5	2	4	3	4	2	4	5	4
5	5	2	5	5	4	5	4	3	5
3	2	2	5	2	1	2	3	4	4
4	5	4	4	4	3	3	4	4	4
5	5	2	5	5	4	4	4	1	3
5	5	5	5	4	4	3	2	3	3

1	2	2	1	3	3	3	4	1	5
5	5	3	5	4	4	4	4	4	4
5	5	2	5	3	3	2	4	3	4
4	4	3	3	3	2	2	2	2	2
5	5	2	5	5	5	5	5	5	5
3	4	2	5	3	3	4	3	3	2
4	1	1	1	2	2	1	1	1	1
3	2	5	4	1	2	2	2	2	4
5	5	5	2	4	1	3	3	4	4
4	4	2	5	4	4	4	4	4	4
5	2	2	2	1	2	4	1	2	2
5	5	5	2	5	5	4	5	5	5
2	5	2	5	1	1	3	2	1	3
5	5	1	5	5	5	4	4	4	5
5	5	5	5	4	4	4	4	5	5
2	2	2	2	3	4	3	3	3	3
5	5	2	3	4	4	2	3	5	1
2	2	2	4	3	2	3	2	3	3
5	5	5	5	1	5	4	5	5	5
2	2	1	5	3	3	4	4	4	2
2	2	3	4	4	4	4	4	4	4
5	5	5	5	2	4	1	2	4	4
3	3	2	4	4	4	4	2	4	3
5	5	5	5	4	4	3	4	4	4
2	2	2	4	4	4	4	4	4	4
5	3	2	5	5	4	5	4	5	5
4	4	2	4	4	4	3	4	4	4
4	4	2	4	4	4	4	4	4	4
5	5	4	5	5	5	4	5	4	5
5	5	4	5	5	4	4	5	4	4
5	5	1	3	4	4	4	4	4	4
1	1	5	5	1	2	5	4	1	4
2	2	2	3	3	3	4	3	4	3
5	5	5	2	4	4	2	2	1	1
5	4	3	5	4	4	4	4	4	4
2	2	2	3	3	3	4	4	2	4
2	2	1	4	4	4	4	4	5	4
3	5	4	1	4	5	5	5	5	4
2	3	2	3	4	3	3	3	4	3
4	4	3	4	4	3	3	4	4	3
3	2	2	2	4	4	4	4	4	4
5	3	1	2	4	1	5	3	4	1
5	5	5	5	1	1	1	1	4	4
1	5	2	2	3	3	2	4	3	4
2	2	2	5	5	4	4	4	4	4
5	5	3	4	1	1	4	2	1	4
5	5	3	4	3	2	2	3	4	3

1	2	3	5	3	4	3	3	4	4
5	5	1	3	2	2	2	2	5	4
5	5	5	5	5	5	5	4	5	5
3	4	2	4	3	3	4	4	3	3
5	5	5	5	5	5	4	5	5	5
5	4	3	5	3	2	2	2	3	3
2	3	1	5	2	2	2	3	4	3
2	4	2	4	3	3	3	3	3	4
3	3	5	3	4	4	4	4	5	4
4	5	1	4	4	5	3	3	4	4
5	5	3	4	4	4	3	4	5	5
5	5	4	5	4	5	5	4	5	5
3	3	5	5	4	4	4	4	5	4
4	3	2	4	3	3	3	3	4	3
3	3	3	4	3	4	3	3	4	4
4	5	2	4	2	1	2	2	1	1
3	3	3	4	4	4	2	3	2	2
5	5	5	5	4	4	4	4	5	5
3	4	3	4	4	4	4	4	5	4
5	5	1	5	4	4	4	4	5	5
5	5	3	4	4	4	5	4	4	4
3	3	5	4	4	3	2	3	3	4
3	3	2	2	4	5	4	4	4	5
4	3	2	4	4	4	4	4	1	1
5	5	3	3	4	4	1	1	1	5
5	5	1	5	4	5	4	4	5	5
2	3	2	4	4	4	4	4	5	5
4	5	2	4	4	2	2	3	2	2
3	3	2	5	4	3	2	2	4	3
3	3	2	3	3	3	2	2	4	3
2	3	2	3	3	3	3	3	4	4
5	5	1	4	4	4	4	3	4	3
2	1	2	2	3	2	4	4	5	4
2	3	2	5	4	4	4	4	4	4
5	5	5	5	5	4	5	5	5	4
5	5	2	5	5	5	5	5	5	5
2	2	2	5	4	3	4	4	4	4
3	2	2	5	3	3	3	3	3	4
4	5	2	4	4	4	4	4	5	5
5	5	2	5	2	4	4	3	4	4
3	2	1	5	5	4	4	3	4	4
5	5	5	4	3	5	3	3	4	3
2	2	2	4	2	3	2	4	2	3
5	2	2	4	4	4	5	5	5	5
3	3	3	4	4	2	4	4	5	3
1	1	1	3	3	3	2	3	4	4
2	2	4	5	1	2	3	4	4	4

3	2	1	5	4	4	3	3	3	4
4	2	2	5	4	4	4	4	4	4
5	5	2	4	4	4	4	4	4	4
3	2	2	4	4	4	3	3	4	4
4	2	1	4	4	4	4	4	4	4
4	4	4	4	5	5	4	5	5	5
4	4	3	3	3	3	2	3	3	3
4	2	1	3	2	3	2	3	3	3
5	5	5	5	3	4	2	2	4	3
4	4	2	3	4	4	2	4	3	3
5	5	2	5	4	4	4	4	3	4
4	4	2	4	2	2	2	3	4	4
5	5	2	5	5	5	5	5	5	5
5	5	4	4	3	4	4	3	1	3
4	5	4	2	3	3	4	4	4	3
5	5	2	5	4	4	4	4	4	4
5	5	2	2	2	5	4	4	5	5
2	2	3	4	3	4	2	3	4	3
5	5	1	3	4	4	4	4	5	5
5	3	3	4	4	4	4	4	4	5
5	5	5	3	5	5	5	5	5	5
5	5	4	5	3	3	4	4	5	5
5	4	2	5	4	4	3	3	4	4
4	5	2	2	4	5	4	4	5	4
4	4	4	4	3	2	3	2	3	2
4	4	4	4	4	4	4	4	4	4
5	5	1	2	2	2	1	1	3	2
5	5	5	5	4	4	3	4	5	4
5	5	2	3	4	4	4	4	4	4
4	3	1	1	4	5	5	5	5	5
5	5	5	5	4	4	4	4	5	5
4	4	4	4	4	4	4	4	4	4
5	5	5	2	4	4	4	2	5	5
5	5	4	5	5	4	5	4	5	5
5	5	5	5	4	4	4	4	5	4
4	4	2	4	4	4	4	4	4	4
5	3	3	2	3	4	4	4	5	4
2	2	2	5	4	4	3	2	4	4
4	4	3	5	3	4	4	4	5	5
3	3	3	5	4	4	4	4	5	4
4	2	1	5	3	4	4	3	4	4
3	3	3	3	3	4	3	3	4	4
4	3	2	5	4	4	4	2	4	3
5	4	4	5	4	4	4	4	1	4
4	5	3	5	3	4	4	4	5	4
5	5	4	5	4	3	1	3	5	4
3	3	1	5	3	1	2	4	1	3

5	5	5	5	4	4	4	4	4	4
3	3	2	3	3	3	3	3	4	4
3	4	4	5	4	2	2	2	2	2
5	1	1	5	2	2	4	2	3	3
2	3	4	5	2	1	2	2	2	2
3	3	3	4	3	2	4	3	1	1
2	1	1	2	2	2	3	2	3	2
4	3	3	5	4	5	5	5	2	3
5	5	5	5	4	4	4	4	5	5
3	3	2	2	5	4	4	4	4	4
4	4	2	5	4	4	4	4	4	4
5	5	3	5	4	4	4	4	4	4
5	5	3	5	3	2	3	2	4	3
2	2	2	5	4	4	2	3	4	4
5	5	4	5	4	4	3	2	3	2
4	5	2	4	4	3	3	4	4	4
3	2	2	5	3	2	4	3	3	3
4	3	2	4	4	2	4	3	4	4
3	3	2	4	3	2	2	2	2	3
5	5	4	4	4	4	4	4	1	5
5	4	4	5	4	5	5	5	5	5
5	5	2	5	4	4	4	4	3	4
4	5	2	5	2	1	2	2	2	2
4	5	2	2	3	1	2	4	2	3
4	4	1	5	4	4	2	3	3	3
5	2	2	4	4	4	3	4	4	4
5	5	2	4	4	4	2	3	4	4
4	4	2	3	4	3	4	3	4	5
5	5	2	5	5	5	4	5	5	5
4	4	3	5	1	1	3	3	4	4
5	5	4	4	5	5	4	4	5	4
4	4	3	3	4	3	3	3	4	4
2	2	2	2	4	4	3	3	3	3

old_17	old_18	old_19	old_20	old_21	old_22	old_23	old_24
4	3	5	4	3	4	4	2
5	4	5	4	4	5	5	4
2	3	2	4	3	3	3	3
4	4	4	4	4	4	4	4
3	2	3	4	2	3	2	2
5	5	5	5	5	5	5	5
3	3	4	3	3	3	3	3
3	2	2	2	2	2	2	2
5	4	3	3	4	4	4	4
3	3	4	4	5	4	4	4
4	4	4	3	4	4	4	4
4	4	4	4	4	4	4	4
4	4	4	4	4	4	4	4
3	4	4	3	3	3	3	2
4	5	4	5	4	5	5	5
5	4	4	4	4	4	4	4
4	3	4	4	4	4	4	4
3	4	4	4	4	4	4	4
4	4	4	4	4	4	4	4
4	3	4	4	4	4	4	4
4	3	3	4	4	4	4	4
4	4	4	4	4	4	4	4
4	4	4	4	4	4	4	4
3	3	4	4	3	3	3	3
3	3	4	4	3	3	3	3
4	4	4	4	4	4	5	4
4	4	4	4	4	4	4	4
2	2	3	1	2	1	2	2
4	2	3	4	4	4	4	3
3	3	3	4	3	3	3	3
3	3	3	4	3	3	3	3
4	4	4	5	4	4	4	3
3	2	3	2	3	4	3	3
4	5	4	5	4	4	4	4
5	3	4	4	4	4	3	4
2	2	3	3	4	5	5	5
2	4	1	2	2	4	4	5
1	1	4	5	4	4	4	4
4	4	5	5	5	5	5	5
3	5	3	4	3	4	2	2
3	4	3	3	4	4	3	3
5	5	5	5	5	5	5	5
2	2	2	2	2	2	2	2
4	4	4	4	4	4	4	4
5	3	5	5	5	5	3	3
4	4	4	4	3	4	4	3

4	4	3	3	3	3	2	3
4	4	3	3	3	3	3	3
4	4	4	4	4	4	3	3
4	4	4	4	4	4	4	4
4	2	2	4	3	4	1	1
4	4	5	5	4	4	4	4
4	4	4	4	2	2	4	4
4	4	4	3	4	4	4	4
4	3	4	4	5	5	5	4
5	5	5	5	4	4	4	4
3	3	4	3	4	5	5	4
4	4	4	4	4	4	4	4
1	4	2	4	4	4	4	4
1	1	1	5	5	5	5	5
4	1	5	4	4	4	5	3
4	1	5	4	4	4	5	3
5	4	4	4	4	4	4	4
4	4	4	4	4	4	4	4
4	2	4	4	3	4	4	3
5	5	4	4	5	5	5	5
3	3	4	4	4	4	4	4
4	3	3	4	2	3	3	3
3	2	2	5	2	3	3	1
4	4	4	4	4	4	4	4
3	3	3	2	4	3	3	3
1	3	1	3	2	3	4	4
3	4	4	4	4	4	4	4
4	3	3	3	4	4	4	4
4	4	5	4	4	4	4	4
4	4	4	5	4	4	4	4
3	3	1	5	4	4	4	4
4	4	4	4	4	4	4	4
1	1	5	4	1	4	4	4
4	4	4	4	4	4	4	4
4	3	3	4	4	3	3	3
2	2	2	4	3	3	2	2
4	1	5	2	4	4	4	3
5	5	5	5	5	5	5	5
4	3	4	4	4	4	4	4
4	4	4	4	4	4	4	4
3	2	3	4	2	5	3	3
5	5	4	4	4	5	4	4
3	4	3	3	4	4	4	2
5	5	4	4	4	5	4	5
4	5	4	4	4	4	4	4
4	4	5	5	5	5	4	4
5	5	5	5	4	5	4	4

4	4	4	4	4	4	4	4
5	3	4	4	4	5	5	5
3	3	4	3	2	2	1	1
5	5	5	4	5	5	5	5
4	4	3	5	2	2	2	1
3	2	3	3	3	3	3	2
3	3	3	4	2	3	3	2
4	4	4	4	2	3	3	3
5	5	5	5	4	5	5	5
4	3	3	4	4	4	3	4
4	4	5	4	4	5	4	4
3	3	4	4	4	4	5	5
5	5	4	5	5	5	5	5
5	4	5	5	4	5	4	4
5	5	5	5	5	5	5	5
1	4	4	4	4	4	4	4
2	2	4	3	4	4	4	3
4	4	5	4	4	5	4	4
4	4	4	4	4	3	1	2
4	4	5	5	4	5	4	4
5	5	5	3	4	4	4	4
4	4	3	4	4	3	4	2
3	3	4	1	1	4	2	2
1	4	2	3	4	4	4	3
4	3	4	5	4	4	4	4
1	2	1	4	4	4	4	4
3	3	3	4	3	4	2	1
4	4	4	4	4	4	4	4
2	2	4	2	3	3	4	3
4	1	3	3	1	1	1	1
1	4	5	4	5	5	5	5
5	4	5	5	5	5	5	5
2	2	3	4	5	4	4	4
4	4	4	3	4	3	3	4
3	3	3	4	3	3	3	3
5	5	5	5	5	5	5	5
1	1	1	3	4	4	4	4
4	2	3	3	1	2	2	2
5	4	4	4	4	4	4	4
4	4	5	4	3	3	3	3
4	4	4	4	4	4	4	4
1	1	4	3	4	4	4	4
5	5	5	4	4	5	4	4
2	2	2	4	2	2	2	1
3	4	2	5	5	5	5	5
4	4	5	4	3	5	4	3
5	3	1	4	4	5	4	5

3	3	4	4	4	4	4	4
3	3	4	3	4	4	4	4
2	3	3	4	2	1	2	2
4	3	4	3	3	4	4	4
1	1	2	3	1	1	2	2
4	4	4	4	4	4	4	4
5	5	5	5	2	3	4	3
2	3	5	4	3	4	2	3
4	4	4	4	3	3	3	3
5	5	5	5	5	5	5	5
5	5	5	5	5	5	4	5
1	1	1	5	1	1	1	1
4	3	4	4	4	4	4	4
5	4	5	4	4	4	5	5
4	4	2	4	3	4	4	3
2	3	3	4	4	4	5	5
4	5	5	5	5	5	5	5
2	4	3	5	3	3	3	3
4	4	4	4	4	4	4	4
4	4	4	1	4	5	5	5
4	5	5	5	4	5	5	5
4	3	4	4	4	5	4	4
3	3	4	4	4	4	3	4
4	3	4	4	4	4	4	4
4	5	4	4	4	5	5	5
5	4	4	4	5	5	4	4
2	2	4	2	3	4	4	4
1	4	5	4	4	5	4	4
4	2	2	4	2	4	2	2
3	4	4	2	1	5	5	1
3	3	4	4	4	3	3	4
4	4	2	5	4	4	3	3
3	2	3	4	4	4	4	4
4	4	4	4	3	2	2	2
4	4	4	4	3	4	4	4
4	2	3	3	5	4	4	4
5	3	4	4	3	4	5	5
5	5	5	5	5	4	4	4
1	1	4	4	4	4	4	4
4	4	4	5	4	5	5	5
1	1	1	4	2	4	4	4
1	1	1	5	4	5	5	4
4	3	4	4	4	4	4	4
5	5	5	5	5	5	5	4
4	4	4	5	4	4	4	4
4	4	4	4	4	5	5	4
4	3	3	5	4	4	4	4

2	2	3	4	2	3	2	2
4	3	1	3	4	4	3	3
3	2	4	3	4	4	2	4
2	3	3	3	2	3	3	2
4	4	3	4	3	4	3	3
4	4	5	4	4	5	3	4
3	3	2	4	2	2	3	1
4	4	4	4	4	4	4	4
5	5	5	5	5	5	5	5
5	5	5	4	4	4	4	4
2	2	3	4	3	2	2	2
4	4	3	4	1	3	2	4
4	5	4	4	4	5	4	3
4	3	4	5	4	4	4	4
4	4	4	4	4	4	4	4
1	4	1	5	3	4	4	4
3	3	3	4	4	4	4	3
5	5	5	5	5	5	5	5
2	3	3	2	3	4	4	4
4	1	3	4	3	2	2	2
4	3	4	4	3	4	4	4
4	4	4	4	4	4	3	3
4	4	4	4	4	4	3	3
3	2	4	4	4	4	4	4
5	5	5	5	5	5	5	5
4	4	4	3	5	5	5	5
4	4	4	4	4	4	4	4
5	5	5	4	4	5	5	5
3	3	3	4	4	4	3	4
4	3	4	4	4	4	4	4
4	4	4	4	4	5	5	5
3	4	3	4	4	4	4	4
4	4	4	4	4	5	5	5
4	4	4	4	4	4	4	4
2	2	3	4	3	4	2	4
4	3	5	4	4	5	5	4
4	3	3	1	3	4	3	3
3	1	2	5	2	2	1	2
2	1	3	4	3	3	2	2
4	4	4	5	4	4	4	4
5	5	5	5	5	5	5	5
2	1	3	4	2	4	2	2
2	1	2	3	3	3	1	1
4	4	4	4	3	3	3	3
4	3	3	2	3	3	3	3
5	5	5	5	5	5	5	4
4	2	3	4	4	4	4	4

3	3	3	3	4	3	3	3
5	4	5	4	4	4	4	4
5	4	4	5	4	5	4	4
3	4	3	4	4	4	4	4
5	3	1	5	5	5	5	4
1	1	2	5	1	1	1	5
4	3	3	4	3	4	4	4
4	4	3	3	4	4	3	3
5	4	3	5	5	5	4	4
3	3	3	3	3	3	3	4
3	3	4	4	4	4	4	4
3	4	3	4	3	4	2	4
3	2	4	4	3	4	2	2
3	3	4	4	4	4	4	4
2	3	3	4	3	4	5	5
4	4	4	4	5	5	4	4
2	2	3	4	4	3	4	3
3	3	4	4	4	5	4	4
4	2	4	5	3	4	3	3
3	4	4	4	4	4	4	3
3	4	3	4	5	4	4	4
4	4	4	4	4	4	4	4
4	4	4	4	4	4	4	4
4	4	5	5	5	5	5	5
5	4	5	4	5	5	5	5
5	5	5	5	5	5	5	5
1	3	4	3	5	5	4	5
4	4	4	4	4	4	4	4
2	3	2	4	2	2	1	5
3	2	2	3	2	3	2	3
4	4	4	4	4	4	4	4
3	3	4	4	2	2	2	1
4	4	4	4	3	4	4	4
3	3	3	4	3	3	3	3
4	4	5	4	4	4	4	4
3	3	1	3	4	4	3	3
4	1	4	4	3	3	1	3
2	2	3	3	3	3	4	2
4	4	3	3	2	2	3	3
4	4	4	4	4	4	4	4
2	2	4	4	3	5	4	3
4	4	4	3	4	4	4	4
4	4	1	4	4	4	4	2
3	3	3	4	4	4	4	3
2	2	3	4	1	1	4	5
4	3	4	4	4	5	5	5
4	4	4	5	4	4	4	4

5	5	5	5	5	5	5	5
2	3	2	3	3	2	2	2
3	3	4	5	4	4	4	4
2	3	3	4	4	3	3	3
5	4	5	5	5	5	5	5
2	2	3	4	2	3	2	2
5	5	5	5	4	4	5	5
4	4	5	4	4	5	4	4
4	4	4	2	2	4	2	2
2	2	3	4	2	3	3	4
4	3	4	3	3	3	3	3
5	4	5	5	5	5	5	4
4	4	4	4	4	4	4	4
4	4	2	4	4	4	4	4
4	4	4	4	4	5	4	4
3	4	2	3	4	4	3	3
3	3	3	3	3	3	3	3
3	3	3	4	4	4	3	3
4	5	4	5	4	4	4	4
4	4	4	4	3	5	5	5
1	4	4	5	5	5	5	5
4	3	5	4	3	4	5	4
5	4	4	4	4	4	4	4
4	3	4	4	3	4	3	3
4	3	4	4	2	4	5	4
3	4	3	3	4	4	4	4
4	3	4	4	4	4	4	5
4	3	4	4	4	4	4	5
4	4	4	5	4	4	4	4
3	2	3	1	3	3	4	4
4	4	4	4	4	4	4	3
5	5	5	5	5	5	5	5
5	4	4	4	3	2	4	4
3	3	4	4	4	4	5	4
5	4	5	1	4	4	4	4
1	1	2	2	2	2	2	2
2	2	1	2	3	1	3	3
5	5	4	5	5	5	5	5
2	1	4	4	4	5	4	4
3	2	3	4	4	4	5	5
4	2	3	4	2	2	3	3
4	4	4	5	4	4	5	5
5	5	5	4	4	4	4	4
4	4	4	4	4	4	4	4
2	2	1	4	2	4	4	2
3	4	4	4	3	4	4	4
1	3	1	4	2	3	2	3

4	5	4	4	4	4	4	4
4	3	4	4	4	5	4	4
4	5	4	4	4	5	5	5
2	1	3	5	5	4	3	4
4	3	4	4	4	4	4	4
5	5	5	5	5	5	5	5
5	4	5	5	5	5	5	4
3	3	3	3	3	2	2	2
4	3	1	3	1	1	1	1
1	3	2	2	4	4	3	4
3	1	2	5	3	3	4	4
4	2	1	4	1	2	5	1
5	5	5	5	5	5	5	5
4	4	4	4	3	4	4	4
3	3	4	3	4	3	3	3
3	3	4	2	3	5	4	2
4	4	5	5	5	5	4	4
4	4	4	4	4	4	5	5
4	3	4	4	4	4	4	4
5	4	5	5	5	5	5	5
5	4	4	5	4	4	4	4
2	4	4	4	2	4	4	3
5	5	4	5	4	5	4	5
4	3	4	4	4	3	2	3
5	3	4	4	4	5	4	4
2	3	2	4	3	3	2	2
3	4	4	1	3	3	4	3
4	4	4	4	4	4	4	4
5	5	5	5	4	4	4	4
3	4	4	4	4	4	4	4
4	4	4	4	4	4	4	4
3	1	3	4	3	4	4	4
4	5	4	3	4	4	4	4
4	3	4	4	5	5	4	4
4	1	3	4	4	4	4	3
4	5	3	2	2	1	1	2
4	4	5	3	4	4	4	4
5	4	4	5	5	5	5	5
3	3	4	3	4	4	4	4
5	4	4	4	4	4	4	4
4	4	4	4	4	4	4	4
4	5	5	5	4	5	5	5
2	2	3	5	1	2	3	2
5	5	5	4	5	5	5	5
4	4	4	4	4	4	4	4
4	4	3	3	4	4	2	2
4	4	4	5	5	4	4	4

4	3	4	4	4	4	4	4
4	4	4	4	4	4	4	4
1	1	1	1	2	4	4	4
4	4	4	4	4	4	4	4
4	3	4	5	5	5	5	5
5	4	4	4	4	5	4	4
4	3	4	4	4	4	4	4
2	2	3	4	3	4	2	2
2	3	3	4	4	3	2	2
5	4	4	5	4	4	5	4
3	2	4	2	3	5	3	3
3	2	3	3	4	4	5	4
4	4	4	4	4	4	4	4
4	2	4	4	4	4	4	4
4	4	3	3	4	4	4	4
3	2	4	3	4	5	5	5
4	4	4	4	3	4	2	3
4	4	4	4	5	5	4	4
2	1	3	3	2	2	1	1
5	4	4	4	2	4	4	4
5	3	3	3	4	4	4	4
3	3	3	3	3	3	3	3
4	4	4	4	4	4	4	4
4	2	3	4	2	3	2	3
2	4	4	4	3	4	4	4
4	4	4	4	4	4	4	4
5	5	5	4	5	5	5	5
4	4	4	4	4	4	4	4
4	4	5	5	4	4	5	3
3	2	4	4	4	4	4	4
5	5	4	5	5	5	5	4
5	4	5	5	5	5	5	4
3	3	3	3	4	4	3	3
5	5	5	5	4	4	4	4
5	4	4	4	4	4	4	4
4	4	4	4	5	5	5	5
4	4	4	5	4	4	4	4
3	4	3	3	2	2	2	2
5	5	5	4	4	5	4	4
2	2	3	2	3	4	3	3
2	3	3	5	4	4	3	3
3	2	4	4	5	5	4	3
4	4	5	4	4	4	2	2
3	3	4	4	3	4	4	4
4	4	4	4	3	3	3	3
3	2	3	4	4	4	2	1
3	3	3	3	4	3	3	3

5	5	5	5	4	4	3	1
4	4	4	4	4	5	4	3
4	4	4	3	2	2	2	2
2	1	2	2	2	3	3	3
5	5	5	5	5	5	4	4
2	3	4	4	3	4	3	3
1	1	3	1	2	2	5	2
4	2	3	4	3	4	2	1
4	3	3	2	3	4	4	3
4	4	4	4	4	4	4	4
2	2	3	2	2	4	4	4
4	4	5	2	5	5	5	5
3	3	3	4	4	2	2	1
4	4	4	4	4	4	4	4
5	5	5	4	4	4	4	4
2	3	3	3	3	3	3	3
1	5	2	4	4	4	4	4
2	3	3	4	4	4	4	4
5	5	5	4	3	3	3	3
2	2	3	4	4	5	4	4
4	4	4	4	4	4	4	4
4	3	4	4	4	4	4	3
2	2	2	4	4	4	3	4
4	5	5	5	4	4	4	4
4	4	4	4	4	4	3	4
5	4	4	5	5	4	4	4
4	4	4	4	4	4	4	4
4	4	4	4	4	5	4	4
4	4	4	5	5	5	5	5
4	3	4	5	5	5	5	5
2	2	4	3	4	4	4	4
3	3	3	4	2	4	1	1
3	3	3	3	4	4	3	3
1	1	1	5	4	5	4	1
4	4	4	4	4	4	4	4
4	4	4	4	4	4	4	1
4	4	5	5	4	5	5	5
4	3	4	4	4	4	4	4
2	2	4	4	4	4	3	3
4	2	4	4	4	4	4	4
4	3	4	5	4	4	4	4
1	1	1	4	4	4	4	4
4	4	4	4	4	4	4	4
5	4	4	4	5	5	5	5
4	4	5	3	5	5	5	5
4	1	4	4	4	4	3	3
3	3	3	3	4	5	2	2

4	3	4	4	4	4	4	4
4	3	4	4	3	2	2	2
5	5	5	5	5	5	5	5
4	3	4	4	4	4	4	4
5	5	5	5	3	5	4	4
2	2	1	4	3	3	3	2
3	4	3	4	4	4	4	4
3	3	4	4	3	4	4	3
5	4	4	3	4	4	4	3
4	2	4	3	4	4	5	5
4	4	3	4	4	4	4	4
5	5	5	5	5	5	4	4
4	4	5	5	5	5	4	4
2	3	3	4	4	4	4	4
3	3	3	4	4	3	3	3
1	3	1	3	3	2	3	3
3	4	3	4	4	5	4	4
4	5	5	5	4	4	4	4
4	3	4	4	4	4	3	4
5	4	5	5	5	5	5	4
5	4	4	4	4	4	5	5
4	4	4	5	3	4	4	4
4	4	4	5	3	4	4	4
1	4	4	1	4	4	4	4
4	2	2	1	5	5	5	5
5	3	4	4	5	5	5	5
4	5	5	5	5	5	5	5
2	2	2	3	2	2	2	2
3	3	3	4	4	4	4	4
4	3	3	4	3	3	3	4
4	3	4	4	3	3	3	4
2	1	3	4	3	5	5	3
3	1	3	3	2	3	3	3
4	4	4	4	4	4	4	4
4	3	4	5	5	5	5	5
5	3	5	4	4	5	4	4
4	4	4	4	3	4	4	4
3	4	3	4	4	4	4	4
5	4	4	4	4	4	4	4
4	3	3	4	4	2	4	4
4	4	4	5	4	5	4	4
4	5	4	3	4	5	3	3
3	3	3	4	4	5	4	4
5	4	5	3	4	4	4	4
3	4	4	4	4	2	4	4
4	4	4	3	4	4	3	3
4	4	3	4	2	2	2	2

4	4	4	4	4	4	3	3
4	4	4	5	4	4	4	4
4	3	4	4	5	5	4	4
4	3	3	4	4	4	4	5
4	5	4	4	4	4	4	3
5	5	5	5	4	4	4	4
3	3	3	2	3	3	2	2
4	2	1	3	2	2	2	1
3	3	3	5	3	3	2	2
3	4	3	4	4	4	5	5
5	4	4	4	4	5	5	4
2	2	4	4	3	4	2	3
5	5	5	5	5	5	5	5
4	4	3	5	4	4	3	2
3	3	4	4	4	4	4	4
4	4	4	4	4	5	5	5
4	4	4	4	4	4	4	4
2	3	3	4	2	4	2	3
5	4	5	3	4	4	5	2
4	4	5	4	4	4	4	4
5	5	5	4	5	5	5	5
4	4	4	5	4	4	4	4
5	3	3	5	4	4	4	4
4	4	4	4	4	5	4	4
3	2	2	3	2	4	2	2
4	4	4	4	4	4	4	4
1	2	3	3	2	3	2	2
4	4	4	4	3	4	4	3
4	3	2	4	4	4	4	4
5	5	5	5	5	5	5	5
5	1	1	5	4	5	4	4
4	4	4	3	4	4	4	4
4	1	4	3	3	4	3	4
5	4	4	5	5	5	5	5
4	4	4	4	4	4	4	4
4	3	4	4	4	4	4	4
1	4	1	4	4	4	3	4
4	4	4	5	4	4	4	4
4	4	4	4	3	3	3	3
5	5	5	5	4	5	5	5
1	1	1	4	4	4	4	4
4	3	4	4	3	4	4	3
4	3	4	5	4	4	2	2
4	4	1	5	4	4	4	4
4	5	4	5	3	2	4	4
4	3	1	4	3	3	2	3
2	1	3	4	4	4	3	2

4	3	4	5	4	4	4	4
4	3	4	4	3	4	3	3
2	2	2	2	2	2	2	2
3	3	1	4	4	5	5	5
1	2	3	4	1	2	1	1
1	1	1	4	1	4	4	4
2	3	3	2	3	4	2	2
3	5	5	5	5	5	5	5
5	3	5	5	4	4	4	4
4	4	4	5	5	5	4	4
4	4	4	4	4	4	4	4
4	4	4	4	5	5	5	5
3	2	2	4	1	3	3	3
3	2	2	3	1	3	2	2
3	2	1	4	4	4	3	4
4	3	4	4	4	4	4	4
3	3	3	5	3	4	4	4
4	3	4	4	4	4	4	4
3	3	2	4	2	3	2	2
5	5	4	4	4	4	4	4
5	5	1	5	5	5	5	4
4	4	4	2	3	4	2	2
2	2	2	5	2	2	4	1
3	1	3	2	4	4	4	4
3	3	3	3	3	4	4	4
4	4	2	4	4	4	4	4
2	2	3	4	3	3	2	2
5	4	4	4	4	4	4	4
5	4	5	5	5	5	5	5
4	2	4	4	3	4	4	4
4	2	3	4	3	4	4	3
4	4	4	3	3	3	3	4
2	3	3	2	4	4	4	4
